# Supplementary material for: Large differences in carbohydrate degradation and transport potential among lichen fungal symbionts
Source: Nat Commun. 2022 May 12;13:2634. doi: 10.1038/s41467-022-30218-6 (PMC9098629; doi:10.1038/s41467-022-30218-6)
Supplement: Supplementary file 1 — Supplementary Information [file 41467_2022_30218_MOESM1_ESM.pdf]

Supplementary material for: Large differences in carbohydrate degradation and transport potential in the genomes of lichen fungal symbionts.

Philipp Resl   Adina R. Bujold   Gulnara Tagirdzhanova   Peter Meidl
Sandra Freire Rallo   Mieko Kono   Samantha Fernandez-Brime
Hörður Guðmundsson   Ólafur Sigmar Andrésson   Lucia Muggia
Helmut Mayrhofer   John P. McCutcheon   Mats Wedin   Silke Werth
Lisa M. Willis   Toby Spribille

12 April, 2022

Contents

Supplementary Methods **1**
Overview of analysis workflow . . . 1
Dataset construction . . . 1
De-novo sequencing of LFS genomes . . . 1
Raw sequencing data cleaning and filtering . . . 3
De-novo assembly, quality assessment and filtering . . . 4
Gene-calling and functional annotation . . . 4
Phylogenomic analyses and ultrametric tree reconstruction . . . 5
Selection of plant cell wall degrading enzymes . . . 5
Ancestral size and evolutionary dynamics of CAZyme families . . . 5
Overall similarity of CAZyme sets . . . 6
Additional characterization of carbohydrate active enzymes . . . 7
Heterologous expression and enzymatic assays of putative LFS cellulases . . . 7
Orthologue identification of cloned cellulases . . . 8
Identification of sugar and sugar-alcohol transporters . . . 8
Identification of fungal peroxidases . . . 8
Supplementary Table 1: Genomes included in this study . . . 9
Supplementary Table 2: Sample information of *de-novo* sequenced genomes . . . 12
Supplementary Table 3: Used software . . . 15
Supplementary Table 4: Read trimmers and parameters . . . 16
Supplementary Table 5: Genome assemblers and parameters . . . 17
Supplementary Table 6: Studied CAZyme families . . . 17
Supplementary Table 7: Overview of single-copy genes and alignments used for phylogenomic reconstruction. . . . 18
Supplementary Table 8: Gene family expansion analysis summary for different runs . . . 41
Supplementary Table 9, 10 and 11: Comparison of CAZyme number of LFS groups and other fungi 43
Supplementary Figure 1: Metrics of studied genomes . . . 45
Supplementary Figure 2: BUSCO completeness of studied genomes . . . 45
Supplementary Figure 3 and 4: Phylogenomic trees . . . 47
Supplementary Figure 5: Distribution of sugar-transporter orthologues . . . 49
Supplementary Figure 6: Distribution of peroxidase orthologues . . . 50

Supplementary Figure 7: Ancestral state reconstruction results for (hemi-)cellulose degrading CAZymes 51
Supplementary Figure 8: Ancestral state reconstruction results for pectin degrading CAZymes . . 52
Supplementary Figure 9: Ancestral state reconstruction results for lignin degrading CAZymes . . . 53
Supplementary Figure 10: Similarity of CAZyme sets based on PCA 54
Supplementary Figure 11: Distribution and ancestral states of all CAZyme families 56
Supplementary Figure 12: Overview of gene family expansion analyses results 61
Supplementary Figure 13: Tree of putative invertases in GH32 62
Supplementary Figures 14-61: Gene trees of CAZyme families generated with Saccharis 63

References **110**

Supplementary Methods

Overview of analysis workflow

The analysis workflow used to acquire the results in this paper, including all custom python and R scripts is available on Github (https://github.com/reslp/LFS-cazy-comparative). The workflow used to calculate phylogenomic trees is available on Github (https://github.com/reslp/phylociraptor) as well as the workflow used for gene prediction and functional annotation of the genomes (https://github.com/reslp/smsi-funannotate). An overview of the used software including version numbers is given in Supplementary Table 3.

Dataset construction

This study is based on the analysis of 83 fungal genomes (Supplementary Table 1). The selected genomes span six classes of Ascomycetes with focus on the largest radiation of lichen-fungal symbionts (LFS) Lecanoromycetes and their sister group Eurotiomycetes. We provide 29 *de-novo* sequenced draft genomes of LFSs and *Agyrium rufum*, which is a saprotrophic member of the Lecanoromycetes. Additional genomes include members of Dothideomycetes, Leotiomycetes, Sordariomycetes, Coniocybomycetes and Arthoniomycetes and cover species with different lifestyles including saprotrophs, pathogens, necrotrophs and symbionts. Our study also includes published genomes of LFS genomes from the Lecanoromycetes, Arthoniomycetes and Eurotiomycetes (Supplementary Table 1). For Eurotiomycetes, which are the sister-group to Lecanoromycetes, we included two members of each order (Supplementary Table 1).

De-novo sequencing of LFS genomes

We report the draft genome sequences of 29 *de-novo* sequenced Lecanoromycete genomes. Genomes were sequenced between 2014 and 2019 with different sequencing technologies and from different starting material: Some are derived from axenic cultures of the fungal component of lichens while others have been extracted from sequenced whole-lichen metagenomes. Voucher information, and DNA extraction method is summarized in Supplementary Table 2.

Agyrium rufum, *Lambiella insularis*, *Trapelia coarctata*, *Xylographa parallela*

Axenic mycobiont cultures were obtained from hymenial fragments of freshly collected lichen material on Malt-Yeast extract agar following the protocol of.[1] Tissue samples from axenic cultures of these four species were snap frozen at -80C and ground in a Retsch TissueLyser (Qiagen) immediately preceding nucleic acid extractions. We extracted DNA with the PowerBiofilm DNA Isolation Kit (MO BIO) and purified with Agencourt AMPure magnetic beads (Beckman Coulter) following manufacturers instructions. A minimum of 1μg of genomic DNA was used for library preparation. PCR-free libraries were prepared at the High Throughput Genomics Lab of the Huntsman Cancer Institute, Salt Lake City, UT with a mean insert size of 350 bp. These libraries were sequenced at the High Throughput Genomics Lab of the Huntsman Cancer Institute (Salt Lake City, UT, USA) on an Illumina HighSeq 2500 machine to 100 bp paired-end reads.

Lobaria immixta*, *Pseudocyphellaria aurata*, *Xylographa opegraphella*, *Xylographa soralifera*, *Xylographa bjoerkii*, *Xylographa pallens* and *Xylographa trunciseda

We extracted DNA from lichen fragments of *Lobaria immixta* and *Pseudocyphellaria aurata* and from axenic culture material of *Xylographa bjoerkii*, *X. opegraphella*, *X. pallens*, *X. soralifera* and *X. trunciseda* using a modified chloroform-phenol extraction method developed by the lab of Paul Dyer, University of Nottingham. We used about 2 cm^2 cleaned terminal pieces of the lichens or with pea-sized pieces of axenic culture as staring material, respectively.

1) 5PRIME Phase Lock Gel Light (hereafter: PLG; QuantaBio) tubes were centrifuged for 30s at 12,000 g immediately prior to use.
2) Then, 0.5 ml of DNA extraction buffer (room temperature; 250 mM Tris-HCl pH 8.5, 250 mM NaCl, 25 mM NaEDTA, 0.5% SDS) was added to the PLG tubes.
3) Freeze-dried material was ground thoroughly under liquid nitrogen in a mortar with a pistil and added to the DNA extraction buffer in the PLG tube.
4) One equal volume (0.5 ml) of ice-cold phenol:chloroform:isoamyl alcohol (25:24:1) was added to the ground mycelium in DNA extraction buffer, mixing thoroughly by inversion for 2 minutes, followed by centrifugation at 12,000 g at 4°C for 10 min.
5) The upper, aqueous phase was removed to a fresh tube, taking care not to disturb the interphase. 5 μL of RNAse (20 mg ml^{-1}) were added, incubating for 30 min at 37°C. Then, 1 μL of proteinase K was added to a final concentration of 10 μg/ml, incubating 40 min at 37°C. Finally, 1 volume (0.5 ml) of ice cold chloroform:isoamyl alcohol (24:1) was added, the solution was mixed thoroughly and centrifuged at 4°C and 12,000 g for 10 min. The upper (aqueous) phase was transferred to a new tube.
6) 0.54 volumes of isopropanol (-20°C) were added and mixed by inversion. The solution was left for 20 minutes on ice to precipitate the DNA.
7) The solution was centrifuged for 20 min at 4°C and 12,000g to pellet the DNA and the supernatant was discarded after centrifugation.
8) 500 μL 70% ethanol (-20°C) were added to the pellet and the pellet was washed by gentle vortexing. Then, the solution was centrifuged at 4°C and 12,000 g for 5 minutes before removing the supernatant. Another wash step was performed.
9) The pellet was air dried at room temperature for 15-20 min, then the pellet was resuspended in 100 μL TE buffer (10 mM Tris-HCl, 1 mM EDTA, pH 8.0) overnight at 4°C. The extracted DNA was subsequently cleaned using Agencourt AMPure magnetic beads (Beckman Coulter) following manufacturer's instructions. Library preparation was performed by the Research Center for Molecular Medicine of the Austrian Academy of Sciences (CEMM) using TrueSeq Nano (Illumina) Library Preparation kits. The seven prepared libraries were sequenced on a single lane of an Illumina HighSeq 3000 machine to 150 bp paired-end reads.

Loxospora ochrophaea*, *Schaereria dolodes

We extracted DNA using the DNeasy Plant Mini Kit (Qiagen, GmbH, Hilden, Germany) and prepared metagenomic libraries using TruSeq DNA PCR-Free Low Throughput Library Prep Kit (Illumina, San Diego, CA, USA). The libraries were sequenced by the Huntsman Cancer Center (Salt Lake City, UT, USA) on an Illumina HiSeq 2500 to 125 bp paired-end reads.

Ptychographa xylographoides*, *Xylographa vitiligo

We extracted DNA from thallus parts indicated in Supplementary Table 2 using the DNeasy Plant Mini Kit (Qiagen, GmbH, Hilden, Germany). Libraries were prepared using NEBNext Ultra II DNA Library Prep Kit (New England BioLabs, Ipswich, MA, USA) and sequenced by the Genome Sciences Centre (BC Cancer, Vancouver, BC, Canada) on an Illumina HiSeq X to 150 bp paired-end reads.

Acarospora aff strigata*, *Bachmanniomyces* sp. S44760, *Hypocenomyce scalaris*, *Icmadophila ericetorum*, *Lignoscripta atroalba*, *Mycoblastus sanguinarius*, *Puttea exsequens*, *Thelotrema lepadinum*, *Toensbergia leucococca*, *Varicellaria rhodocarpa*, *Xylographa carneopallida

We extracted DNA from thallus parts indicated in Supplementary Table 2 using the QIAamp Investigator kit (Qiagen, GmbH, Hilden, Germany) following the blood and tissue protocol. Libraries were prepared using NEBNext Ultra II DNA Library Prep Kit (New England BioLabs, Ipswich, MA, USA) and sequenced by the Genome Sciences Centre (BC Cancer, Vancouver, BC, Canada) on an Illumina HiSeq X to 150 bp paired-end reads.

Stictis urceolatum

We extracted DNA from apothecia collected from field-collected thalli of *Stictis urceolatum* using the CTAB method. The apothecia were homogenized in 4 % CTAB buffer (4% w/v CTAB; 100 mM Tris-HCl (pH 8.0); 20 mM EDTA (pH 8.0); 1.4M NaCl) and incubated at 60 °C for six hours constantly mixed on a tube rotator. After the incubation the homogenate was mixed with an equal volume of chloroform:isoamyl alcohol (24 : 1, v/v) and an upper aqueous phase was collected after centrifugation. After repeating this step twice, DNA in the aqueous phase was precipitated by isopropanol precipitation and dissolved in 1 x TE buffer. The DNA solution was treated with 0.2 mg/mL RNaseA (QIAGEN, Hilden, Germany). DNA was then purified by isopropanol and PEG precipitation (13 % w/v PEG 8000; 1.6 M NaCl) and dissolved in 0.1 x TE buffer. Library preparation and sequencing was done by SciLifeLab (Solna, Stockholm, Sweden) using SMARTer ThruPLEX DNA-seq kit (Takara Bio, Shiga, Japan) on an Illumina Miseq v3 (300 bp paired-end reads).

Peltigera leucophlebia* and *Sticta canariensis

DNA was extracted from *Peltigera leucophlebia* apothecia cleaned of algae on the lower surface and for a chloromorph thallus of *Sticta canariensis* as follows: Samples were ground in a mortar with a pistil after immersion in liquid nitrogen for 2.5 minutes. The resulting fine powder was placed in a 2 ml tube and 450 μL lysis buffer (250 mM Tris-HCl pH 8.5, 250 mM NaCl, 25 mM NaEDTA, 0.5% SDS) and 4 μL RNAse A (20 mg/ml) were added. The sample was mixed by inversion and placed at -50°C for 20 min. Then, the tubes were spun at maximum speed (12000g) for 25 minutes at 20°C. The resulting supernatant (375 μL) was pipetted into 2 ml collection tubes. Next, 55 μL binding buffer (2M guanidine hydrochloride in 95% EtOH) were added and the sample was mixed well by pipetting and releasing the entire volume. The sample was added to a 3μm glass fiber plate (Chromafil Multi 96 GF 3 μm, Cat. Nr. 738658.M, Macherey-Nagel), spun for 5 min at 3800 g at 20°C, discarding the flow-through. Then, two washing steps followed where 500 μL of 70% EtOH were pipetted into the glass fiber plate wells and the plate was spun for 3 min at 3800 g at 20°C. After this, the plate was centrifuged for 15 min at 3800g and 20°C to remove residual ethanol. Now, the glass fiber plate was placed on a PCR plate and 55 μL TE elution buffer were added (5 mM Tris pH 8.0, 0.05mM EDTA) and the plate was spun for 1 min at 3800 g and at 20°C. The elution was repeated as above to obtain a total of 110 μL DNA. The sequencing libraries were prepared using the Nextera XT DNA Library Prep Kit according to manufacturers instructions and sequenced using the Illumina Miseq v3 platform generating 300 bp paired-end reads.

Raw sequencing data cleaning and filtering

Raw paired-end read data of the *de-novo* sequenced genomes were cleaned using trimmomatic after inspection with FastQC. Trimming parameters and the used trimmomatic versions are summarized in Supplementary Table 4. Trimmed read files were inspected again with FastQC. Only trimmed reads were used for subsequent genome assembly.

De-novo assembly, quality assessment and filtering

We used different *de-novo* genome assemblers to assemble the trimmed reads of different species. For a small subset of species consisting of *Xylographa parallela*, *X. pallens*, *X. bjoerkii*, *X. soralifera*, *X. trunciseda* and *X. opegraphella* we created a number of test assemblies to investigate the impact of different assemblers on

common genome metrics such as N50, BUSCO completeness etc. For these species we assembled trimmed reads using SPAdes, Platanus, Minia, Abyss and Velvet. The quality of the generated test assemblies was assessed using QUAST and by comparing completeness of fungal (fungi_odb9) and ascomycete (ascomycota_odb9) BUSCOs using BUSCO 3. We used the best assemblies for the species included in our initial tests. We realized that SPAdes and Abyss produced consistently good assemblies in terms of BUSCO completeness and N50 while also being fast. Consequently we used either SPAdes or Abyss to assembly all *de-novo* sequenced genomes and the genome of *Graphis scripta*, for which only Illumina reads are available at the Sequence Read Archive (SRA) but no assembly. For each *de-novo* assembled genome the assembler and used parameters are summarized in Supplementary Table 5.

De-novo assembled genomes were filtered using blobtools as implemented in the binner pipeline (https://github.com/reslp/binner) after assembly to filter out contaminant non-fungal contigs. After this first filtering step, genome completeness was assessed using QUAST and BUSCO 3. Blobtools filtering worked well for most species, however for *Bachmanniomyces sp. S44760*, *Puttea exsequens*, *Toensbergia leucococca* and *Mycoblastus sanguinarius* we had to use additional binning methods. *Mycoblastus sanguinarius* was binned using CONCOCT as implemented in the binner pipeline. The remaining three species (*Bachmanniomyces sp. S44760*, *Puttea exsequens*, *Toensbergia leucococca*) were binned using CONCOCT within the metaWRAP pipeline; the lecanoromycete genomes were identified and assessed by screening the bins with BUSCO 4 in the automated lineage selection mode.

Published assemblies downloaded from NCBI contain only the nuclear genome. Consequently we also limited our downstream analyses to nuclear genomes. After the initial binning steps (see above) we filtered out candidate mitochondrial contigs in each *de-novo* sequenced genome based on BLAST similarity to published mitochondrial genes and complete mitochondrial genomes of *Graphis lineola* (Accession KY460674) and *Cladonia rangiferina* (Accession KY315996).[2] We selected these two species because they are members of the two large sub-groups of lichen-forming fungi Ostropomycetidae (*Graphis lineola*) and Lecanoromycetidae (*Cladonia rangiferina*). Using ncbi-blast+, we created BLAST databases for each binner filtered *de-novo* assembly and blasted all mitochondrial genes and genomes of *G. scripta* and *C. rangiferina* against the databases. Next we filtered the best BLAST hits for each query with an e-value cutoff of 1e-03 and a minimum alignment length of 500 base pairs to remove spurious hits. We extracted the target sequence names of these hits and filtered the input assemblies to remove all contigs containing hits from the query sequences. The so filtered genome assemblies were used in all downstream analyses. An overview of common genome metrics for each genome is given in Supplementary Figure 1.

Gene-calling and functional annotation

The quality and completeness of gene models and functional annotations can vary greatly. Well-studied species often have reference genomes with high-quality, manually curated gene models and comprehensive functional annotations. For the majority of organisms however, high quality assemblies, gene models and annotations are still lacking. In our study we combined heterogenic genomic data from a range of different sources, generated with different sequencing technologies at different times. This could introduce bias, potentially influencing the number of predicted genes, the quality of functional annotations and thus affecting results of our study. To reduce potential bias of inconsistent gene calling and functional annotation to a minimum, we used the same gene calling and functional annotation regime for all included genomes, regardless of them being publicly available (and thus already having functional annotations) or *de-novo* sequenced. This ensures both comparability and reproducability of the results presented in this study. For all genomes we used the funannotate pipeline (https://github.com/nextgenusfs/funannotate) to predict genes and provide functional annotations. Funannotate provides wrapper scripts around several important steps of genome annotation and produces output which can easily be incorporated into downstream analyses. First we cleaned raw assemblies with *funannotate clean* to remove duplicated (percent identity $> 95\%$) or too short (length $<$ 500bp) contigs from subsequent annotation steps. Next we sorted the contigs in each assembly by length using *funannotate sort*. After that, we masked repetitive regions in the assemblies with *funannotate mask* using custom generated repeat libraries with RepeatModeler and RepeatMasker. Repeatmasked assembles were then subjected to gene-calling using the gene-callers Augustus, GeneMark-ES, snap and GlimmerHMM. tRNAs were predicted using tRNAScan. The so generated gene-models were subjected to searches against

various databases to subsequently add functional annotations. Specifically we searched the predicted gene models against the InterPro databses using InterProScan and the PFAM database (version 33.1; Apr. 2020) using HMMer. Carbohydrate active enzymes (CAZymes) were predicted by HMMer against the dbCAN database version 9 (08 Apr. 2020; http://bcb.unl.edu/dbCAN2/) as part of the *funannotate annotate* step. Additional functional annotations were recovered from the EggNOG (version 4.5.1; 24 Mar. 2020; http://eggnog5.embl.de/) database. An overview of the number of called genes in each genome and annotated CAZymes is given in Supplementary Figure 1.

Phylogenomic analyses and ultrametric tree reconstruction

We used phylociraptor for all steps of phylogenomic tree inference: For the initial phylogenomic estimation we used all single-copy BUSCO genes of the ascomycota_odb9 set found in at least 80% of genomes. An overview of BUSCO results are given in Supplementary Figure 2. For each gene we created MAFFT alignments of the amino-acid sequence using the -auto flag. We trimmed alignments using trimAL with the -gappyout flag. This resulted in 1310 alignments (Supplementary Table 7), which were used for subsequent phylogenomic reconstruction. For each single-gene alignment we calculated the best substitution model using IQ-Tree's modeltest functionality and a maximum-likelihood tree. Using phylociraptor we created a concatenated alignment from all alignments and reconstructed a tree again with IQ-Tree. We performed a partitioned analysis using the best substitution model estimated for each gene.

As one measure of node support we calculated 1000 replicates of ultra fast bootstrap approximations.[3] Since bootstrap has been identified to overestimate node support in phylogenomic datasets[4] we also provide gene concordance and site concordance as two additional measures of node-support.[5] Gene concordance factors describe the proportion of genes supporting a node based on individual gene-trees, while site concordance factors describe the proportion of informative sites in the alignments supporting a node.[5] We calculated gene concordance and site concordance factors with IQ-Tree Additionally, we created a species tree using ASTRAL from the single-locus maximum-likelihood trees. We visualized all trees with custom R scripts (Supplementary Figures 3 and 4).

To calculate an ultrametric tree necessary for ancestral state reconstructions and gene family expansion analyses we used r8s to transform the branch lengths of the concatenated ML tree topology. We fixed the age of the tree root to an arbitrary value of 1000 (fixage taxon=root age=1000) and estimated the divergence times of descending nodes with a combination of penalized likelihood[6] and truncated network method (divtime method=PL algorithm=TN). The smoothing parameter for the penalized likelihood approach was set to 500 (set smoothing=500). This ultrametric tree was used for downstream analyses.

Selection of plant cell wall degrading enzymes

The degradative potential of plant cell wall degrading enzymes (PCWDEs) of fungi is manifold. Previous genomic studies suggest that breakdown of common plant cell-wall components can involve many gene families acting in concertation[7] [8]. We followed these studies on PCWD capabilities of fungi to select gene families potentially involved in degrading the three most common plant cell wall components (hemi)cellulose, pectin and lignin (Supplementary Table 6).

Ancestral size and evolutionary dynamics of CAZyme families

To study the evolution of CAZyme families involved in PCWD we reconstructed ancestral family sizes along our phylogenomic tree. We used gene family count information of each CAZyme family size involved in (hemi-)cellulose and pectin degradation as well as lignin modification as inferred by funannotate for each species. We were specifically interested in nodes close to the evolution of major lichen fungal symbiont groups. We thus reconstructed the ancestral size of CAZyme families for the root of the tree (R), the node of the Eurotiomycete and Lecanoromycete split (ELS), the ancestral node of Eurotiomycetes (AE), the node of the most recent common ancestor of Lecanoromycetes *sensu lato* (including *Acarospora* and *Umbilicaria*; ALSL), the node of the most recent common ancestor of Lecanoromycetes *sensu stricto* (ALSS), the node at the split between the two large subclasses Ostropomycetidae and Lecanoromycetidae (OLS), the MRCA nodes of

Lecanoromycetidae (AL) and Ostropomycetidae (AO) as well as the MRCA of the genus *Xylographa* (AX). For ancestral state reconstruction we used the anc.ML method from phytools[9] under an Ornstein-Uhlenbeck model of trait evolution.[10]

As a second approach and without restricting ourselves to *a-priori* defined sets of genes we analyzed all CAZyme families with CAFE. This allowed us to identify gene families with significantly elevated birth-rates, regardless of their ability to degrade different complex carbohydrates. We used CAZyme family counts from funannotate and our ultrametric phylogenomic tree as input for CAFE. We imposed two models, one with a single gene-birth rate parameter for the whole tree and one with two independent birth rates; one for Lecanoromycetes and one the rest of the tree. The second model was introduced to test the assumption that some gene families, playing an important role in Lecanoromycetes, could also have significantly different birth-rates and thus a model assuming a single birth rate could be too simplistic. For each birth-rate scenario we also estimated a specific error model with CAFE. We ran the CAFE analysis five times for each birth-rate parameter model, with and without the specific error model. This lead us to 20 total CAFE runs (Supplementary Table 8). We used custom R and python scripts to visualize expanded gene families and in how many CAFE runs the family was identified as significantly expanded ($p<0.05$) (Supplementary Table 8, Supplementary Figure 12).

Overall similarity of CAZyme sets

After investigating CAZyme families in an evolutionary context, we were further interested in the overall similarity of extant CAZyme sets. Gene numbers for each species and each CAZyme family included in the pectin and (hemi-)cellulose sets of CAZymes as well as CAZymes of the lignin set combined with the estimated numbers DyP- and Halo-Heme-peroxidases (see below) were log transformed and subjected to phylogenetically informed PCA,[11] as implemented in the phytools function *phyl.pca*. We used a Maximum-Likelihood optimization method (opt="ML") and the mod="corr" parameter. To be able to compare overall similarity of extant species and the CAZyme compositions based on ancestral state reconstruction for the nine nodes along the tree backbone (see above) we additionally performed a classical PCA. Since we did not estimate ancestral states for Dyp- and Halo-Heme-peroxidase counts we excluded them from the classical PCA. Again we used log-transformed gene-count information for each CAZyme family of each species and reconstructed ancestral nodes and calculated a PCA using the R function *prcomp* with the parameter center=T. We visualized the first two principal components for each PCA in R using ggplot2[12] (Supplementary Figure 10). We also compared the mean numbers of CAZymes in the five CAZyme categories (AA, CBM, GH, GT and PL) between Lecanoromycetes groups and other fungi. First we calculated mean values of CAZyme counts for each CAZyme groups for all Lecanoromycetes, Lecanoromycetidae, the five members of the OG clade (see Fig.1 in the main text) and Ostropomycetidae (without OG clade members) and all other fungi. Next we calculated the difference of these values between LFS groups and all other fungi as follows:

$$\frac{|LFSgroup - OtherFungi|}{\frac{LFSgroup+OtherFungi}{2}} * 100 = \%Difference$$

Differences in % are summarized in (Supplementary Table 9).

Next we compared the distributions of mean values for different LFS groups from above with a Wilcoxon rank-sum test in R to other fungi (Supplementary Table 10).

Additionally we calculated the percent increase (positive numbers) or decrease (negative numbers) of CAZyme numbers between LFS groups and all other fungi (Supplementary Table 11):

$$\frac{LFSgroup - OtherFungi}{OtherFungi} * 100 = \%Increase$$

Additional characterization of carbohydrate active enzymes

To characterize carbohydrate active enzymes beyond presence and absence in different genomes, we used a phylogenetic approach in combination with subcellular localization prediction and information of experimentally characterized enzymes listed in the cazy.org database. We started with downloaded information about experimentally characterized CAZymes by parsing the cazy.org website using a custom python script. This information contains broad taxonomic assignments (on the level of domains) as well as GenBank accession numbers, as well as descriptions of the activity of enzymes including Enzyme Code (EC) numbers. Cazy.org contains a large number of characterized CAZymes and CAZymes can act on many substrates apart from plant cell walls. This is especially important for some of the key families involved in degrading cellulose and hemicellulose, families GH5 and GH43. GH5 was originally called the "cellulase family"[13] however subsequent analyses revealed a large number of different enzymatic activities besides cellulase activity.[14] Similarly GH43, a family involved in hemicellulose and pectin breakdown which is often expanded in PCWD organisms[15] has been divided in many subfamilies to increase accuracy of predicted functions.[16]

To characterize functions of CAZymes in greater detail while taking into account potential multifunctionality within CAZyme families we blended information on characterized genes from each gene family listed in Supplementary Table 6 with results from Saccharis. Saccharis is a phylogenetic pipeline specifically developed for CAZyme characterization. It takes CAZyme families as input and downloads all characterized sequences listed in cazy.org from GenBank. Then it searches the predicted protein sequences from our included genomes against HMM profiles (from dbcan) of the specified family to identify additional sequences with affinity to the CAZyme family under study. The so-identified sequences are aligned together with the downloaded characterized sequences using MUSCLE and a phylogenetic tree is produced with FastTree based on this alignment.

Since plant-cell wall degrading enzymes have to act outside the fungal cell to have access to the long-chained carbohydrate substrates we predicted the subcellular location of CAZyme sequences using DeepLoc. Using protein sequences as input DeepLoc predicts the probability of an enzyme to be located in each of ten different subcellular locations. It is pre-trained on a large data set of UNIPROT sequences for which subcellular locations are known. We ran DeepLoc on all sequences assigned to a single CAZyme family by Saccharis including all sequences from characterized genes. We filtered DeepLoc results and kept location predictions only when probability for one location was above 70%. Finally we summarized information retrieved from cazy.org with the trees generated with Saccharis and subcellular location predictions as well as taxonomic assignments. We created a custom R script to visualize phylogenetic tree plots for each CAZyme family (Figure 3 in the main text; Supplementary Figure 13 and subsequent Supplementary Figures here).

Heterologous expression and enzymatic assays of putative LFS cellulases

Cellulase candidate selection: We found that all characterized sequences in GH5 subfamily 5 have Cellulase activity (EC 3.2.1.4) and the vast majority come from eukaryotes (see Figure 3 in the main text). We aligned the cellulase domain of multiple GH5 subfamily 5 sequences from different *Xylographa* species to a previously epxressed and crystalized cellulase domain (PDB: 3QR3, https://www.rcsb.org/structure/3QR3) from *Trichoderma reesei*. We selected *Xylographa* for this experiment due to the obligate lichen-forming lifestyle and the obligate and close association with decaying wood of all members of this genus. Based on initial alignments using Clustal Omega we selected two candidate genes from *Xylographa bjoerkii* whith high similarity to 3QR3 for heterologous expression experiments. These genes are subsequently referred to cellulase A and B.

Cloning and expression: Synthetic genes of cellulase A and B were sub-cloned from pET28a into pMBPT via PCR amplification, SalI/NotI double-digestion, ligation, and transformation into *E. coli* BL21. Transformants were confirmed by sequencing. Cultures were routinely grown on LB agar or in LB broth (Fisher Scientific, Mississauga, ON) containing 100 μg/mL ampicillin. For expression, overnight cultures seeded from a single colony isolate were used to inoculate 600 mL LB broth with ampicillin, which was then grown at 37ºC with 180 rpm shaking until an OD600nm of ~0.4-0.6 was reached. Cultures were then induced with 0.5 mM IPTG and incubated for 24 h at 20ºC with 180 rpm shaking. Cells were harvested at 10,000 $\times$g for 15 min at 4ºC and stored at -80ºC until protein purification.

Cell lysis: Frozen cell pellets were mixed with an equal amount of diatomaceous earth, 1 uL Benzonase, and a protease inhibitor tablet (Roche), then ground manually by mortar and pestle for several minutes until homogeneous. Buffer A (100 mM Tris, 200 mM NaCl, pH 5.5) was added one mL at a time until a volume of 10 mL/g pellet was achieved. The mixture was transferred to a 50 mL conical tube and centrifuged at 10,000 ×g for 10 min at 4ºC to pellet debris. The supernatant was filtered through a 0.45 μm syringe-top filter to remove residual particulate matter prior to purification.

Protein purification: Filtered cell lysates were loaded onto a 5 mL MBPT column at approximately 1.5 mL/min by syringe, then the column was washed with 3 column volumes (CV) of buffer A. The protein was eluted from the column in 1.5 mL fractions with 1 CV of a 50% mixture of buffer A and buffer B (100 mM Tris, 200 mM NaCl, 10 mM maltose, pH 5.5), followed by 3 CV of 100% buffer B. The fraction containing the highest concentration of the target protein was determined visually by SDS-PAGE and confirmed by Western blot with anti-His-HRP.

Enzyme assay optimization: Enzyme activity was tested at pH 3, 4, 5, 6, or 7; and incubated at 4, 20, 37, 50, or 60ºC for 24, 48, or 72 h in a flat-bottom plate. Absorbance at 595 nm was measured by plate reader and blanked with a sample containing water instead of enzyme. Only optimal pH range and incubation time were presented.

Orthologue identification of cloned cellulases

To identify orthologues of the cloned cellulases we inferred orthogroups for the protein files produced with funannotate with Orthofinder. Orthofinder uses a hybrid approach based on sequence similarity estimated by a blast all-vs-all search with diamond and subsequent reconstruction of gene trees and a species tree to identify (single copy) orthologs and paralogs.[17] The trees utilized by Orthofinder were reconstructed using Fasttree. The two cloned sequences were recovered in a single orthogroup. We used custom python and R scripts to count and visualize the number of orthologues for each speciesn in this orthogroup.

Identification of sugar and sugar-alcohol transporters

Apart from the ability to break down PCW material, fungi also need the ability to transport breakdown products across the cell membrane to be able to metabolize them. To identify sugar transporter orthologues in LFSs we took all genes with PFAM annotations for sugar transporters (PF00083) and combined them with the seed set of characterized sugar transporters from the PFAM database. The PFAM seed set consists of 33 experimentally characterized sequences from a wide range of different organisms and covers transporters for different sugars. Additional information can be found here: http://pfam.xfam.org/family/sugar_tr.

To this initial set of sequences we added experimentally characterized fungal cellodextrin (*Aspergillus niger*: MH648002.1 (NCBI); *Penicillium oxalicum*: S8AIR7 (UniProtKB)) and sugar-alcohol (*Ambrosiozyma monospora*: AAX98668.1; Debaryomyces hansenii: CAR65543.1, CAG86001.1; *Saccharomyces cerevisiae*: NP_010036.1) transporter sequences. Again we used Orthofinder to assign all sugar transporters to orthogroups and used the presence of characterized sequences in each orthogroup to assign putative functions to sequences from the 83 genomes under study. We used custom python and R scripts to count and visualize the number of orthologues in each sugar-transporter orthogroup (Supplementary Figure 5).

Identification of fungal peroxidases

To our knowledge peroxidases have not been surveyed in LFSs so far despite their importance in lignin modification in fungi[7,8,15] and their recently discovered unexpectedly high phylogenetic diversity.[18] To acquire a complete picture of the degradation potential of LFSs fungi we also identified a range of different peroxidases including class II peroxidases, Heme-peroxidases and CyP-peroxidases involved in lignin breakdown in the 83 genomes studied here. First we downloaded all 1290 Ascomycota peroxidase sequences from RedOxiBase (accessed Jul. 14 2021). Next, we used diamond 0.9.22 to search all characterized sequences against all predicted proteins from our genomes. We used the diamond results to subsample predicted proteins from the studied genomes to only those sequences with a diamond hit to a characterized sequence. Next we used Orthofinder on the reduced protein sequence sets and all characterized peroxidase sequences to cluster them

into orthogroups. We used the presence of characterized sequences in individual orthogroups to assigned names to orthogroups. Finally we used custom python and R scripts to count and visualize the number of orthologues in each peroxidase orthogroup (Supplementary Figure 6). Additionally we summed up all six types of Heme- and CyP peroxidases and added these numbers as a new column (DyP_haeme_peroxi_total) to Supplementary Figure 6. This column is also displayed in Figure 1 of the main text.

Supplementary Table 1: Genomes included in this study

Supplementary Table 1: List of fungal genomes analyzed in this study. This information is also provided in the Supplementary-Data-1 file in EXCEL format.

isolate	lifestyle	published	origin	Genbank or SRA accession	class or subclass
Acarospora aff strigata	lichen symbiont	this study	whole lichen	GCA_022814355.1	Lecanoromycetidae
Agyrium rufum	saprotroph	this study	axenic culture	GCA_022814335.1	Ostropomycetidae
Arthonia radiata	lichen symbiont	Armstrong et al. (2018)	NCBI Genbank	GCA_002989075.1	Arthoniomycetes
Aureobasidium pullulans	saprotroph	Zhang et al. (2017)	NCBI Genbank	GCA_003336255.1	Dothideomycetes
Botrytis cinerea	necrotroph	Staats et al. (2012)	NCBI Genbank	GCA_000143535.4	Leotiomycetes
Capronia coronata	saprotroph	Teixeira et al. (2017)	NCBI Genbank	GCA_000585585.1	Eurotiomycetes
Ceratocystis platani	biotroph (pathogenic)	Sayari et al. (2021)	NCBI Genbank	GCA_000978885.1	Sordariomycetes
Cetradonia linearis	lichen symbiont	Allen et al. (2018)	NCBI Genbank	GCA_003521265.1	Lecanoromycetidae
Cladonia grayi	lichen symbiont	Armaleo et al. (2019)	NCBI Genbank	DOE JGI Portal - version 3	Lecanoromycetidae
Cladonia macilenta	lichen symbiont	Park et al. (2013)	NCBI Genbank	GCA_000444155.1	Lecanoromycetidae
Cladonia metacorallifera	lichen symbiont	Park et al. (2014)	NCBI Genbank	GCA_000482085.2	Lecanoromycetidae
Cladonia uncialis	lichen symbiont	Bertrand et al. (2018)	NCBI Genbank	GCA_002927785.1	Lecanoromycetidae
Cladophialophora carrionii	biotroph (pathogenic)	Teixeira et al. (2017)	NCBI Genbank	GCA_000365165.2	Eurotiomycetes
Colletotrichum graminicola	biotroph (pathogenic)	Oconnell et al. (2012)	NCBI Genbank	GCA_000149035.1	Sordariomycetes
Coniella lustricola	saprotroph	Raudabaugh et al. (2018)	NCBI Genbank	GCA_003019895.1	Sordariomycetes
Coniochaeta ligniaria	saprotroph	Jimenez et al. (2017)	NCBI Genbank	GCA_001879275.1	Sordariomycetes
Cyanodermella asteris	biotroph (endophyte)	Jahn el al. (2017)	NCBI Genbank	GCA_900618795.1	Ostropomycetidae
Diaporthe longicolla	biotroph (pathogenic)	Li et al. (2015)	NCBI Genbank	GCA_000800745.1	Sordariomycetes
Dibaeis baeomyces	lichen symbiont	McDonald et al. (2013)	Sequence Read Archive	SRX665192	Ostropomycetidae
Elaphomyces granulatus	biotroph (mycorrizha)	Quandt et al. (2015)	NCBI Genbank	GCA_002240705.1	Eurotiomycetes
Endocarpon pusillum	lichen symbiont	Wang et al. (2014)	NCBI Genbank	GCA_000464535.1	Eurotiomycetes
Epichloe typhina	biotroph (pathogenic)	Schardl et al. (2013)	NCBI Genbank	GCA_000308955.1	Sordariomycetes
Erysiphe necator	biotroph (pathogenic)	Jones et al. (2014)	NCBI Genbank	GCA_000798715.1	Leotiomycetes
Evernia prunastri	lichen symbiont	Meiser et al. (2017)	NCBI Genbank	GCA_003184365.1	Lecanoromycetidae
Fonsecaea erecta	biotroph	Vicente et al. (2017)	NCBI Genbank	GCA_001651985.1	Eurotiomycetes
Fusarium oxysporum	biotroph (pathogenic)	Ma et al. (2010)	NCBI Genbank	GCA_000149955.2	Sordariomycetes
Graphis scripta	lichen symbiont	McDonald et al. (2013)	Sequence Read Archive	SRX665273	Ostropomycetidae
Gomphillus americanus	lichen symbiont	Tagirdzhanova et al. (2021)	NCBI Genbank	GCA_905337335.1	Ostropomycetidae
Gyalolechia flavorubescens	lichen symbiont	Park et al. (2013)	NCBI Genbank	GCA_000442125.1	Lecanoromycetidae
Helicocarpus griseus	saprotroph	Munoz et al. (2018)	NCBI Genbank	GCA_002573585.1	Eurotiomycetes
Hypocenomyce scalaris	lichen symbiont	this study	whole lichen	GCA_022814315.1	Lecanoromycetidae
Hypoxylon pulicicidum	biotroph (endophyte)	Nicholson et al. (2018)	NCBI Genbank	GCA_002775035.1	Sordariomycetes

Supplementary Table 1: List of fungal genomes analyzed in this study. This information is also provided in the Supplementary-Data-1 file in EXCEL format. *(continued)*

isolate	lifestyle	published	origin	Genbank or SRA accession	class or subclass
Icmadophila ericetorum	lichen symbiont	this study	whole lichen	GCA_022814295.1	Ostropomycetidae
Knufia petricola	saprotroph	Tesei et al. (2017)	NCBI Genbank	GCA_002319055.1	Eurotiomycetes
Lambiella insularis	lichen symbiont	this study	axenic culture	GCA_022814265.1	Ostropomycetidae
Lasallia hispanica	lichen symbiont	DalGrande et al. (2018)	NCBI Genbank	GCA_003254425.1	Lecanoromycetidae
Lasallia pustulata	lichen symbiont	Wang et al. (2017)	NCBI Genbank	GCA_900169345.1	Lecanoromycetidae
Bachmanniomyces sp. S44760	lichen symbiont	this study	whole lichen	GCA_022814255.1	Ostropomycetidae
Lignoscripta atroalba	lichen symbiont	this study	whole lichen	GCA_022814235.1	Lecanoromycetidae
Lobaria immixta	lichen symbiont	this study	whole lichen	GCA_022814215.1	Ostropomycetidae
Loxospora ochrophaea	lichen symbiont	this study	axenic culture	GCA_022814175.1	Lecanoromycetidae
Magnaporthe grisea	biotroph (pathogenic)	Song et al. (2018)	NCBI Genbank	GCA_002871045.1	Sordariomycetes
Malbranchea cinnamomea	saprotroph	Huttner et al. (2017)	NCBI Genbank	GCA_900128795.2	Leotiomycetes
Monascus purpureus	saprotroph	Yang et al. (2015)	NCBI Genbank	GCA_003184285.1	Eurotiomycetes
Mycoblastus sanguinarius	lichen symbiont	this study	whole lichen	GCA_022814195.1	Ostropomycetidae
Onygena corvina	saprotroph	Huang et al. (2015)	NCBI Genbank	GCA_000812245.1	Eurotiomycetes
Peltigera leucophlebia	lichen symbiont	this study	whole lichen	GCA_022814155.1	Lecanoromycetidae
Penicillium chrysogenum	saprotroph	Specht et al. (2014)	NCBI Genbank	GCA_000710275.1	Eurotiomycetes
Phaeomoniella chlamydospora	biotroph (pathogenic)	MoralesCruz et al. (2015)	NCBI Genbank	GCA_001006345.1	Eurotiomycetes
Phialophora attae	biotroph (ant symbiont)	Moreno et al. (2015)	NCBI Genbank	GCA_001299255.1	Eurotiomycetes
Phyllosticta citricarpa	biotroph (pathogenic)	Wang et al. (2016)	NCBI Genbank	GCA_001604955.1	Dothideomycetes
Podospora anserina	saprotroph	Espagne et al. (2008)	NCBI Genbank	GCA_000226545.1	Sordariomycetes
Pseudevernia furfuracea	lichen symbiont	Meiser et al. (2017)	NCBI Genbank	GCA_003184345.1	Lecanoromycetidae
Pseudocyphellaria aurata	lichen symbiont	this study	whole lichen	GCA_022814125.1	Lecanoromycetidae
Pseudophaeomoniella oleicola	biotroph	MoralesCruz et al. (2015)	NCBI Genbank	GCA_003868215.1	Eurotiomycetes
Ptychographa xylographoides	lichen symbiont	this study	whole lichen	GCA_022814105.1	Ostropomycetidae
Puttea exsequens	lichen symbiont	this study	whole lichen	GCA_022814085.1	Lecanoromycetidae
Ramalina intermedia	lichen symbiont	Wang et al. (2018)	NCBI Genbank	GCA_003073195.1	Lecanoromycetidae
Ramalina peruviana	lichen symbiont	Merlotti et al. (2018)	NCBI Genbank	GCA_001956345.1	Lecanoromycetidae
Schaereria dolodes	lichen symbiont	this study	whole lichen	GCA_022814065.1	Ostropomycetidae
Sordaria macrospora	saprotroph	Nowrousian et al. (2010)	NCBI Genbank	GCA_000182805.2	Sordariomycetes
Sticta canariensis	lichen symbiont	this study	whole lichen	GCA_022814045.1	Lecanoromycetidae
Stictis urceolatum	lichen symbiont	this study	whole lichen	GCA_022814025.1	Ostropomycetidae
Talaromyces cellulolyticus	saprotroph	Fujii et al. (2015)	NCBI Genbank	GCA_000829775.1	Eurotiomycetes
Thelotrema lepadinum	lichen symbiont	this study	whole lichen	GCA_022813995.1	Ostropomycetidae
Thermoascus crustaceus	biotroph (pathogenic)	NCBI Genbank (strain JM 12817, sequenced by RIKEN BioResource Center and RIKEN Center for Life Science Technologies (Japan))	NCBI Genbank	GCA_001599835.1	Eurotiomycetes
Thielaviopsis musarum	necrotroph	Wingfield et al. (2015)	NCBI Genbank	GCA_001513885.1	Sordariomycetes

Supplementary Table 1: List of fungal genomes analyzed in this study. This information is also provided in the Supplementary-Data-1 file in EXCEL format. *(continued)*

isolate	lifestyle	published	origin	Genbank or SRA accession	class or subclass
Toensbergia leucococca	lichen symbiont	this study	whole lichen	GCA_022813965.1	Lecanoromycetidae
Trapelia coarctata	lichen symbiont	this study	axenic culture	GCA_022813945.1	Ostropomycetidae
Trichoderma reesei	saprotroph	Martinez et al. (2008)	NCBI Genbank	GCA_000167675.2	Sordariomycetes
Umbilicaria muehlenbergii	lichen symbiont	Park et al. (2014)	NCBI Genbank	GCA_000611775.1	Lecanoromycetidae
Uncinocarpus reesii	saprotroph	Sharpton et al. (2009)	NCBI Genbank	GCA_000003515.2	Eurotiomycetes
Varicellaria rhodocarpa	lichen symbiont	this study	whole lichen	GCA_022813925.1	Ostropomycetidae
Verticillium dahliae	biotroph (pathogenic)	Ma et al. (2010)	NCBI Genbank	GCA_000150675.2	Sordariomycetes
Xylaria grammica	saprotroph	Sook-Young et al. (2021)	NCBI Genbank	GCA_004353285.1	Sordariomycetes
Xylographa bjoerkii	lichen symbiont	this study	axenic culture	GCA_022813905.1	Ostropomycetidae
Xylographa carneopallida	lichen symbiont	this study	whole lichen	GCA_022813885.1	Ostropomycetidae
Xylographa opegraphella	lichen symbiont	this study	axenic culture	GCA_022813865.1	Ostropomycetidae
Xylographa pallens	lichen symbiont	this study	axenic culture	GCA_022813845.1	Ostropomycetidae
Xylographa parallela	lichen symbiont	this study	axenic culture	GCA_022813825.1	Ostropomycetidae
Xylographa soralifera	lichen symbiont	this study	axenic culture	GCA_022813805.1	Ostropomycetidae
Xylographa trunciseda	lichen symbiont	this study	axenic culture	GCA_022813785.1	Ostropomycetidae
Xylographa vitiligo	lichen symbiont	this study	whole lichen	GCA_022813765.1	Ostropomycetidae

Supplementary Table 2: Sample information of *de-novo* sequenced genomes

Supplementary Table 2: List of de-novo sequenced LFS genomes analyzed in this study. This information is also provided in the Supplementary-Data-2 file in EXCEL format.

isolate	extraction method	class or subclass	voucher	material	date	comments
Acarospora aff strigata	QIAamp DNA Investigator Kit	Lecanoromycetidae	Spribille 44754, Isolate T1882, Canada:Alberta,Donalda	10-15 thalli with apothecia	Mar. 31 2019	Lichen morphologically close to A. fuscata, but ITS places fungus within A. strigata in Westberg data set.
Agyrium rufum	PowerBiofilm DNA Isolation Kit	Ostropomycetidae	Spribille 39798, Canada: British Columbia, Gnat Pass	small pieces of mycelium	Aug. 13 2012	
Hypocenomyce scalaris	QIAamp DNA Investigator Kit	Lecanoromycetidae	Spribille 44763, Isolate T1881, Canada:Alberta, E of Hinton along Hwy 16	many individual squamules	Apr. 7 2019	
Icmadophila ericetorum	QIAamp DNA Investigator Kit	Ostropomycetidae	Spribille 43583, isolate T1913, Canada: Manitoba,HIghway 6 N of Grand Rapids	sterile thallus only	Jul. 4 2018	
Lambiella insularis	PowerBiofilm DNA Isolation Kit	Ostropomycetidae	Spribille 39820, USA: Montana near N end of Salmon Lake, Lincoln. Co.	cultured mycelium	Sep. 7 2012	
Bachmanniomyces sp. S44760	QIAamp DNA Investigator Kit	Ostropomycetidae	Spribille 44760, isolate T1894, Canada: Alberta, E of Hinton along Hwy 16	seven or eight apothecia	Apr. 7 2019	This is Lecidea scabridula Hedl. nom. illeg.
Lignoscripta atroalba	QIAamp DNA Investigator Kit	Lecanoromycetidae	Spribille 44772, isolate T1887, Canada: Alberta, Athabasca R near Brule	many apothecia	Apr. 7 2019	
Lobaria immixta	Phenol-Chloroform extraction	Ostropomycetidae	Silke Werth ST12-03b, Tenerife, ca. 3 km SW of La Esperanza along Road TF24. Lat: 28.43232, Lon: -16.39024.	not recorded	May 10 2018	
Loxospora ochrophaea	DNeasy Plant Mini	Lecanoromycetidae	Spribille 41460, isolates: T1860-1863 pooled, USA:New Hampshire, Kancamagus Highway	cultured mycelium	Jun. 26 2016	extraction performed in Missoula Spring 2017
Mycoblastus sanguinarius	QIAamp DNA Investigator Kit	Ostropomycetidae	Spribille 43910, isolate T1914, Canada: Saskatchewan, Wapawekka Hills	sterile thallus only	Jul. 07 2018	

Supplementary Table 2: List of de-novo sequenced LFS genomes analyzed in this study. This information is also provided in the Supplementary-Data-2 file in EXCEL format. *(continued)*

**isolate**	**extraction method**	**class or subclass**	**voucher**	**material**	**date**	**comments**
Peltigera leucophlebia	see relevant section in supplementary information document	Lecanoromycetidae	Silke Werth HD1-Pleu1, isolate: Pleu1 Iceland: Heidmoerk (Lat: 64.06833844, Lon: -21.72876066)	mycelium from the backside of apothecia	Aug. 18 2018	
Pseudocyphellaria aurata	Phenol-Chloroform extraction	Lecanoromycetidae	Silke Werth ST10-01b, Spain, Tenerife, Anaga Mountains. 28.5387161, Lon: -16.26701272	not recorded	May 9 2018	
Ptychographa xylographoides	DNeasy Plant Mini	Ostropomycetidae	Spribille 42058, isolate T1868, Canada: Alberta, Rock Lake		Sep. 25 2017	
Puttea exsequens	QIAamp DNA Investigator Kit	Lecanoromycetidae	Spribille 42807, isolate T1888, Canada: Alberta, W of Clear Prairie	many apothecia	Sep. 30 2017	
Schaereria dolodes	DNeasy Plant Mini	Ostropomycetidae	Spribille 41586, Spribille 41654, isolates T1822 and T1849 pooled, USA: Lake Co, Tim Wheeler residence, Jocko River swimming hole	not recorded	Jan.-Mar. 2017	
Sticta canariensis	Phenol-Chloroform extraction	Lecanoromycetidae	leg. Israel Perez Vargas, voucher S.can, Spain, Tenerife, Anaga Mountains, Lat: 28.54, Lon: -16.27	not recorded	Mar. 2 2017	
Stictis urceolatum	CTAB Method	Ostropomycetidae	USA: Virginia, Smyth County, Grindstone Campground, on hardwood, leg. Linda Phillips (S)	apothecia	May 1 2017	
Thelotrema lepadinum	QIAamp DNA Investigator Kit	Ostropomycetidae	Spribille 44606, isolate T1916 , Canada: British Columbia, Frisby Creek near Revelstoke	apotheica	Oct. 20 2018	
Toensbergia leucococca	QIAamp DNA Investigator Kit	Lecanoromycetidae	Spribille 42847, isolate T1904, Canada: Saskatchewan, Wapawekka Hills	many squamules	Apr. 28 2018	
Trapelia coarctata	PowerBiofilm DNA Isolation Kit	Ostropomycetidae	Resl 1158, isolate: P141, Austria: Styria, Schoeckl	small pieces of mycelium	Apr. 4 2012	

Supplementary Table 2: List of de-novo sequenced LFS genomes analyzed in this study. This information is also provided in the Supplementary-Data-2 file in EXCEL format. *(continued)*

**isolate**	**extraction method**	**class or subclass**	**voucher**	**material**	**date**	**comments**
Varicellaria rhodocarpa	QIAamp DNA Investigator Kit	Ostropomycetidae	Spribille 41938, isolate T1912, Canada: Northwest Territories, dolomite cliffs ca 8.5 km SW of Edzo	sterile thallus	Aug.17	
Xylographa bjoerkii	Phenol-Chloroform extraction	Ostropomycetidae	Spribille 41600, USA: Oregon, beach across from Neah-Kah-Nie School	cultured mycelium	Jan. 29 2017	
Xylographa carneopallida	QIAamp DNA Investigator Kit	Ostropomycetidae	Spribille 44761, isolate T1889, Canada: Alberta, E of Hinton along Hwy 16	10 - 15 apothecia and thallus	Apr. 07 2019	
Xylographa opegraphella	Phenol-Chloroform extraction	Ostropomycetidae	Spribille 41601, USA: Oregon, beach across from Neah-Kah-Nie School	cultured mycelium	Jan. 29 2017	
Xylographa pallens	Phenol-Chloroform extraction	Ostropomycetidae	Resl 1159, Austria: Stryria, Eisenerz, S of Praebichl, 400m after Praebichlerhof along hiking trail	small pieces of mycelium	Nov. 27 2012	
Xylographa parallela	PowerBiofilm DNA Isolation Kit	Ostropomycetidae	Resl 1145, isolate T1151, Austria: Carinthia, Hochrindl	small pieces of mycelium	Jun. 5 2013	
Xylographa soralifera	Phenol-Chloroform extraction	Ostropomycetidae	Spribille 41478, USA: Montana Silver Butte Pass	cultured mycelium	Aug. 15 2016	
Xylographa trunciseda	Phenol-Chloroform extraction	Ostropomycetidae	Spribille 41477, USA: Montana Silver Butte Pass	cultured mycelium	Aug. 15 2016	
Xylographa vitiligo	DNeasy Plant Mini	Ostropomycetidae	Spribille 42472, isolate T1866, Canada: Alberta, Swan Hills	soredia including wood grains	Oct. 28 2017	

Supplementary Table 3: Used software

Supplementary Table 3: Software used in this study.

software	version	step	citation
trimmomatic	0.38	data cleaning	Bolger (2014)
FastQC	0.11.7	data cleaning	Andrews (2010)
binner	0.1	data cleaning	Resl (2020)
SPAdes	3.12.0	assembly	Bankevich et al. (2012)
platanus	1.2.4	assembly	Kajitani et al. (2014)
minia3	git commit 1d5b8f4	assembly	Chiki and Rizk (2012)
abyss	2.0.1	assembly	Jackman et al. (2017)
velvet	1.2.0	assembly	Zerbino and Birney (2008)
QUAST	4.6.3	assembly	Gurevich et al. (2013)
BUSCO	3.0.2, 4.0.2	assembly/phylogenomics	Waterhouse et al. (2017)
blobtools	1.1.1	assembly filtering	Laetsch and Blaxter (2017)
MetaWrap	1.2	assembly filtering	Uritskiy et al. (2018)
CONCOCT	1.2	assembly filtering (within MetaWrap)	Alneberg et al. (2014)
ncbi-blast+	2.9.0	assembly filtering	Camacho et al. (2009)
funannotate	1.8.3	genome annotation	Palmer and Stajich (2021)
RepeatModeller	1.0.11	genome annotation	Smit el al. (2008)
RepeatMasker	4.0.7	genome annotation	Smit et al. (2013)
Augustus	3.3.2	genome annotation (within funannotate)	Stanke et al (2006)
GlimmerHMM	3.0.4	genome annotation (within funannotate)	Majoros et al. (2004)
snap	2006-07-28	genome annotation (within funannotate)	Korf (2004)
GeneMark-ES	4.62 (Jan. 2020)	genome annotation (within funannotate)	Ter-Hovhannisyan et al. (2008)
tRNA-Scan	2.0.5	genome annotation (within funannotate)	Lowe and Eddy (1997)
InterproScan	5.48-83.0	genome annotation	Jones et al (2014)
eggnog-mapper	1.0.3	genome annotation	Huerta-Cepas et al. (2017)
phylociraptor	git commit a93b4c8	phylogenomics	Resl and Hahn (2021)
mafft	7.464	phylogenomics (within phylociraptor)	Katoh and Standley (2013)
trimAL	1.4.1	phylogenomics (within phylociraptor)	Capella-Gutierrez et al. (2009)
IQ-Tree	2.0.7	phylogenomics (within phylociraptor)	Minh et al. (2020)
ASTRAL	5.7.1	phylogenomics (within phylociraptor)	Mirarab et al. (2014)
r8s	1.81	phylogenomics	Sanderson (2003)
CAFE	5.0.0b2	gene family evolution analysis	Mendes et al. (2020)
Saccharis	git commit 9a748be	cazyme characterization	Jones et al. (2018)
MUSCLE	3.8.31	cazy characterization (within Saccharis)	Edgar (2004)
hmmer	3.1b2	cazy characterization (within Saccharis)	Mistry et al. (2013)
raxml	8.2.12	cazy characterization (within Saccharis)	Stamatakis (2014)
DeepLoc	1.0	cazyme characterization	Almagro Armenteros et al. (2017)
Clustal Omega	1.2.4	cazy characterization	Sievers et al. (2011)
Orthofinder	2.5.2	orthology detection	Emms and Kelly (2019)
diamond	0.9.24, 0.9.22	orthology detecion (within Orthofinder)	Buchfink et al. (2021)
Fasttree	2.1.10	orthology detection (within Orthofinder)	Price et al. (2010)

Supplementary Table 4: Read trimmers and parameters

Supplementary Table 4: Software and parameters used in initial trimming of raw data of different species.

	trimmer	trimming__parameters
Acarospora__aff__strigata	Trimmomatic 0.38	ILLUMINACLIP:all__PE.fa:2:30:10 LEADING:30 TRAILING:30 SLIDINGWINDOW:4:15 MINLEN 80
Agyrium__rufum	Trimmomatic 0.35	LEADING:5 TRAILING:5 SLIDINGWINDOW:4:15 MINLEN:50
Dibaeis__baeomyces	Trimmomatic 0.35	ILLUMINACLIP:all__PE.fa:2:20:7:1:false LEADING:28 TRAILING:28 SLIDINGWINDOW:4:15 MINLEN:50
Graphis__scripta	Trimmomatic 0.35	ILLUMINACLIP:TruSeq2-PE.fa:2:30:10 LEADING:30 TRAILING:30 SLIDINGWINDOW:4:15 MINLEN:36
Hypocenomyce__scalaris	Trimmomatic 0.38	ILLUMINACLIP:all__PE.fa:2:30:10 LEADING:30 TRAILING:30 SLIDINGWINDOW:4:15 MINLEN 80
Icmadophila__ericetorum	Trimmomatic 0.38	ILLUMINACLIP:all__PE.fa:2:30:10 LEADING:30 TRAILING:30 SLIDINGWINDOW:4:15 MINLEN 80
Lambiella__insularis	Trimmomatic 0.38	ILLUMINACLIP:all__PE.fa:2:30:10 LEADING:30 TRAILING:30 SLIDINGWINDOW:4:15 MINLEN 80
Bachmanniomyces__sp.__S44760	Trimmomatic 0.38	ILLUMINACLIP:all__PE.fa:2:30:10 LEADING:30 TRAILING:30 SLIDINGWINDOW:4:15 MINLEN 80
Lignoscripta__atroalba	Trimmomatic 0.38	ILLUMINACLIP:all__PE.fa:2:30:10 LEADING:30 TRAILING:30 SLIDINGWINDOW:4:15 MINLEN 80
Lobaria__immixta	Trimmomatic 0.38	ILLUMINACLIP:all__PE.fa:2:30:10 LEADING:33 TRAILING:33 SLIDINGWINDOW:15:20 MINLEN:36
Loxospora__ochrophaea	Trimmomatic 0.38	ILLUMINACLIP:all__PE.fa:2:30:10 LEADING:30 TRAILING:30 SLIDINGWINDOW:4:15 MINLEN 80
Mycoblastus__sanguinarius	Trimmomatic 0.38	ILLUMINACLIP:all__PE.fa:2:30:10 LEADING:30 TRAILING:30 SLIDINGWINDOW:4:15 MINLEN 80
Peltigera__leucophlebia	Trimmomatic 0.36	ILLUMINACLIP:NexteraPE-PE.fa:2:30:10 LEADING:30 TRAILING:30 SLIDINGWINDOW:4:15 MINLEN 50
Pseudocyphellaria__aurata	Trimmomatic 0.38	ILLUMINACLIP:all__PE.fa:2:30:10 LEADING:33 TRAILING:33 SLIDINGWINDOW:15:20 MINLEN:36
Ptychographa__xylographoides	Trimmomatic 0.38	ILLUMINACLIP:all__PE.fa:2:30:10 LEADING:30 TRAILING:30 SLIDINGWINDOW:4:15 MINLEN 80
Puttea__exsequens	Trimmomatic 0.38	ILLUMINACLIP:all__PE.fa:2:30:10 LEADING:30 TRAILING:30 SLIDINGWINDOW:4:15 MINLEN 80
Schaereria__dolodes	Trimmomatic 0.38	ILLUMINACLIP:all__PE.fa:2:30:10 LEADING:30 TRAILING:30 SLIDINGWINDOW:4:15 MINLEN 80
Sticta__canariensis	Trimmomatic 0.38	ILLUMINACLIP:all__PE.fa:2:30:10 LEADING:33 TRAILING:33 SLIDINGWINDOW:15:20 MINLEN:36
Stictis__urceolatum	Trimmomatic 0.38	ILLUMINACLIP:all__PE.fa:2:30:10 LEADING:30 TRAILING:30 SLIDINGWINDOW:4:15 MINLEN:100
Thelotrema__lepadinum		
Toensbergia__leucococca	Trimmomatic 0.38	ILLUMINACLIP:all__PE.fa:2:30:10 LEADING:30 TRAILING:30 SLIDINGWINDOW:4:15 MINLEN 80
Trapelia__coarctata	Trimmomatic 0.35	LEADING:5 TRAILING:5 SLIDINGWINDOW:4:15 MINLEN:50
Varicellaria__rhodocarpa	Trimmomatic 0.38	ILLUMINACLIP:all__PE.fa:2:30:10 LEADING:30 TRAILING:30 SLIDINGWINDOW:4:15 MINLEN 80
Xylographa__bjoerkii	Trimmomatic 0.38	ILLUMINACLIP:all__PE.fa:2:30:10 LEADING:30 TRAILING:30 SLIDINGWINDOW:4:15 MINLEN 100
Xylographa__carneopallida	Trimmomatic 0.38	ILLUMINACLIP:all__PE.fa:2:30:10 LEADING:30 TRAILING:30 SLIDINGWINDOW:4:15 MINLEN 80
Xylographa__opegraphella	Trimmomatic 0.38	ILLUMINACLIP:all__PE.fa:2:30:10 LEADING:30 TRAILING:30 SLIDINGWINDOW:4:15 MINLEN 80
Xylographa__pallens	Trimmomatic 0.38	ILLUMINACLIP:all__PE.fa:2:30:10 LEADING:30 TRAILING:30 SLIDINGWINDOW:4:15 MINLEN 60
Xylographa__parallela	Trimmomatic 0.38	ILLUMINACLIP:all__PE.fa:2:30:10 LEADING:30 TRAILING:30 SLIDINGWINDOW:4:15 MINLEN 80
Xylographa__soralifera	Trimmomatic 0.38	ILLUMINACLIP:all__PE.fa:2:30:10 LEADING:30 TRAILING:30 SLIDINGWINDOW:4:15 MINLEN 100
Xylographa__trunciseda	Trimmomatic 0.38	ILLUMINACLIP:all__PE.fa:2:30:10 LEADING:30 TRAILING:30 SLIDINGWINDOW:4:15 MINLEN 60
Xylographa__vitiligo	Trimmomatic 0.38	ILLUMINACLIP:all__PE.fa:2:30:10 LEADING:30 TRAILING:30 SLIDINGWINDOW:4:15 MINLEN 80

Supplementary Table 5: Genome assemblers and parameters

Supplementary Table 5: Software and parameters used to assemble the genomes of different species.

	**assembler**	**parameters**
Acarospora__aff__strigata	SPAdes v.3.12.0	-k 21,31,51,71,81,101,127 –careful
Agyrium__rufum	SPAdes v.3.6.2	-k 21,33,55,77,89 –careful
Dibaeis__baeomyces	SPAdes v.3.6.2	-k 21,33,41,49 –careful
Graphis__scripta	SPAdes v.3.6.2	-k 21,33,55,77,89 –careful
Hypocenomyce__scalaris	SPAdes v.3.12.0	-k 21,31,51,71,81,101,127 –careful
Icmadophila__ericetorum	SPAdes v.3.12.0	-k 21,31,51,71,81,101,127 –careful
Lambiella__insularis	SPAdes v.3.12.0	-k 21,31,51,71,81,101,127 –careful
Bachmanniomyces__sp.__S44760	SPAdes v.3.12.0	-k 21,31,51,71,81,101,127 –careful
Lignoscripta__atroalba	SPAdes v.3.12.0	-k 21,31,51,71,81,101,127 –careful
Lobaria__immixta	SPAdes v.3.12.0	-k 31,43,55,67,79,91,103,115,127 –careful
Loxospora__ochrophaea	SPAdes v.3.12.0	-k 21,31,51,71,81,101,127 –careful
Mycoblastus__sanguinarius	SPAdes v.3.12.0	-k 21,31,51,71,81,101,127 –careful
Peltigera__leucophlebia	SPAdes v.3.12.0	-k 21,31,51,71,89 –careful
Pseudocyphellaria__aurata	SPAdes v.3.12.0	-k 31,43,55,67,79,91,103,115,127 –careful
Ptychographa__xylographoides	SPAdes v.3.12.0	-k 21,31,51,71,81,101,127 –careful
Puttea__exsequens	SPAdes v.3.12.0	-k 21,31,51,71,81,101,127 –careful
Schaereria__dolodes	SPAdes v.3.12.0	-k 21,31,51,71,81,101,127 –careful
Sticta__canariensis	SPAdes v.3.12.0	-k 31,43,55,67,79,91,103,115,127 –careful
Stictis__urceolatum	SPAdes v.3.12.0	-k 21,31,51,71,81,101,127 –careful
Thelotrema__lepadinum		
Toensbergia__leucococca	SPAdes v.3.12.0	-k 21,31,51,71,81,101,127 –careful
Trapelia__coarctata	SPAdes v.3.6.2	-k 21,33,55,77,89,101 –careful
Varicellaria__rhodocarpa	SPAdes v.3.12.0	-k 21,31,51,71,81,101,127 –careful
Xylographa__bjoerkii	Abyss 2.0.1	K=55
Xylographa__carneopallida	SPAdes v.3.12.0	-k 21,31,51,71,81,101,127 –careful
Xylographa__opegraphella	Abyss 2.0.1	K=65
Xylographa__pallens	Abyss 2.0.2	K=90
Xylographa__parallela	SPAdes v.3.12.0	-k 61 –careful
Xylographa__soralifera	Abyss 2.0.1	K=90
Xylographa__trunciseda	SPAdes v.3.12.0	-k 31 –careful
Xylographa__vitiligo	SPAdes v.3.12.0	-k 21,31,51,71,81,101,127 –careful

Supplementary Table 6: Studied CAZyme families

Supplementary Table 6: CAZyme families involved in degrading different PCW components studied here.

	Set of gene families
(hemi)cellulose	AA3, AA9, CBM1, CBM13, CBM35, CBM6, CBM66, GH10, GH1, GH11, GH12, GH141, GH16, GH2, GH26, GH27, GH29, GH30, GH3, GH31, GH35, GH36, GH39, GH43, GH45, GH5, GH51, GH55, GH6, GH61, GH67, GH7, GH72, GH74, GH95
pectin	CE8, GH105, GH28, GH49, GH53, GH79, GH88, PL1, PL3, PL4, PL9
lignin	AA1, AA2, AA5

Supplementary Table 7: Overview of single-copy genes and alignments used for phylogenomic reconstruction.

Supplementary Table 7: Overview of alignments of genes used for phylogenomic reconstruction. Table generated with phylociraptor. This information is also provided in the Supplementary-Data-3 file in EXCEL format.

gene	length	no. of sequences	no. of parsimony informative sites	no. of variable sites	no. of fixed sites	best model
EOG092D4D0M	150	78	54	89	61	DCMut+I+G4
EOG092D0GSP	963	81	442	889	74	LG+F+I+G4
EOG092D0VUY	671	69	364	621	50	LG+I+G4
EOG092D1179	819	82	434	648	171	JTT+F+I+G4
EOG092D11OI	1068	81	542	842	226	LG+I+G4
EOG092D1Q73	460	82	192	429	31	LG+I+G4
EOG092D09NC	1338	82	435	1088	250	JTT+F+I+G4
EOG092D1P7M	850	80	320	828	22	JTT+I+G4
EOG092D3CGZ	248	74	102	237	11	LG+F+I+G4
EOG092D0S24	1206	78	309	1140	66	JTT+F+I+G4
EOG092D3M5I	283	80	179	248	35	LG+I+G4
EOG092D2MTA	1418	78	466	1398	20	JTT+F+I+G4
EOG092D2A39	972	81	342	922	50	JTT+F+I+G4
EOG092D1SW4	422	82	235	337	85	LG+I+G4
EOG092D4CWZ	179	76	128	163	16	LG+F+I+G4
EOG092D01J4	2451	82	963	2413	38	JTT+F+I+G4
EOG092D3J3N	265	79	165	229	36	JTTDCMut+I+G4
EOG092D1ODS	436	83	253	404	32	LG+F+I+G4
EOG092D42U2	225	76	140	201	24	LG+I+G4
EOG092D2V2F	317	82	32	262	55	JTT+F+I+G4
EOG092D4NC2	121	81	86	98	23	LG+I+G4
EOG092D3TIT	627	80	163	585	42	LG+F+I+G4
EOG092D13U3	725	73	338	688	37	JTT+I+G4
EOG092D2GBR	378	67	231	347	31	LG+I+G4
EOG092D26GB	818	81	196	794	24	VT+F+I+G4
EOG092D4CNZ	273	64	146	261	12	JTT+F+I+G4
EOG092D3SSN	179	80	93	128	51	LG+I+G4
EOG092D1KAG	747	80	485	697	50	JTTDCMut+I+G4
EOG092D0T1H	820	76	506	720	100	LG+I+G4
EOG092D28JA	383	77	240	322	61	LG+F+I+G4
EOG092D2ZQ2	433	69	195	365	68	LG+F+I+G4
EOG092D1FF7	548	83	206	265	283	LG+I+G4
EOG092D0VMZ	703	83	394	660	43	LG+F+I+G4
EOG092D4HEW	193	83	102	166	27	LG+I+G4
EOG092D3ZQC	738	81	140	718	20	JTT+F+I+G4
EOG092D2MZB	393	81	211	354	39	LG+I+G4
EOG092D1S42	413	80	181	375	38	LG+I+G4
EOG092D4KES	139	70	21	64	75	LG+G4
EOG092D0GFU	958	72	653	911	47	LG+F+I+G4
EOG092D1KPD	549	81	246	341	208	LG+I+G4
EOG092D47B5	978	83	449	822	156	JTT+I+G4
EOG092D1SLW	428	82	196	346	82	LG+I+G4
EOG092D4D2W	178	77	126	171	7	LG+I+G4
EOG092D0DUF	1011	79	561	800	211	LG+I+G4
EOG092D4O4M	190	83	75	167	23	JTT+I+G4
EOG092D069S	1210	75	499	874	336	LG+F+I+G4
EOG092D2RN7	383	80	215	333	50	LG+I+G4
EOG092D172M	543	83	260	464	79	LG+I+G4
EOG092D24ND	672	74	299	646	26	WAG+G4
EOG092D2DN8	379	81	126	224	155	LG+I+G4
EOG092D2GPJ	470	76	210	412	58	LG+I+G4
EOG092D076U	1032	79	568	754	278	LG+I+G4
EOG092D0ELD	1809	81	416	1764	45	JTT+F+I+G4

Supplementary Table 7: Overview of alignments of genes used for phylogenomic reconstruction. Table generated with phylociraptor. This information is also provided in the Supplementary-Data-3 file in EXCEL format. *(continued)*

gene	length	no. of sequences	no. of parsimony informative sites	no. of variable sites	no. of fixed sites	best model
EOG092D3B56	715	81	152	588	127	JTT+F+I+G4
EOG092D0M7O	735	83	349	689	46	LG+F+I+G4
EOG092D3FK5	179	70	102	177	2	LG+G4
EOG092D0J6Y	766	75	367	570	196	JTT+I+G4
EOG092D4A2D	273	81	176	235	38	LG+I+G4
EOG092D0CVR	947	71	557	926	21	LG+F+I+G4
EOG092D0Q9D	640	81	306	432	208	LG+I+G4
EOG092D17F4	536	78	323	445	91	JTTDCMut+I+G4
EOG092D3PL7	400	83	193	334	66	LG+F+I+G4
EOG092D3D0W	503	78	96	455	48	JTT+F+I+G4
EOG092D3BKV	405	83	201	307	98	JTT+F+I+G4
EOG092D1GOQ	426	77	205	323	103	LG+I+G4
EOG092D07WZ	1021	77	749	921	100	LG+F+I+G4
EOG092D430X	488	81	256	444	44	JTT+F+I+G4
EOG092D4CHO	191	81	69	152	39	LG+I+G4
EOG092D4DWT	170	80	101	131	39	LG+G4
EOG092D1W8I	454	75	323	401	53	LG+I+G4
EOG092D3IB3	419	82	164	368	51	JTT+I+G4
EOG092D3ALG	297	82	170	251	46	LG+I+G4
EOG092D3JAI	492	82	162	470	22	JTT+I+G4
EOG092D23DO	653	82	118	612	41	JTT+F+I+G4
EOG092D3B18	253	74	145	238	15	LG+I+G4
EOG092D1U19	623	83	297	536	87	WAG+I+G4
EOG092D0QVE	906	82	341	714	192	JTTDCMut+F+I+G4
EOG092D4TKE	170	67	70	160	10	VT+I+G4
EOG092D09F6	970	74	565	692	278	LG+I+G4
EOG092D1YB4	569	77	333	517	52	JTT+F+I+G4
EOG092D2SHG	336	82	205	258	78	LG+I+G4
EOG092D2UBG	501	72	312	464	37	LG+F+I+G4
EOG092D1UUH	505	82	214	400	105	LG+I+G4
EOG092D2AVN	286	76	215	274	12	LG+I+G4
EOG092D1BFC	538	74	209	288	250	LG+I+G4
EOG092D0SFV	663	82	516	634	29	JTT+I+G4
EOG092D0UBQ	610	82	342	515	95	JTTDCMut+I+G4
EOG092D431N	180	67	112	155	25	VT+G4
EOG092D4380	176	81	113	139	37	LG+I+G4
EOG092D2N4T	500	82	326	443	57	LG+I+G4
EOG092D47GI	183	73	99	169	14	LG+G4
EOG092D0L00	836	82	439	814	22	JTT+F+I+G4
EOG092D0P1F	775	83	297	729	46	JTT+I+G4
EOG092D2DNW	607	80	254	569	38	LG+F+I+G4
EOG092D3VG3	300	79	186	262	38	LG+I+G4
EOG092D0NEP	969	72	392	948	21	LG+F+I+G4
EOG092D1LJK	704	81	248	601	103	JTT+F+I+G4
EOG092D1RDH	447	77	250	413	34	LG+I+G4
EOG092D2BJL	151	73	64	126	25	LG+I+G4
EOG092D3CQ9	348	78	150	326	22	JTTDCMut+I+G4
EOG092D4H8N	167	80	63	157	10	WAG+F+I+G4
EOG092D1KTN	429	70	243	374	55	LG+I+G4
EOG092D2W7U	332	82	89	269	63	JTT+G4
EOG092D1ULX	404	78	283	346	58	LG+I+G4
EOG092D4DTK	137	78	61	127	10	VT+I+G4
EOG092D3ECY	343	79	214	316	27	LG+F+I+G4
EOG092D011S	2334	82	644	2197	137	JTT+F+I+G4
EOG092D47YB	234	81	148	201	33	LG+I+G4
EOG092D01WH	1137	77	362	1128	9	JTT+F+I+G4

Supplementary Table 7: Overview of alignments of genes used for phylogenomic reconstruction. Table generated with phylociraptor. This information is also provided in the Supplementary-Data-3 file in EXCEL format. *(continued)*

gene	length	no. of sequences	no. of parsimony informative sites	no. of variable sites	no. of fixed sites	best model
EOG092D357K	408	82	245	353	55	LG+I+G4
EOG092D27ZM	390	81	77	191	199	LG+I+G4
EOG092D4C1H	145	54	62	129	16	LG+G4
EOG092D1NDG	398	80	196	295	103	LG+I+G4
EOG092D3VIZ	807	82	410	721	86	JTT+F+I+G4
EOG092D1T7G	748	82	418	643	105	JTT+I+G4
EOG092D2ZWY	322	74	177	275	47	LG+I+G4
EOG092D0JA2	750	77	522	651	99	LG+F+I+G4
EOG092D1L0N	506	79	294	415	91	LG+F+I+G4
EOG092D2MAK	693	79	447	675	18	LG+I+G4
EOG092D4H14	319	79	129	301	18	LG+I+G4
EOG092D3BBO	653	77	301	595	58	JTT+I+G4
EOG092D3OFG	401	79	162	340	61	JTTDCMut+F+I+G4
EOG092D2RP6	354	80	212	298	56	LG+I+G4
EOG092D04ZP	1166	81	570	829	337	LG+I+G4
EOG092D3RZ8	274	76	169	243	31	LG+F+I+G4
EOG092D4G83	223	79	100	207	16	JTT+I+G4
EOG092D3LRW	326	78	151	304	22	JTTDCMut+F+I+G4
EOG092D0NV0	663	76	361	631	32	JTT+I+G4
EOG092D13KG	862	81	319	782	80	LG+F+I+G4
EOG092D1FGG	511	82	180	259	252	LG+I+G4
EOG092D10DF	848	82	497	779	69	JTTDCMut+I+G4
EOG092D4JU7	554	76	143	546	8	VT+F+I+G4
EOG092D3LA8	330	80	96	288	42	LG+I+G4
EOG092D444G	150	73	93	125	25	WAG+I+G4
EOG092D3V1W	168	71	84	158	10	LG+I+G4
EOG092D07BL	1339	80	731	1136	203	LG+I+G4
EOG092D16TH	899	82	324	746	153	JTT+I+G4
EOG092D4AY8	160	77	123	140	20	LG+I+G4
EOG092D3XVK	216	78	67	128	88	LG+I+G4
EOG092D34ZI	401	79	180	344	57	JTT+F+I+G4
EOG092D2DQ7	428	80	242	366	62	LG+I+G4
EOG092D4XRX	903	83	411	897	6	JTT+F+I+G4
EOG092D3DNT	291	76	217	281	10	LG+G4
EOG092D4KVP	245	78	171	232	13	LG+I+G4
EOG092D070E	1507	83	739	1280	227	JTT+I+G4
EOG092D248P	555	83	291	463	92	LG+F+I+G4
EOG092D4RYQ	118	82	28	48	70	JTT+I+G4
EOG092D44BU	202	80	126	174	28	VT+I+G4
EOG092D4PUW	184	77	87	156	28	Dayhoff+F+I+G4
EOG092D33KV	437	76	203	378	59	LG+I+G4
EOG092D3IVF	336	81	158	297	39	JTT+F+I+G4
EOG092D2LES	430	82	285	387	43	LG+G4
EOG092D48ZU	185	71	79	142	43	VT+G4
EOG092D2VOM	299	82	124	204	95	LG+F+I+G4
EOG092D4H30	92	80	7	68	24	VT+G4
EOG092D0HE1	947	80	564	823	124	LG+F+I+G4
EOG092D2C76	799	78	392	756	43	JTT+I+G4
EOG092D434J	245	80	160	225	20	LG+I+G4
EOG092D4D4F	134	74	110	129	5	LG+I+G4
EOG092D1Q04	579	75	287	567	12	JTT+I+G4
EOG092D1V8V	432	83	114	383	49	JTTDCMut+I+G4
EOG092D0ITM	825	81	562	742	83	JTT+F+I+G4
EOG092D4MNU	177	76	74	134	43	LG+I+G4
EOG092D1DIJ	502	83	317	418	84	JTT+I+G4
EOG092D3PNL	402	72	237	381	21	JTT+I+G4

Supplementary Table 7: Overview of alignments of genes used for phylogenomic reconstruction. Table generated with phylociraptor. This information is also provided in the Supplementary-Data-3 file in EXCEL format. *(continued)*

gene	length	no. of sequences	no. of parsimony informative sites	no. of variable sites	no. of fixed sites	best model
EOG092D23WM	556	83	132	520	36	JTT+I+G4
EOG092D4N86	198	81	63	162	36	LG+I+G4
EOG092D12CU	672	81	218	339	333	LG+I+G4
EOG092D35K7	284	82	99	278	6	JTT+I+G4
EOG092D3UVA	468	83	339	458	10	LG+I+G4
EOG092D2DE1	350	78	213	274	76	LG+F+I+G4
EOG092D4KSS	113	52	61	85	28	LG+G4
EOG092D38OJ	268	79	182	256	12	JTTDCMut+I+G4
EOG092D2NK1	471	82	276	419	52	LG+F+I+G4
EOG092D1718	1493	81	264	1470	23	JTT+F+I+G4
EOG092D2A7Q	409	79	274	370	39	LG+I+G4
EOG092D2XJY	313	61	192	305	8	LG+I+G4
EOG092D4C59	157	81	100	122	35	LG+I+G4
EOG092D3KF0	428	82	196	389	39	LG+F+I+G4
EOG092D0590	1318	82	697	1201	117	JTT+F+I+G4
EOG092D0YSG	924	80	349	825	99	JTT+I+G4
EOG092D27VI	415	81	193	387	28	JTT+I+G4
EOG092D2U5N	545	80	142	533	12	JTT+I+G4
EOG092D4VBM	139	12	57	111	28	LG+F+G4
EOG092D2PEN	416	78	239	331	85	LG+I+G4
EOG092D2VEC	395	78	205	287	108	LG+I+G4
EOG092D39EO	298	76	204	283	15	LG+I+G4
EOG092D28EY	1131	82	450	1070	61	JTT+F+I+G4
EOG092D3FDW	298	79	205	284	14	LG+I+G4
EOG092D3GMS	111	76	48	67	44	WAG+G4
EOG092D1LTM	530	81	385	483	47	LG+F+I+G4
EOG092D0YW3	665	79	231	560	105	JTT+I+G4
EOG092D43MS	186	79	75	139	47	LG+I+G4
EOG092D35VJ	480	82	207	371	109	JTTDCMut+F+I+G4
EOG092D2ICY	354	78	125	220	134	WAG+I+G4
EOG092D1ZZP	504	77	345	435	69	LG+I+G4
EOG092D2UWC	308	79	172	250	58	LG+I+G4
EOG092D4OXW	113	82	48	83	30	LG+I+G4
EOG092D2XOE	611	81	184	569	42	LG+I+G4
EOG092D3J4Y	316	82	153	294	22	WAG+I+G4
EOG092D28BN	605	69	136	570	35	LG+I+G4
EOG092D2W27	301	77	203	263	38	JTT+F+I+G4
EOG092D0KUS	778	78	483	673	105	JTT+I+G4
EOG092D4FS0	281	80	139	226	55	LG+I+G4
EOG092D3TIC	433	73	103	410	23	WAG+I+G4
EOG092D1H5T	557	72	210	408	149	LG+I+G4
EOG092D16KC	848	81	375	833	15	JTT+I+G4
EOG092D2N6R	191	78	101	170	21	WAG+I+G4
EOG092D1RUY	584	81	360	540	44	LG+F+I+G4
EOG092D3NXJ	852	79	219	749	103	JTT+F+I+G4
EOG092D1QLK	828	82	414	608	220	LG+I+G4
EOG092D4MRW	119	78	38	81	38	JTT+G4
EOG092D05RI	1736	81	447	1679	57	JTT+F+I+G4
EOG092D0KJL	1155	82	532	1084	71	JTT+F+I+G4
EOG092D04CD	1240	80	240	1217	23	JTT+F+I+G4
EOG092D0OMH	724	79	209	669	55	LG+F+I+G4
EOG092D3HJ3	276	82	114	148	128	LG+I+G4
EOG092D2FMD	401	73	300	364	37	LG+I+G4
EOG092D29G7	373	81	181	291	82	LG+F+I+G4
EOG092D2MH0	474	79	226	421	53	LG+I+G4
EOG092D38AL	601	79	233	493	108	JTT+F+I+G4

Supplementary Table 7: Overview of alignments of genes used for phylogenomic reconstruction. Table generated with phylociraptor. This information is also provided in the Supplementary-Data-3 file in EXCEL format. *(continued)*

gene	length	no. of sequences	no. of parsimony informative sites	no. of variable sites	no. of fixed sites	best model
EOG092D2ZRK	330	68	233	321	9	LG+F+G4
EOG092D1L48	905	83	211	843	62	JTT+F+I+G4
EOG092D1OKZ	530	70	199	393	137	LG+I+G4
EOG092D4RG5	89	77	64	71	18	LG+I+G4
EOG092D2VEA	387	76	207	378	9	JTT+I+G4
EOG092D3ASB	314	80	189	301	13	LG+I+G4
EOG092D3M1U	364	80	201	284	80	JTT+I+G4
EOG092D0XCU	1007	74	316	992	15	JTT+F+I+G4
EOG092D0RST	988	82	614	940	48	LG+F+I+G4
EOG092D092D	748	63	436	729	19	LG+I+G4
EOG092D3SJS	821	75	131	807	14	JTT+I+G4
EOG092D2UCZ	226	80	120	191	35	LG+I+G4
EOG092D0F10	1056	49	653	983	73	LG+I+G4
EOG092D4CBH	184	70	112	171	13	LG+I+G4
EOG092D2RAL	313	78	153	268	45	LG+G4
EOG092D10ZS	703	81	347	605	98	JTT+I+G4
EOG092D1G5D	589	75	308	497	92	LG+F+I+G4
EOG092D10UA	698	82	379	613	85	LG+I+G4
EOG092D1QZM	511	77	262	444	67	LG+I+G4
EOG092D1VUE	671	81	236	562	109	LG+F+I+G4
EOG092D3V2Z	331	78	164	318	13	LG+F+I+G4
EOG092D1H9Y	440	82	247	393	47	LG+I+G4
EOG092D128P	993	78	370	986	7	LG+I+G4
EOG092D3BUG	360	80	170	326	34	JTT+I+G4
EOG092D4124	288	78	162	271	17	LG+I+G4
EOG092D3M40	574	79	281	530	44	JTT+F+I+G4
EOG092D2D20	687	83	94	640	47	JTT+F+I+G4
EOG092D20CT	791	74	452	765	26	JTT+I+G4
EOG092D3MB4	244	77	92	231	13	LG+F+I+G4
EOG092D2A6H	439	75	294	410	29	LG+I+G4
EOG092D19A4	696	82	212	308	388	LG+I+G4
EOG092D3S7I	148	76	51	145	3	JTT+I+G4
EOG092D3T80	200	83	116	152	48	LG+I+G4
EOG092D1RSN	675	80	407	609	66	JTT+I+G4
EOG092D3OBO	336	68	154	314	22	LG+I+G4
EOG092D1A3F	586	83	330	520	66	JTT+I+G4
EOG092D4CO8	228	80	120	223	5	JTTDCMut+I+G4
EOG092D48Q8	180	76	109	153	27	LG+I+G4
EOG092D2AGS	504	81	211	441	63	LG+I+G4
EOG092D4BKP	178	83	97	132	46	JTT+I+G4
EOG092D0RR7	1027	68	745	1009	18	JTT+F+I+G4
EOG092D4JAO	234	80	98	220	14	LG+F+I+G4
EOG092D45TL	352	69	158	348	4	JTTDCMut+F+I+G4
EOG092D3XGU	220	73	139	216	4	LG+I+G4
EOG092D0PYM	751	83	267	479	272	LG+I+G4
EOG092D1UKL	637	79	265	522	115	JTT+I+G4
EOG092D07ZA	1058	76	581	907	151	LG+I+G4
EOG092D4F81	244	74	128	227	17	LG+F+I+G4
EOG092D3XC6	247	82	130	200	47	Dayhoff+I+G4
EOG092D2N6E	340	80	122	201	139	LG+I+G4
EOG092D1RV3	708	83	256	673	35	JTTDCMut+F+I+G4
EOG092D0HKD	1117	83	245	932	185	JTT+I+G4
EOG092D3T99	307	79	171	290	17	JTTDCMut+I+G4
EOG092D4ENZ	282	81	185	271	11	LG+I+G4
EOG092D4CP0	265	82	179	242	23	JTTDCMut+I+G4
EOG092D0UX9	718	80	363	482	236	LG+I+G4

Supplementary Table 7: Overview of alignments of genes used for phylogenomic reconstruction. Table generated with phylociraptor. This information is also provided in the Supplementary-Data-3 file in EXCEL format. *(continued)*

gene	length	no. of sequences	no. of parsimony informative sites	no. of variable sites	no. of fixed sites	best model
EOG092D14CG	695	79	237	685	10	LG+I+G4
EOG092D1JIT	495	81	270	415	80	LG+I+G4
EOG092D1QPX	672	77	365	645	27	LG+F+I+G4
EOG092D3223	520	80	212	444	76	JTT+I+G4
EOG092D2P76	347	81	219	278	69	LG+I+G4
EOG092D0XB4	868	83	466	772	96	LG+F+I+G4
EOG092D24SJ	451	78	274	367	84	LG+I+G4
EOG092D4SCY	198	78	106	173	25	LG+I+G4
EOG092D28M7	402	83	215	350	52	LG+F+I+G4
EOG092D0WLP	1160	81	430	935	225	JTT+F+I+G4
EOG092D30H2	481	77	258	358	123	LG+I+G4
EOG092D19HX	703	82	355	638	65	LG+F+I+G4
EOG092D2HRE	443	82	121	389	54	LG+F+I+G4
EOG092D0E6G	766	81	281	488	278	LG+I+G4
EOG092D1XFN	419	79	141	385	34	WAG+F+I+G4
EOG092D0IU7	728	81	362	463	265	LG+I+G4
EOG092D2TPW	369	82	170	301	68	LG+I+G4
EOG092D27RI	440	81	172	306	134	JTT+I+G4
EOG092D4E63	150	76	23	46	104	LG+I+G4
EOG092D4A46	547	81	56	517	30	JTT+F+I+G4
EOG092D3OHP	296	81	188	281	15	LG+I+G4
EOG092D0OAE	1131	81	558	1058	73	JTT+I+G4
EOG092D13HB	614	83	274	400	214	LG+I+G4
EOG092D1YWG	464	82	77	274	190	LG+I+G4
EOG092D3KSF	265	78	163	203	62	LG+I+G4
EOG092D0OOP	692	79	235	314	378	LG+I+G4
EOG092D4F44	197	60	141	184	13	LG+G4
EOG092D0CFT	1030	83	534	729	301	LG+I+G4
EOG092D3014	508	82	224	395	113	LG+F+I+G4
EOG092D2S6W	313	65	222	305	8	LG+I+G4
EOG092D3SP3	310	80	177	256	54	LG+I+G4
EOG092D0T75	657	77	375	515	142	LG+F+I+G4
EOG092D0CWO	1013	82	596	728	285	LG+F+I+G4
EOG092D1J3R	476	82	210	326	150	LG+I+G4
EOG092D4OVK	170	79	85	151	19	JTT+I+G4
EOG092D25EQ	367	82	180	312	55	LG+I+G4
EOG092D2I7R	362	81	205	293	69	LG+I+G4
EOG092D0YQS	722	82	452	607	115	LG+I+G4
EOG092D0431	1687	79	654	1657	30	JTT+F+I+G4
EOG092D2Q2D	626	83	227	585	41	JTT+F+I+G4
EOG092D28LI	379	82	157	235	144	LG+I+G4
EOG092D0XW1	1013	81	496	917	96	JTT+F+I+G4
EOG092D2FXY	287	78	176	276	11	LG+I+G4
EOG092D4GR9	178	82	97	153	25	LG+I+G4
EOG092D37TR	311	79	133	245	66	JTTDCMut+I+G4
EOG092D4383	188	80	63	111	77	LG+I+G4
EOG092D339Q	428	80	184	362	66	WAG+I+G4
EOG092D2HX1	362	79	187	267	95	LG+F+I+G4
EOG092D0E4N	896	80	195	891	5	JTTDCMut+I+G4
EOG092D1LC1	786	77	276	759	27	JTT+F+I+G4
EOG092D37V8	262	79	189	232	30	LG+I+G4
EOG092D07LW	1196	81	525	1167	29	JTT+F+I+G4
EOG092D3HQ5	256	80	164	209	47	LG+I+G4
EOG092D4Q5Q	136	76	43	109	27	LG+I+G4
EOG092D1Q7K	497	80	240	406	91	JTT+I+G4
EOG092D29P8	357	78	164	320	37	LG+I+G4

Supplementary Table 7: Overview of alignments of genes used for phylogenomic reconstruction. Table generated with phylociraptor. This information is also provided in the Supplementary-Data-3 file in EXCEL format. *(continued)*

gene	length	no. of sequences	no. of parsimony informative sites	no. of variable sites	no. of fixed sites	best model
EOG092D2N6K	303	79	198	296	7	LG+G4
EOG092D3JQ0	200	79	109	181	19	LG+I+G4
EOG092D4O5W	103	78	55	80	23	LG+G4
EOG092D1AZP	1043	82	362	918	125	JTT+F+I+G4
EOG092D15YH	678	79	394	630	48	JTTDCMut+I+G4
EOG092D1VPO	467	82	217	327	140	LG+I+G4
EOG092D3Q3O	240	66	102	221	19	LG+I+G4
EOG092D45MK	240	78	121	224	16	JTT+F+I+G4
EOG092D3BOU	477	81	121	458	19	LG+F+I+G4
EOG092D3GX0	305	57	139	292	13	LG+F+I+G4
EOG092D0O5Q	757	83	367	575	182	LG+I+G4
EOG092D23P5	364	83	174	290	74	JTTDCMut+I+G4
EOG092D2TS7	501	81	243	449	52	JTT+I+G4
EOG092D2MZ4	323	83	125	303	20	LG+I+G4
EOG092D01YA	2272	83	900	1888	384	LG+I+G4
EOG092D0GJX	716	76	401	655	61	JTT+I+G4
EOG092D0B03	1230	83	435	1093	137	JTT+I+G4
EOG092D20QX	410	75	198	408	2	JTT+F+I+G4
EOG092D3NFG	288	81	165	281	7	JTT+G4
EOG092D09R6	952	74	593	769	183	LG+I+G4
EOG092D4I2T	152	71	81	140	12	JTT+I+G4
EOG092D10GL	561	82	324	456	105	JTT+I+G4
EOG092D4GCP	325	81	135	304	21	LG+I+G4
EOG092D43HN	188	75	107	148	40	LG+G4
EOG092D0FWO	1048	78	889	1011	37	LG+F+I+G4
EOG092D22W1	648	81	221	512	136	LG+F+I+G4
EOG092D2GZ4	314	73	232	310	4	JTTDCMut+G4
EOG092D3TKU	184	70	127	152	32	LG+I+G4
EOG092D49R5	189	62	132	186	3	LG+F+I+G4
EOG092D2J6Q	591	76	255	556	35	JTTDCMut+I+G4
EOG092D4DNZ	166	78	87	144	22	LG+I+G4
EOG092D37F4	403	77	220	369	34	LG+F+I+G4
EOG092D00VT	2206	82	1048	1685	521	JTT+I+G4
EOG092D0OVH	1101	81	246	914	187	JTT+F+I+G4
EOG092D37ON	315	82	109	199	116	JTT+I+G4
EOG092D054H	1347	82	855	1260	87	JTT+F+I+G4
EOG092D4G9R	173	76	89	148	25	WAG+I+G4
EOG092D2RJ9	412	79	250	370	42	LG+I+G4
EOG092D3VHD	232	81	137	184	48	LG+I+G4
EOG092D0HWZ	831	80	408	662	169	LG+F+I+G4
EOG092D2NO9	314	81	83	154	160	JTT+I+G4
EOG092D0KGE	1199	82	254	970	229	JTT+F+I+G4
EOG092D16B7	585	82	294	525	60	LG+I+G4
EOG092D0SNY	572	76	323	466	106	LG+I+G4
EOG092D46DE	188	79	125	166	22	LG+I+G4
EOG092D4J6A	230	77	104	222	8	WAG+I+G4
EOG092D1HUK	529	81	195	272	257	LG+I+G4
EOG092D4IJQ	122	76	91	109	13	LG+I+G4
EOG092D49MT	211	81	134	183	28	LG+G4
EOG092D1UCZ	471	83	330	423	48	LG+F+I+G4
EOG092D4MX9	142	78	105	132	10	LG+G4
EOG092D2PES	330	83	177	307	23	LG+I+G4
EOG092D3I0C	239	81	136	166	73	LG+I+G4
EOG092D3QR7	299	79	122	215	84	LG+I+G4
EOG092D2YGY	402	82	231	324	78	LG+I+G4
EOG092D1GUU	825	81	396	675	150	JTT+I+G4

Supplementary Table 7: Overview of alignments of genes used for phylogenomic reconstruction. Table generated with phylociraptor. This information is also provided in the Supplementary-Data-3 file in EXCEL format. *(continued)*

gene	length	no. of sequences	no. of parsimony informative sites	no. of variable sites	no. of fixed sites	best model
EOG092D3E5G	256	72	165	212	44	JTTDCMut+I+G4
EOG092D31U4	389	77	93	371	18	LG+F+I+G4
EOG092D2BYU	426	81	187	274	152	LG+I+G4
EOG092D2QY5	847	82	322	749	98	JTT+F+I+G4
EOG092D41CK	303	77	166	258	45	LG+F+I+G4
EOG092D23JK	514	83	290	452	62	LG+I+G4
EOG092D44MC	190	73	141	178	12	LG+G4
EOG092D2HF9	417	82	197	280	137	LG+F+I+G4
EOG092D0A17	1127	82	291	1022	105	JTT+I+G4
EOG092D3XG3	221	79	99	158	63	Dayhoff+I+G4
EOG092D00LL	2345	82	630	1045	1300	LG+I+G4
EOG092D0H4I	1026	82	341	984	42	JTT+F+I+G4
EOG092D49G9	211	82	110	208	3	JTTDCMut+I+G4
EOG092D3OPM	248	77	180	212	36	LG+F+I+G4
EOG092D37JC	292	81	194	275	17	LG+I+G4
EOG092D3ZUC	245	79	151	216	29	LG+I+G4
EOG092D1R2W	794	83	395	742	52	JTTDCMut+I+G4
EOG092D0Y8Q	881	80	474	764	117	JTTDCMut+I+G4
EOG092D041E	1269	71	581	1228	41	LG+F+I+G4
EOG092D11EA	674	83	333	541	133	LG+I+G4
EOG092D4FU5	189	77	103	167	22	WAG+I+G4
EOG092D41S5	485	82	69	388	97	LG+I+G4
EOG092D3GEY	354	80	211	308	46	LG+I+G4
EOG092D26ZW	414	82	178	301	113	JTTDCMut+I+G4
EOG092D2U1L	335	81	206	294	41	LG+I+G4
EOG092D1UL3	669	83	287	591	78	JTT+F+I+G4
EOG092D0643	1358	82	730	1320	38	LG+F+I+G4
EOG092D1TC1	589	81	169	551	38	WAG+I+G4
EOG092D3S4W	395	83	141	373	22	WAG+I+G4
EOG092D06GR	1029	81	575	917	112	LG+I+G4
EOG092D2ZDK	324	50	110	226	98	LG+G4
EOG092D225O	493	82	308	407	86	JTTDCMut+I+G4
EOG092D09PM	1142	83	390	1069	73	JTT+F+I+G4
EOG092D467U	347	65	206	345	2	LG+I+G4
EOG092D4EPH	190	81	97	157	33	LG+I+G4
EOG092D4O6D	119	80	92	110	9	JTT+G4
EOG092D05LP	1249	81	896	1072	177	JTTDCMut+F+I+G4
EOG092D3G4V	193	80	85	182	11	LG+I+G4
EOG092D0LL2	813	81	410	684	129	LG+F+I+G4
EOG092D19T7	759	78	437	636	123	LG+I+G4
EOG092D3K9O	269	80	110	193	76	LG+I+G4
EOG092D27I5	386	81	182	314	72	LG+I+G4
EOG092D1Q94	669	80	430	612	57	JTT+I+G4
EOG092D2OLS	728	82	126	696	32	JTT+I+G4
EOG092D4BSZ	175	78	78	116	59	LG+I+G4
EOG092D38RE	403	82	233	374	29	LG+I+G4
EOG092D329B	532	80	355	501	31	LG+F+I+G4
EOG092D25JG	445	79	256	383	62	LG+I+G4
EOG092D116A	789	77	485	772	17	JTT+I+G4
EOG092D4HT4	209	77	104	199	10	LG+F+I+G4
EOG092D36PK	333	70	143	277	56	LG+F+I+G4
EOG092D47GJ	194	81	113	162	32	LG+I+G4
EOG092D20Y4	523	81	304	433	90	LG+I+G4
EOG092D2457	1095	81	191	1067	28	JTT+F+I+G4
EOG092D034L	2042	82	456	2015	27	JTT+F+I+G4
EOG092D12AA	570	82	374	479	91	LG+I+G4

Supplementary Table 7: Overview of alignments of genes used for phylogenomic reconstruction. Table generated with phylociraptor. This information is also provided in the Supplementary-Data-3 file in EXCEL format. *(continued)*

gene	length	no. of sequences	no. of parsimony informative sites	no. of variable sites	no. of fixed sites	best model
EOG092D1OL9	808	79	363	598	210	JTTDCMut+I+G4
EOG092D08FO	1178	80	652	890	288	JTT+F+I+G4
EOG092D1ZGE	763	83	295	716	47	JTT+F+I+G4
EOG092D3UPV	210	72	49	75	135	JTT+I+G4
EOG092D4J2E	229	76	87	211	18	JTT+F+I+G4
EOG092D2RJR	759	82	288	616	143	JTT+F+I+G4
EOG092D09TB	974	81	512	772	202	LG+I+G4
EOG092D47XX	181	83	101	160	21	LG+I+G4
EOG092D3VPV	552	81	211	501	51	JTT+F+I+G4
EOG092D0ZNF	929	81	413	790	139	JTT+I+G4
EOG092D4X9E	96	80	38	72	24	LG+G4
EOG092D2F5H	423	78	218	373	50	LG+I+G4
EOG092D2HI6	562	80	191	510	52	LG+I+G4
EOG092D48MS	302	79	194	289	13	LG+I+G4
EOG092D1KWA	583	83	360	491	92	LG+F+I+G4
EOG092D2YOQ	450	78	262	408	42	LG+I+G4
EOG092D4CCD	147	78	47	101	46	LG+G4
EOG092D3YZ8	150	39	104	133	17	LG+I+G4
EOG092D42VQ	229	54	136	192	37	LG+I+G4
EOG092D14XV	895	80	385	873	22	JTT+F+I+G4
EOG092D247K	398	76	231	332	66	LG+I+G4
EOG092D23EJ	473	82	242	391	82	LG+F+I+G4
EOG092D1Q2Q	289	82	216	276	13	LG+I+G4
EOG092D0NUZ	683	82	305	495	188	LG+I+G4
EOG092D13QH	639	79	363	468	171	LG+I+G4
EOG092D4557	175	80	48	76	99	LG+I+G4
EOG092D0DYZ	831	73	479	754	77	JTT+I+G4
EOG092D1GCX	681	80	345	555	126	JTTDCMut+I+G4
EOG092D0LJL	951	82	549	898	53	LG+F+I+G4
EOG092D39BT	192	79	114	161	31	JTT+I+G4
EOG092D24WU	724	72	406	653	71	LG+I+G4
EOG092D0JFU	901	67	123	899	2	JTT+F+I+G4
EOG092D2B2Y	418	79	267	340	78	LG+I+G4
EOG092D3FNY	551	82	217	519	32	JTT+F+I+G4
EOG092D261T	902	82	572	768	134	LG+I+G4
EOG092D3OQG	309	75	166	226	83	JTT+I+G4
EOG092D13AE	560	81	405	504	56	LG+I+G4
EOG092D0H69	938	75	305	927	11	LG+F+I+G4
EOG092D1EUT	200	81	94	136	64	LG+I+G4
EOG092D4MKM	182	80	79	138	44	LG+G4
EOG092D03HZ	1765	77	1290	1677	88	LG+F+I+G4
EOG092D4EV2	292	81	122	286	6	JTTDCMut+F+I+G4
EOG092D1QLG	748	82	257	637	111	JTT+F+I+G4
EOG092D0EGX	945	82	360	663	282	JTT+F+I+G4
EOG092D4CHM	673	82	257	663	10	JTTDCMut+I+G4
EOG092D2TLM	317	68	225	277	40	LG+I+G4
EOG092D2M1W	386	78	202	332	54	JTT+I+G4
EOG092D3TL4	205	81	63	116	89	LG+I+G4
EOG092D03AT	1681	82	235	1639	42	JTT+F+I+G4
EOG092D09SW	981	80	618	930	51	JTT+F+I+G4
EOG092D1ZEW	545	79	316	487	58	LG+F+I+G4
EOG092D0JGD	956	83	498	895	61	JTT+F+I+G4
EOG092D4APA	116	33	91	114	2	LG+G4
EOG092D1NW2	516	66	282	452	64	LG+F+I+G4
EOG092D4ISY	124	51	19	71	53	Dayhoff+G4
EOG092D2XUX	272	79	180	243	29	LG+F+I+G4

Supplementary Table 7: Overview of alignments of genes used for phylogenomic reconstruction. Table generated with phylociraptor. This information is also provided in the Supplementary-Data-3 file in EXCEL format. *(continued)*

gene	length	no. of sequences	no. of parsimony informative sites	no. of variable sites	no. of fixed sites	best model
EOG092D2806	498	81	208	449	49	JTT+F+I+G4
EOG092D4V60	68	78	44	50	18	LG+I+G4
EOG092D105K	655	83	292	554	101	JTTDCMut+I+G4
EOG092D3Y1Q	235	77	165	214	21	LG+I+G4
EOG092D3UQ8	257	78	166	199	58	LG+I+G4
EOG092D0CUY	1434	64	314	1429	5	LG+F+I+G4
EOG092D1UWS	710	83	273	635	75	JTT+F+I+G4
EOG092D0UKN	950	81	577	850	100	LG+I+G4
EOG092D0S50	1111	82	374	1046	65	JTT+F+I+G4
EOG092D4B4O	413	80	188	365	48	LG+I+G4
EOG092D2ERC	589	82	213	580	9	LG+I+G4
EOG092D3JQI	394	82	202	331	63	JTT+I+G4
EOG092D0AE9	1390	82	697	1138	252	JTT+F+I+G4
EOG092D1M2S	553	83	394	541	12	LG+F+I+G4
EOG092D1CM1	627	82	315	617	10	JTT+F+I+G4
EOG092D4HJN	114	80	69	99	15	LG+I+G4
EOG092D0FXM	640	69	247	611	29	LG+F+I+G4
EOG092D430G	336	80	88	307	29	JTT+F+I+G4
EOG092D1KRH	665	83	83	493	172	JTT+I+G4
EOG092D0S6R	788	80	415	698	90	LG+I+G4
EOG092D3G7O	559	76	309	488	71	LG+F+I+G4
EOG092D4J1H	138	80	46	78	60	LG+I+G4
EOG092D04KS	1711	79	682	1528	183	JTT+F+I+G4
EOG092D4S9W	133	82	90	116	17	LG+I+G4
EOG092D4PXN	120	82	74	98	22	LG+I+G4
EOG092D3DNZ	336	81	163	255	81	JTTDCMut+I+G4
EOG092D4E28	165	73	98	132	33	LG+I+G4
EOG092D2YAG	425	77	289	406	19	LG+I+G4
EOG092D31CV	283	82	127	231	52	LG+I+G4
EOG092D4A4K	143	78	78	105	38	LG+G4
EOG092D3F6H	256	78	135	196	60	LG+I+G4
EOG092D18SO	505	79	334	473	32	LG+F+I+G4
EOG092D0T8J	667	82	342	610	57	JTT+I+G4
EOG092D3DMS	855	81	234	802	53	JTT+F+I+G4
EOG092D3R6J	230	81	102	152	78	LG+I+G4
EOG092D2MK3	593	78	388	560	33	JTT+F+I+G4
EOG092D33VX	307	81	119	298	9	LG+F+I+G4
EOG092D3R78	311	79	108	302	9	WAG+I+G4
EOG092D3AVM	608	80	129	536	72	JTT+F+I+G4
EOG092D2PJJ	443	79	241	335	108	LG+I+G4
EOG092D3J6B	245	81	67	141	104	LG+I+G4
EOG092D0320	1381	76	299	1330	51	JTT+I+G4
EOG092D3DN6	298	75	93	172	126	LG+I+G4
EOG092D0KG3	1293	71	434	1266	27	JTT+F+I+G4
EOG092D3M3Y	445	82	50	438	7	JTT+I+G4
EOG092D1SLT	422	56	286	374	48	LG+I+G4
EOG092D01WX	1865	81	472	1793	72	JTT+F+I+G4
EOG092D2KS9	406	78	280	361	45	LG+I+G4
EOG092D1YHF	467	83	201	364	103	LG+I+G4
EOG092D0H0I	1103	73	572	1055	48	JTT+F+I+G4
EOG092D4IME	223	81	122	196	27	LG+I+G4
EOG092D4MUW	116	73	67	103	13	LG+I+G4
EOG092D25WL	346	82	213	276	70	LG+I+G4
EOG092D4JQM	300	62	60	287	13	JTT+I+G4
EOG092D4AOT	276	80	207	261	15	LG+F+I+G4
EOG092D3CSS	296	83	178	257	39	LG+I+G4

Supplementary Table 7: Overview of alignments of genes used for phylogenomic reconstruction. Table generated with phylociraptor. This information is also provided in the Supplementary-Data-3 file in EXCEL format. *(continued)*

gene	length	no. of sequences	no. of parsimony informative sites	no. of variable sites	no. of fixed sites	best model
EOG092D0S8J	759	66	410	705	54	LG+F+I+G4
EOG092D2Q89	419	79	94	409	10	LG+I+G4
EOG092D0WLN	737	80	277	690	47	LG+F+I+G4
EOG092D00R3	2203	82	1010	1452	751	LG+I+G4
EOG092D3YU3	453	80	166	418	35	JTT+I+G4
EOG092D1QEF	708	80	357	618	90	LG+I+G4
EOG092D3NPH	233	81	139	190	43	LG+I+G4
EOG092D4J7I	116	83	71	92	24	JTT+I+G4
EOG092D3K72	246	78	145	175	71	LG+F+I+G4
EOG092D28HS	474	80	189	287	187	WAG+I+G4
EOG092D3VON	242	80	107	194	48	LG+I+G4
EOG092D1A1J	553	79	343	473	80	LG+I+G4
EOG092D44FK	1236	80	127	1222	14	JTTDCMut+F+I+G4
EOG092D07ZP	996	81	569	928	68	LG+F+I+G4
EOG092D26IC	349	76	186	321	28	LG+I+G4
EOG092D3TNE	214	75	153	197	17	LG+I+G4
EOG092D2OL1	361	82	225	301	60	LG+I+G4
EOG092D4HAV	183	81	98	124	59	LG+I+G4
EOG092D4WYA	128	72	42	122	6	JTT+I+G4
EOG092D3KII	262	79	129	198	64	LG+I+G4
EOG092D23UZ	628	82	154	462	166	JTT+I+G4
EOG092D0FGS	940	80	497	766	174	LG+F+I+G4
EOG092D2WRH	291	78	239	273	18	LG+I+G4
EOG092D2COI	1184	81	785	1127	57	LG+I+G4
EOG092D2O8B	1026	81	283	954	72	JTT+F+I+G4
EOG092D46HE	279	78	127	259	20	WAG+G4
EOG092D4OAJ	154	80	72	120	34	WAG+I+G4
EOG092D272T	506	82	169	470	36	LG+I+G4
EOG092D49WK	319	77	188	276	43	LG+F+I+G4
EOG092D11XU	652	73	356	549	103	LG+I+G4
EOG092D3PF3	547	81	231	505	42	LG+I+G4
EOG092D344A	316	83	171	267	49	LG+I+G4
EOG092D0BMY	936	81	506	687	249	LG+I+G4
EOG092D0UJ2	593	77	280	576	17	JTT+I+G4
EOG092D29KI	449	78	168	219	230	LG+I+G4
EOG092D3N3B	279	77	138	229	50	Dayhoff+I+G4
EOG092D412G	170	80	90	134	36	LG+I+G4
EOG092D47Y3	187	69	77	145	42	LG+I+G4
EOG092D31X5	392	60	239	315	77	LG+I+G4
EOG092D04HM	1476	82	527	1408	68	JTT+F+I+G4
EOG092D0GXN	909	83	407	742	167	LG+F+I+G4
EOG092D3F1G	547	82	210	518	29	JTT+I+G4
EOG092D4D9K	409	74	224	380	29	LG+I+G4
EOG092D49VZ	165	74	74	94	71	LG+I+G4
EOG092D0KBR	824	80	454	679	145	LG+F+I+G4
EOG092D3HUP	243	80	125	209	34	LG+I+G4
EOG092D3K5F	312	79	112	179	133	LG+I+G4
EOG092D3KV2	331	81	173	242	89	LG+F+I+G4
EOG092D0J9G	913	80	334	540	373	LG+I+G4
EOG092D29QC	548	78	271	535	13	LG+F+I+G4
EOG092D1YZO	416	82	151	281	135	JTT+I+G4
EOG092D3LCX	231	79	138	185	46	LG+I+G4
EOG092D1GVT	557	81	159	259	298	LG+F+I+G4
EOG092D0072	4255	81	2225	3204	1051	JTT+F+I+G4
EOG092D4JRM	188	78	72	174	14	LG+F+I+G4
EOG092D07D8	1708	81	237	1598	110	JTT+I+G4

Supplementary Table 7: Overview of alignments of genes used for phylogenomic reconstruction. Table generated with phylociraptor. This information is also provided in the Supplementary-Data-3 file in EXCEL format. *(continued)*

gene	length	no. of sequences	no. of parsimony informative sites	no. of variable sites	no. of fixed sites	best model
EOG092D3P54	237	76	133	204	33	JTT+F+I+G4
EOG092D1MNK	486	82	174	307	179	LG+I+G4
EOG092D3QV4	219	80	48	120	99	LG+I+G4
EOG092D2CAF	355	82	131	274	81	LG+I+G4
EOG092D4ASP	226	60	139	203	23	LG+G4
EOG092D3H8K	297	82	134	199	98	LG+I+G4
EOG092D4I8P	152	79	81	113	39	LG+I+G4
EOG092D2LX2	346	79	139	220	126	LG+I+G4
EOG092D4S3D	169	60	123	153	16	LG+G4
EOG092D3SP4	339	76	203	268	71	LG+F+I+G4
EOG092D3E1M	605	80	204	557	48	JTTDCMut+I+G4
EOG092D1S33	495	81	237	345	150	LG+I+G4
EOG092D0K9C	857	78	284	803	54	JTT+F+I+G4
EOG092D1G2Z	564	80	108	421	143	LG+F+I+G4
EOG092D4CIZ	255	78	143	239	16	LG+F+I+G4
EOG092D164Q	539	75	306	464	75	LG+F+I+G4
EOG092D0LVZ	844	82	561	758	86	LG+F+I+G4
EOG092D33FU	424	79	253	372	52	LG+I+G4
EOG092D0BYJ	1048	82	486	670	378	LG+F+I+G4
EOG092D0T3V	593	77	278	537	56	LG+F+I+G4
EOG092D2XS7	465	83	327	424	41	LG+I+G4
EOG092D0OXK	838	80	459	636	202	LG+I+G4
EOG092D1CKF	876	81	376	745	131	LG+I+G4
EOG092D1N0P	744	81	484	705	39	LG+F+I+G4
EOG092D07JG	1086	75	494	758	328	LG+I+G4
EOG092D4H8I	133	64	80	116	17	LG+G4
EOG092D1YID	478	83	193	350	128	LG+I+G4
EOG092D1ZEJ	442	81	131	356	86	LG+I+G4
EOG092D056B	1222	82	349	1193	29	WAG+F+I+G4
EOG092D0HGD	1189	81	209	1163	26	JTT+F+I+G4
EOG092D4CW1	200	51	59	155	45	JTT+I+G4
EOG092D065A	1269	72	705	1198	71	LG+I+G4
EOG092D2925	685	80	303	644	41	JTT+F+I+G4
EOG092D406X	201	82	103	159	42	JTT+I+G4
EOG092D0QAH	929	81	564	851	78	LG+I+G4
EOG092D1O7A	495	82	149	279	216	LG+I+G4
EOG092D03RY	1318	79	503	1302	16	JTT+F+I+G4
EOG092D0LJK	853	79	369	624	229	LG+F+I+G4
EOG092D2BJH	524	82	266	365	159	LG+I+G4
EOG092D3K5U	305	78	171	294	11	LG+I+G4
EOG092D3MS3	213	81	157	202	11	LG+I+G4
EOG092D03PE	1494	76	886	1441	53	JTT+F+I+G4
EOG092D4MP9	123	81	55	88	35	LG+G4
EOG092D0ILO	900	81	326	878	22	JTT+F+I+G4
EOG092D3X2Q	228	75	162	200	28	JTT+I+G4
EOG092D2G7T	356	77	198	241	115	LG+I+G4
EOG092D4GG0	133	74	106	129	4	LG+I+G4
EOG092D32SI	301	77	155	268	33	LG+F+I+G4
EOG092D4J3B	105	73	67	84	21	LG+I+G4
EOG092D1H8I	518	78	209	457	61	LG+F+I+G4
EOG092D1VKT	534	81	264	449	85	LG+I+G4
EOG092D3PPI	252	75	73	205	47	VT+I+G4
EOG092D4QSJ	117	74	70	99	18	LG+I+G4
EOG092D1MQ6	571	82	186	256	315	LG+I+G4
EOG092D0P0O	1013	75	305	1001	12	JTT+F+I+G4
EOG092D2Z5Z	298	80	144	205	93	LG+I+G4

Supplementary Table 7: Overview of alignments of genes used for phylogenomic reconstruction. Table generated with phylociraptor. This information is also provided in the Supplementary-Data-3 file in EXCEL format. *(continued)*

gene	length	no. of sequences	no. of parsimony informative sites	no. of variable sites	no. of fixed sites	best model
EOG092D2FDT	404	74	260	390	14	LG+F+I+G4
EOG092D2QYY	360	76	189	342	18	LG+F+I+G4
EOG092D23WW	424	81	229	372	52	LG+I+G4
EOG092D2O1N	999	80	280	976	23	JTT+F+I+G4
EOG092D3THJ	272	77	141	238	34	LG+I+G4
EOG092D0R9A	774	80	417	732	42	JTT+F+I+G4
EOG092D0ULK	1003	83	373	846	157	JTT+I+G4
EOG092D0905	1276	80	480	1197	79	JTT+F+I+G4
EOG092D3PHH	146	80	84	141	5	LG+I+G4
EOG092D40TG	200	77	87	134	66	LG+I+G4
EOG092D080M	1037	80	570	865	172	LG+I+G4
EOG092D02KN	1828	81	576	1739	89	JTT+I+G4
EOG092D2AZW	479	78	179	435	44	JTTDCMut+I+G4
EOG092D36RY	314	76	146	293	21	LG+G4
EOG092D4241	322	79	124	302	20	LG+I+G4
EOG092D3NKD	322	77	114	300	22	JTTDCMut+F+I+G4
EOG092D3X8S	185	72	116	172	13	WAG+I+G4
EOG092D4X1O	163	81	79	135	28	JTT+F+I+G4
EOG092D0UFO	770	83	412	626	144	WAG+I+G4
EOG092D0W9K	600	81	205	381	219	LG+I+G4
EOG092D0S5N	609	81	302	605	4	LG+I+G4
EOG092D1AOO	308	74	159	217	91	LG+I+G4
EOG092D2VWC	382	81	129	377	5	LG+F+I+G4
EOG092D4145	343	81	139	297	46	LG+F+I+G4
EOG092D43XW	200	82	116	187	13	LG+I+G4
EOG092D13PM	709	82	364	600	109	JTT+F+I+G4
EOG092D45IW	170	78	113	161	9	LG+I+G4
EOG092D3HVD	293	81	182	244	49	LG+I+G4
EOG092D0Q00	1074	79	642	1010	64	LG+I+G4
EOG092D19EB	1730	81	391	1620	110	JTT+F+I+G4
EOG092D3HBU	511	76	272	506	5	LG+F+I+G4
EOG092D3WPA	291	79	141	235	56	JTT+I+G4
EOG092D2LER	407	78	152	329	78	LG+F+I+G4
EOG092D4PH2	197	60	129	172	25	JTT+I+G4
EOG092D0ANR	1776	83	801	1662	114	JTT+F+I+G4
EOG092D1WA5	487	81	294	407	80	LG+I+G4
EOG092D28YM	640	83	142	598	42	JTT+F+I+G4
EOG092D0ZVY	644	77	254	518	126	JTT+F+I+G4
EOG092D0DNM	1513	83	508	1214	299	JTT+F+I+G4
EOG092D1RSS	312	83	154	285	27	LG+I+G4
EOG092D1AOA	535	71	258	449	86	LG+I+G4
EOG092D32P7	263	78	124	209	54	LG+I+G4
EOG092D4M0K	128	80	49	82	46	Dayhoff+I+G4
EOG092D05Q0	1205	83	657	1132	73	JTT+I+G4
EOG092D3XA6	351	82	173	317	34	LG+F+I+G4
EOG092D1884	836	82	178	700	136	JTT+F+I+G4
EOG092D3IJD	399	83	237	361	38	JTT+I+G4
EOG092D05X9	1356	82	890	1198	158	LG+I+G4
EOG092D0NP5	1193	82	579	1162	31	JTT+I+G4
EOG092D048Q	1877	80	479	1842	35	JTT+F+I+G4
EOG092D3Z6V	511	77	267	418	93	JTT+F+I+G4
EOG092D0Q23	796	76	273	521	275	LG+I+G4
EOG092D3WSA	297	83	105	241	56	JTT+F+I+G4
EOG092D40PM	161	77	79	143	18	LG+I+G4
EOG092D2RFR	365	76	160	215	150	LG+I+G4
EOG092D360D	421	81	225	398	23	LG+I+G4

Supplementary Table 7: Overview of alignments of genes used for phylogenomic reconstruction. Table generated with phylociraptor. This information is also provided in the Supplementary-Data-3 file in EXCEL format. *(continued)*

gene	length	no. of sequences	no. of parsimony informative sites	no. of variable sites	no. of fixed sites	best model
EOG092D4UA6	75	74	40	68	7	LG+G4
EOG092D2VYQ	309	62	195	291	18	LG+I+G4
EOG092D3LGX	242	41	170	226	16	LG+I+G4
EOG092D485T	268	80	101	227	41	JTTDCMut+F+I+G4
EOG092D3NUF	306	80	92	252	54	Dayhoff+I+G4
EOG092D4OIQ	132	80	70	98	34	LG+I+G4
EOG092D4TT8	101	80	49	83	18	LG+I+G4
EOG092D01IY	1782	81	919	1511	271	JTT+I+G4
EOG092D0FHW	861	82	418	755	106	LG+F+I+G4
EOG092D0UNZ	555	78	333	526	29	LG+I+G4
EOG092D4C5U	386	82	111	361	25	JTT+F+I+G4
EOG092D16QM	535	77	307	519	16	LG+I+G4
EOG092D0O61	1080	80	425	756	324	JTT+F+I+G4
EOG092D2W3S	519	82	195	463	56	JTT+I+G4
EOG092D3LD3	364	83	167	328	36	LG+F+I+G4
EOG092D4DOH	142	76	102	115	27	LG+G4
EOG092D465Q	219	79	146	199	20	LG+F+I+G4
EOG092D0EUY	954	82	638	894	60	JTT+F+I+G4
EOG092D02DP	1584	82	918	1465	119	JTT+F+I+G4
EOG092D1V62	681	83	122	669	12	JTT+I+G4
EOG092D4POO	205	82	70	193	12	JTT+I+G4
EOG092D47QN	319	79	151	275	44	JTTDCMut+I+G4
EOG092D02YC	1628	80	549	1482	146	JTT+I+G4
EOG092D1OV6	521	73	145	361	160	JTT+I+G4
EOG092D310Y	376	81	214	339	37	LG+I+G4
EOG092D1US9	608	81	223	557	51	LG+F+I+G4
EOG092D2A51	576	81	265	563	13	LG+I+G4
EOG092D3Y3L	216	82	58	97	119	WAG+I+G4
EOG092D37Y7	332	81	92	175	157	JTT+I+G4
EOG092D3RNW	576	83	244	503	73	JTT+I+G4
EOG092D4M8A	137	79	24	63	74	LG+I+G4
EOG092D4QXS	116	79	53	100	16	WAG+I+G4
EOG092D3U3F	235	81	137	189	46	LG+I+G4
EOG092D17TE	546	82	250	346	200	LG+I+G4
EOG092D2QGC	347	82	256	324	23	LG+F+G4
EOG092D0GSV	818	79	481	731	87	LG+I+G4
EOG092D0MSY	850	79	547	723	127	LG+I+G4
EOG092D1AQR	791	82	297	679	112	JTT+I+G4
EOG092D4TKN	130	80	73	110	20	LG+I+G4
EOG092D12VX	775	82	302	742	33	LG+F+I+G4
EOG092D0NR0	878	80	336	640	238	LG+F+I+G4
EOG092D2DAM	374	82	192	321	53	LG+I+G4
EOG092D1Q5G	865	81	175	628	237	JTT+F+I+G4
EOG092D30CB	327	67	170	316	11	LG+I+G4
EOG092D0YYX	1133	82	434	968	165	JTT+F+I+G4
EOG092D4RXG	98	81	68	86	12	LG+G4
EOG092D3W5S	226	79	90	216	10	LG+I+G4
EOG092D3JLV	251	82	110	171	80	LG+I+G4
EOG092D22H1	497	82	276	437	60	LG+I+G4
EOG092D2KKF	1514	80	208	1434	80	JTT+I+G4
EOG092D0124	2350	82	1003	2274	76	JTT+F+I+G4
EOG092D4A7L	257	67	128	249	8	WAG+F+I+G4
EOG092D1CLY	634	82	212	485	149	LG+I+G4
EOG092D1ODU	465	77	249	434	31	LG+I+G4
EOG092D2KNO	378	81	149	238	140	LG+I+G4
EOG092D0V4E	659	80	367	525	134	LG+F+I+G4

Supplementary Table 7: Overview of alignments of genes used for phylogenomic reconstruction. Table generated with phylociraptor. This information is also provided in the Supplementary-Data-3 file in EXCEL format. *(continued)*

gene	length	no. of sequences	no. of parsimony informative sites	no. of variable sites	no. of fixed sites	best model
EOG092D2D7L	402	79	154	247	155	LG+I+G4
EOG092D3ETT	370	82	178	306	64	LG+I+G4
EOG092D3T9X	948	81	329	909	39	JTT+I+G4
EOG092D2570	649	81	324	571	78	JTT+I+G4
EOG092D091W	974	83	597	859	115	LG+I+G4
EOG092D3RDW	655	83	74	574	81	JTT+F+I+G4
EOG092D4GXA	152	79	42	71	81	LG+G4
EOG092D3WBP	348	80	241	336	12	LG+I+G4
EOG092D1XYV	454	82	180	355	99	LG+I+G4
EOG092D2MDL	532	80	129	415	117	JTT+F+I+G4
EOG092D338V	401	79	213	340	61	JTT+I+G4
EOG092D3YUS	203	37	85	129	74	LG+I+G4
EOG092D0IC1	786	79	484	710	76	JTT+I+G4
EOG092D1EM9	473	81	244	332	141	LG+I+G4
EOG092D1I4F	535	82	273	461	74	LG+G4
EOG092D4CM0	209	77	120	149	60	Dayhoff+I+G4
EOG092D31FN	268	79	166	244	24	LG+F+I+G4
EOG092D0KAC	1060	81	376	1052	8	JTT+F+I+G4
EOG092D30F8	282	83	107	163	119	LG+I+G4
EOG092D4BMV	173	82	58	127	46	JTT+I+G4
EOG092D3SHS	267	74	119	202	65	LG+I+G4
EOG092D0FN1	1107	81	400	1061	46	JTT+F+I+G4
EOG092D3EJG	292	77	146	255	37	LG+I+G4
EOG092D0QOX	968	80	270	959	9	LG+F+I+G4
EOG092D2633	445	81	299	368	77	LG+I+G4
EOG092D31Z3	394	79	160	376	18	VT+I+G4
EOG092D3CY0	387	65	172	351	36	LG+F+I+G4
EOG092D3LN2	271	78	198	254	17	LG+I+G4
EOG092D2XBR	514	79	317	453	61	LG+F+I+G4
EOG092D2Q04	371	78	147	255	116	LG+I+G4
EOG092D1SCZ	589	80	273	543	46	JTTDCMut+I+G4
EOG092D06QZ	1206	82	598	1021	185	LG+F+I+G4
EOG092D2APH	382	77	283	359	23	LG+I+G4
EOG092D1HB6	571	83	271	458	113	LG+I+G4
EOG092D1J6A	811	82	146	686	125	JTT+F+I+G4
EOG092D0ZEU	1165	82	472	1030	135	JTT+F+I+G4
EOG092D3039	936	82	358	826	110	JTT+F+I+G4
EOG092D2LGS	411	83	154	393	18	JTT+F+I+G4
EOG092D29J0	721	81	323	620	101	JTTDCMut+I+G4
EOG092D2GP9	489	80	279	437	52	LG+I+G4
EOG092D2O0K	481	80	174	464	17	LG+F+I+G4
EOG092D2OL5	404	78	238	384	20	LG+I+G4
EOG092D49PH	357	80	113	343	14	LG+I+G4
EOG092D22TL	428	82	189	279	149	LG+I+G4
EOG092D3MM0	542	82	197	474	68	JTT+I+G4
EOG092D45EU	248	81	119	237	11	JTTDCMut+I+G4
EOG092D3CMX	743	75	309	733	10	JTT+F+I+G4
EOG092D4824	233	32	112	185	48	JTTDCMut+I+G4
EOG092D0433	2462	82	984	2432	30	JTT+F+I+G4
EOG092D3VPE	271	80	197	247	24	JTT+I+G4
EOG092D13K4	1270	83	692	1196	74	JTT+F+I+G4
EOG092D3Y0X	347	82	250	318	29	LG+F+I+G4
EOG092D38IH	307	79	138	205	102	LG+I+G4
EOG092D08HR	1855	81	747	1674	181	JTT+F+I+G4
EOG092D3JAM	248	77	122	201	47	LG+F+I+G4
EOG092D3DOE	393	83	208	364	29	LG+I+G4

Supplementary Table 7: Overview of alignments of genes used for phylogenomic reconstruction. Table generated with phylociraptor. This information is also provided in the Supplementary-Data-3 file in EXCEL format. *(continued)*

gene	length	no. of sequences	no. of parsimony informative sites	no. of variable sites	no. of fixed sites	best model
EOG092D3WW4	204	80	64	129	75	LG+I+G4
EOG092D28PE	396	76	126	385	11	LG+F+I+G4
EOG092D2UJS	446	79	267	407	39	LG+F+I+G4
EOG092D4M5U	172	80	79	151	21	LG+I+G4
EOG092D23RQ	361	81	202	295	66	LG+I+G4
EOG092D4KK3	163	83	99	149	14	LG+F+I+G4
EOG092D2KHQ	409	82	203	378	31	JTT+I+G4
EOG092D3X7S	358	81	125	341	17	VT+I+G4
EOG092D4LDU	172	75	42	87	85	LG+I+G4
EOG092D339O	425	81	163	396	29	LG+F+I+G4
EOG092D2G09	566	83	249	456	110	JTTDCMut+I+G4
EOG092D1A87	565	83	366	492	73	LG+I+G4
EOG092D2MM2	197	72	108	174	23	LG+F+I+G4
EOG092D3JGK	294	80	193	261	33	LG+F+I+G4
EOG092D00HH	2892	82	1089	2600	292	LG+F+I+G4
EOG092D1BU7	587	74	328	521	66	LG+I+G4
EOG092D0KTI	965	82	480	891	74	LG+I+G4
EOG092D1EK2	462	82	238	395	67	LG+I+G4
EOG092D2GVZ	636	78	260	607	29	JTTDCMut+I+G4
EOG092D33Q2	299	79	135	175	124	LG+I+G4
EOG092D09N2	982	82	361	629	353	LG+I+G4
EOG092D3ODN	299	55	187	280	19	JTTDCMut+I+G4
EOG092D3A58	378	81	185	326	52	LG+F+I+G4
EOG092D0EMK	917	82	408	859	58	JTT+I+G4
EOG092D2AME	521	81	197	431	90	LG+I+G4
EOG092D3YIQ	321	79	38	172	149	JTT+F+I+G4
EOG092D0PER	1124	80	452	999	125	JTTDCMut+F+I+G4
EOG092D0DVR	1017	79	577	845	172	LG+I+G4
EOG092D1N80	528	81	227	434	94	LG+F+I+G4
EOG092D3MIU	260	79	151	186	74	LG+I+G4
EOG092D1I8J	601	83	183	573	28	JTT+I+G4
EOG092D4CHT	178	70	108	167	11	LG+G4
EOG092D3VJX	247	77	165	218	29	LG+I+G4
EOG092D2HJ9	295	81	183	259	36	LG+F+I+G4
EOG092D0PZY	656	76	438	536	120	LG+I+G4
EOG092D48Y7	130	71	90	126	4	LG+G4
EOG092D2ZKG	214	74	145	203	11	JTT+I+G4
EOG092D4HHP	135	42	97	129	6	LG+F+G4
EOG092D1XPE	333	64	141	308	25	LG+I+G4
EOG092D3FYF	277	79	136	248	29	LG+I+G4
EOG092D0J2Q	941	80	272	878	63	JTT+F+I+G4
EOG092D0AVU	898	82	369	821	77	JTT+I+G4
EOG092D4D32	252	72	120	250	2	LG+I+G4
EOG092D4HWC	123	75	83	105	18	LG+F+I+G4
EOG092D27O3	464	82	300	428	36	LG+I+G4
EOG092D2LO5	351	78	258	320	31	LG+I+G4
EOG092D3N04	253	81	129	208	45	JTT+I+G4
EOG092D26WR	404	74	287	388	16	LG+F+I+G4
EOG092D3R6Y	230	75	153	210	20	LG+I+G4
EOG092D2SJQ	585	81	29	486	99	JTT+F+I+G4
EOG092D1GZC	525	83	281	391	134	LG+I+G4
EOG092D24W9	632	59	346	625	7	LG+F+I+G4
EOG092D005G	4901	80	1238	4650	251	JTT+F+I+G4
EOG092D2VEY	584	80	282	520	64	LG+I+G4
EOG092D4K3B	170	83	60	83	87	LG+G4
EOG092D2PVH	481	82	249	463	18	LG+I+G4

Supplementary Table 7: Overview of alignments of genes used for phylogenomic reconstruction. Table generated with phylociraptor. This information is also provided in the Supplementary-Data-3 file in EXCEL format. *(continued)*

gene	length	no. of sequences	no. of parsimony informative sites	no. of variable sites	no. of fixed sites	best model
EOG092D33G3	430	83	263	391	39	LG+I+G4
EOG092D1OVL	745	83	350	694	51	JTT+I+G4
EOG092D0PHK	1139	75	578	885	254	JTT+I+G4
EOG092D3C2F	276	78	159	212	64	LG+I+G4
EOG092D0CUX	914	83	435	689	225	LG+I+G4
EOG092D4074	205	80	99	178	27	LG+I+G4
EOG092D1AFQ	579	76	332	487	92	JTT+I+G4
EOG092D2WPT	293	68	92	281	12	LG+G4
EOG092D0SK9	828	81	255	753	75	JTT+I+G4
EOG092D34D0	262	82	116	190	72	LG+I+G4
EOG092D12XY	774	77	377	728	46	LG+I+G4
EOG092D0FQ1	784	78	460	665	119	LG+I+G4
EOG092D39KW	725	78	360	688	37	JTT+I+G4
EOG092D1IEY	499	80	122	193	306	LG+I+G4
EOG092D32X3	392	75	200	327	65	LG+I+G4
EOG092D3JKF	249	60	134	200	49	JTTDCMut+G4
EOG092D0454	1995	81	489	1912	83	LG+F+I+G4
EOG092D3VHN	208	81	135	176	32	JTT+I+G4
EOG092D3EJQ	321	75	212	258	63	LG+I+G4
EOG092D2G5H	327	80	140	202	125	JTT+I+G4
EOG092D0XC3	737	80	417	630	107	JTT+I+G4
EOG092D1BAU	620	81	263	489	131	JTT+F+I+G4
EOG092D2N1K	240	81	143	221	19	LG+I+G4
EOG092D068O	1708	81	727	1424	284	LG+F+I+G4
EOG092D4S06	126	78	65	107	19	LG+I+G4
EOG092D1J1B	456	43	251	443	13	LG+I+G4
EOG092D3WUX	350	73	216	343	7	LG+F+G4
EOG092D3ZRA	238	82	118	196	42	WAG+I+G4
EOG092D24YM	390	76	279	376	14	LG+F+I+G4
EOG092D1W7D	653	80	186	568	85	JTT+I+G4
EOG092D2I29	426	80	208	392	34	JTT+I+G4
EOG092D03LV	1572	47	477	1538	34	JTT+F+I+G4
EOG092D4HNO	135	78	76	110	25	WAG+G4
EOG092D1QZ9	475	80	153	233	242	LG+I+G4
EOG092D3LSA	240	82	110	163	77	LG+I+G4
EOG092D4H5J	296	82	62	281	15	JTT+I+G4
EOG092D2BNK	601	83	194	488	113	JTT+F+I+G4
EOG092D0B5M	906	82	600	738	168	LG+I+G4
EOG092D1JY2	614	79	374	534	80	LG+F+I+G4
EOG092D40N7	224	80	136	182	42	LG+I+G4
EOG092D1THK	547	71	228	462	85	LG+F+I+G4
EOG092D2R1Y	323	74	244	280	43	LG+F+I+G4
EOG092D1TFV	574	81	373	513	61	LG+I+G4
EOG092D3H3Y	287	73	155	260	27	LG+I+G4
EOG092D304F	342	82	186	273	69	LG+I+G4
EOG092D0KCS	1659	80	617	1467	192	JTT+I+G4
EOG092D3I9A	466	81	353	419	47	LG+F+I+G4
EOG092D2QPK	357	80	188	246	111	LG+F+I+G4
EOG092D3Z9F	190	79	96	169	21	LG+F+I+G4
EOG092D1CJS	558	78	191	295	263	JTT+I+G4
EOG092D2ASD	441	80	190	275	166	LG+I+G4
EOG092D3NOC	323	82	185	301	22	LG+I+G4
EOG092D0H7X	1016	83	429	964	52	LG+F+I+G4
EOG092D0NJO	823	77	309	740	83	JTT+I+G4
EOG092D0XLN	768	80	453	641	127	LG+F+I+G4
EOG092D3ZKF	215	81	122	173	42	LG+I+G4

Supplementary Table 7: Overview of alignments of genes used for phylogenomic reconstruction. Table generated with phylociraptor. This information is also provided in the Supplementary-Data-3 file in EXCEL format. *(continued)*

gene	length	no. of sequences	no. of parsimony informative sites	no. of variable sites	no. of fixed sites	best model
EOG092D3O42	210	81	144	180	30	JTT+I+G4
EOG092D01MX	1847	83	580	1608	239	JTT+I+G4
EOG092D26V2	471	81	175	458	13	LG+I+G4
EOG092D0WCK	847	81	267	798	49	JTT+I+G4
EOG092D1DJB	663	82	136	610	53	JTT+F+I+G4
EOG092D383R	1301	82	310	1229	72	JTT+I+G4
EOG092D0YTO	891	81	427	792	99	JTT+F+I+G4
EOG092D4MIB	176	80	116	153	23	LG+I+G4
EOG092D1VPG	433	80	137	235	198	LG+I+G4
EOG092D343G	298	80	153	243	55	LG+I+G4
EOG092D0CQQ	542	79	323	488	54	LG+I+G4
EOG092D4N4A	137	81	28	75	62	Dayhoff+I+G4
EOG092D1O8L	439	80	186	298	141	LG+I+G4
EOG092D3QEX	268	77	158	262	6	JTTDCMut+G4
EOG092D36LY	351	80	169	219	132	LG+I+G4
EOG092D0Y1F	623	81	313	525	98	JTTDCMut+I+G4
EOG092D18RK	901	82	292	818	83	JTT+F+I+G4
EOG092D38PJ	370	79	199	336	34	LG+I+G4
EOG092D47ZH	208	79	123	170	38	LG+I+G4
EOG092D3LLM	381	80	122	344	37	JTT+I+G4
EOG092D04RH	1303	78	434	1274	29	LG+F+I+G4
EOG092D29YU	535	82	198	466	69	LG+I+G4
EOG092D0VF1	586	78	308	573	13	LG+F+I+G4
EOG092D1CXA	643	73	366	575	68	LG+I+G4
EOG092D074K	1152	83	553	1044	108	JTT+F+I+G4
EOG092D4RL0	120	81	70	101	19	LG+I+G4
EOG092D4597	168	78	120	158	10	LG+F+I+G4
EOG092D131D	807	76	185	797	10	JTT+F+I+G4
EOG092D2UGI	229	75	131	174	55	LG+I+G4
EOG092D16LD	786	82	312	612	174	LG+I+G4
EOG092D1HN1	472	81	243	423	49	JTT+I+G4
EOG092D3MYV	580	81	100	503	77	JTT+F+I+G4
EOG092D3BM8	253	73	180	239	14	LG+I+G4
EOG092D4A3U	228	80	145	212	16	LG+I+G4
EOG092D383L	498	75	255	485	13	LG+I+G4
EOG092D145A	601	79	194	541	60	JTT+F+I+G4
EOG092D3J2L	331	75	128	318	13	WAG+I+G4
EOG092D2U8Y	395	80	211	348	47	LG+I+G4
EOG092D0O9O	1054	81	297	980	74	JTT+I+G4
EOG092D2F9R	449	81	256	409	40	LG+I+G4
EOG092D1VHG	403	76	271	363	40	LG+F+I+G4
EOG092D2VBX	378	80	166	311	67	JTTDCMut+I+G4
EOG092D22SO	1022	78	231	1014	8	JTT+F+I+G4
EOG092D1BN5	640	78	288	577	63	JTT+I+G4
EOG092D425I	230	74	151	201	29	LG+I+G4
EOG092D3X9I	264	71	137	253	11	LG+I+G4
EOG092D2W25	498	76	222	456	42	LG+F+I+G4
EOG092D06DZ	1216	78	648	1005	211	JTT+I+G4
EOG092D3PY2	312	82	210	284	28	LG+F+G4
EOG092D0BHJ	797	82	317	627	170	LG+I+G4
EOG092D4Q0H	107	81	59	74	33	LG+I+G4
EOG092D4E56	309	81	145	300	9	LG+I+G4
EOG092D0KU4	1077	83	503	1019	58	LG+F+I+G4
EOG092D4P6F	177	81	120	169	8	LG+F+I+G4
EOG092D0VZC	684	72	368	674	10	JTT+I+G4
EOG092D2Y9W	351	73	228	344	7	LG+I+G4

Supplementary Table 7: Overview of alignments of genes used for phylogenomic reconstruction. Table generated with phylociraptor. This information is also provided in the Supplementary-Data-3 file in EXCEL format. *(continued)*

gene	length	no. of sequences	no. of parsimony informative sites	no. of variable sites	no. of fixed sites	best model
EOG092D0XEW	797	81	214	698	99	JTT+F+I+G4
EOG092D0PA5	839	78	430	630	209	JTTDCMut+I+G4
EOG092D1ABF	647	82	338	596	51	LG+I+G4
EOG092D4E8N	159	82	92	125	34	JTT+G4
EOG092D274B	496	83	321	416	80	JTT+I+G4
EOG092D2UFS	431	78	262	358	73	JTTDCMut+I+G4
EOG092D02JB	1604	83	828	1297	307	JTT+I+G4
EOG092D10MG	871	67	545	714	157	LG+I+G4
EOG092D2T3Z	364	80	151	244	120	LG+I+G4
EOG092D2N6I	309	82	177	256	53	WAG+I+G4
EOG092D1CGS	547	75	257	473	74	LG+I+G4
EOG092D0A4I	873	81	348	719	154	JTTDCMut+I+G4
EOG092D0ZAJ	433	61	266	395	38	LG+I+G4
EOG092D37VX	375	80	74	205	170	DCMut+F+I+G4
EOG092D1CVU	1067	79	219	975	92	JTT+F+I+G4
EOG092D2OW6	346	72	200	271	75	LG+I+G4
EOG092D1MLK	477	75	260	425	52	LG+I+G4
EOG092D2OSF	351	72	217	309	42	JTT+I+G4
EOG092D02CG	1414	81	822	1192	222	JTT+F+I+G4
EOG092D0PTK	627	82	329	588	39	JTT+I+G4
EOG092D383C	399	83	155	278	121	JTT+F+I+G4
EOG092D2VZF	405	82	242	372	33	LG+I+G4
EOG092D15UU	637	81	220	348	289	LG+I+G4
EOG092D3I4P	303	80	157	265	38	WAG+I+G4
EOG092D3GR4	1518	81	394	1489	29	JTT+F+I+G4
EOG092D2AWS	504	80	197	472	32	LG+F+I+G4
EOG092D27Y2	370	76	214	306	64	JTT+I+G4
EOG092D2SZZ	415	68	268	396	19	JTT+F+I+G4
EOG092D0ACX	1063	83	381	971	92	LG+F+I+G4
EOG092D4EIS	276	78	65	244	32	JTTDCMut+I+G4
EOG092D3HE2	355	82	126	313	42	JTTDCMut+I+G4
EOG092D1RW7	677	80	354	638	39	JTT+F+I+G4
EOG092D4CXI	310	77	155	300	10	LG+F+I+G4
EOG092D0B9X	1022	82	423	652	370	JTT+I+G4
EOG092D49XW	147	76	66	109	38	JTTDCMut+I+G4
EOG092D33N6	567	80	259	544	23	JTT+I+G4
EOG092D3QYI	295	78	201	271	24	LG+I+G4
EOG092D3F2O	291	78	175	235	56	LG+I+G4
EOG092D2X2E	240	80	55	185	55	LG+I+G4
EOG092D0VTV	617	79	294	473	144	LG+I+G4
EOG092D1VHP	341	77	263	336	5	LG+F+I+G4
EOG092D3ZLJ	522	82	210	469	53	JTT+F+I+G4
EOG092D3JAF	285	72	131	268	17	LG+F+I+G4
EOG092D10OW	646	80	383	626	20	JTT+F+I+G4
EOG092D3EQH	536	81	205	495	41	JTTDCMut+F+I+G4
EOG092D0C9Q	1150	78	435	981	169	JTT+I+G4
EOG092D0GP7	752	81	410	505	247	LG+I+G4
EOG092D19EQ	535	78	165	229	306	LG+I+G4
EOG092D0XJC	640	82	260	634	6	JTT+F+I+G4
EOG092D2MHF	576	82	332	551	25	JTT+I+G4
EOG092D0KYR	1499	80	732	1272	227	LG+I+G4
EOG092D29XW	387	78	123	269	118	LG+G4
EOG092D3OQJ	269	78	150	255	14	JTT+I+G4
EOG092D4C0N	269	80	175	247	22	JTT+I+G4
EOG092D2P7K	349	79	152	221	128	LG+I+G4
EOG092D4A4D	364	31	120	352	12	LG+F+I+G4

Supplementary Table 7: Overview of alignments of genes used for phylogenomic reconstruction. Table generated with phylociraptor. This information is also provided in the Supplementary-Data-3 file in EXCEL format. *(continued)*

gene	length	no. of sequences	no. of parsimony informative sites	no. of variable sites	no. of fixed sites	best model
EOG092D25DG	438	80	114	215	223	LG+I+G4
EOG092D0NWF	943	77	414	804	139	LG+F+I+G4
EOG092D3SKZ	274	79	104	219	55	LG+I+G4
EOG092D0DLV	840	80	272	830	10	JTT+F+I+G4
EOG092D2NES	381	81	136	243	138	LG+I+G4
EOG092D45DK	135	80	85	119	16	LG+G4
EOG092D4A5H	168	82	84	104	64	LG+I+G4
EOG092D0HLF	745	79	561	689	56	LG+I+G4
EOG092D3IIV	486	77	177	464	22	JTTDCMut+I+G4
EOG092D1EU1	469	83	79	197	272	JTT+I+G4
EOG092D46UJ	223	79	145	193	30	LG+I+G4
EOG092D4IK8	213	81	39	141	72	LG+G4
EOG092D0JYM	937	83	295	925	12	LG+F+I+G4
EOG092D4PJO	97	81	34	60	37	LG+I+G4
EOG092D33CF	457	70	212	405	52	JTT+F+I+G4
EOG092D3KPJ	274	83	69	180	94	WAG+I+G4
EOG092D2J85	506	81	187	355	151	LG+I+G4
EOG092D4NLC	151	80	68	130	21	LG+I+G4
EOG092D0I6N	904	81	409	705	199	JTT+F+I+G4
EOG092D0UJA	681	80	318	506	175	LG+F+I+G4
EOG092D1Z45	653	81	313	595	58	JTT+I+G4
EOG092D2CBO	209	71	146	191	18	WAG+I+G4
EOG092D0LTX	1116	79	485	1046	70	JTT+F+I+G4
EOG092D4SWY	79	76	48	63	16	Dayhoff+I+G4
EOG092D23QW	451	80	131	189	262	LG+I+G4
EOG092D0CKP	913	79	558	790	123	LG+I+G4
EOG092D0LTY	1185	77	556	1087	98	JTT+F+I+G4
EOG092D0TDX	930	61	348	905	25	JTT+I+G4
EOG092D1TP5	1004	80	416	853	151	JTTDCMut+F+I+G4
EOG092D0877	1314	81	497	1285	29	JTT+F+I+G4
EOG092D0HY6	676	64	412	654	22	LG+I+G4
EOG092D1CX4	753	83	360	721	32	JTT+I+G4
EOG092D26CK	1531	72	146	1524	7	JTT+F+I+G4
EOG092D3D9K	402	80	120	356	46	Dayhoff+I+G4
EOG092D4497	226	82	103	171	55	LG+G4
EOG092D0OG6	809	75	480	752	57	LG+F+I+G4
EOG092D3TRJ	188	79	98	153	35	LG+I+G4
EOG092D4HQ1	126	73	92	114	12	LG+I+G4
EOG092D2D6B	475	80	256	402	73	LG+I+G4
EOG092D2YQC	317	80	226	280	37	LG+I+G4
EOG092D44UA	238	80	117	221	17	LG+I+G4
EOG092D2K9N	1191	82	270	1112	79	JTT+F+I+G4
EOG092D2EKA	420	79	69	146	274	JTTDCMut+I+G4
EOG092D1HJN	985	83	183	939	46	JTT+F+I+G4
EOG092D08DO	901	81	507	788	113	LG+I+G4
EOG092D0Y58	912	81	417	790	122	JTT+I+G4
EOG092D2L06	321	79	166	302	19	JTTDCMut+I+G4
EOG092D3OYK	331	81	160	304	27	LG+F+I+G4
EOG092D136Q	834	80	401	731	103	LG+I+G4
EOG092D2IC5	335	77	177	238	97	LG+I+G4
EOG092D4FLZ	168	80	108	141	27	LG+I+G4
EOG092D0HUI	1002	80	650	812	190	LG+I+G4
EOG092D3ASG	317	80	134	257	60	LG+I+G4
EOG092D26R7	396	77	150	318	78	JTT+I+G4
EOG092D0OJS	859	82	360	809	50	JTT+I+G4
EOG092D27S3	393	82	149	216	177	LG+I+G4

Supplementary Table 7: Overview of alignments of genes used for phylogenomic reconstruction. Table generated with phylociraptor. This information is also provided in the Supplementary-Data-3 file in EXCEL format. *(continued)*

gene	length	no. of sequences	no. of parsimony informative sites	no. of variable sites	no. of fixed sites	best model
EOG092D10XC	600	70	252	518	82	LG+I+G4
EOG092D3ZQD	381	82	228	324	57	LG+I+G4
EOG092D4CWI	158	77	46	101	57	LG+I+G4
EOG092D4D5H	233	80	84	154	79	LG+G4
EOG092D2DDI	499	77	305	400	99	LG+F+I+G4
EOG092D0BYP	1219	78	531	1000	219	JTT+F+I+G4
EOG092D2SPT	538	80	264	475	63	JTTDCMut+I+G4
EOG092D1TTT	446	83	148	242	204	JTT+I+G4
EOG092D1H11	318	81	130	249	69	LG+F+I+G4
EOG092D12Y0	1041	82	498	850	191	LG+F+I+G4
EOG092D0FJ0	967	80	397	876	91	JTTDCMut+I+G4
EOG092D19VF	571	82	170	305	266	LG+I+G4
EOG092D4D41	189	78	94	168	21	LG+I+G4
EOG092D2NAB	565	81	284	508	57	LG+I+G4
EOG092D4A0Q	149	81	92	120	29	LG+I+G4
EOG092D34V3	615	72	353	604	11	LG+F+I+G4
EOG092D1UVP	514	81	176	293	221	LG+I+G4
EOG092D2CO6	626	80	157	620	6	JTTDCMut+I+G4
EOG092D4AE2	184	80	97	179	5	LG+I+G4
EOG092D2TE4	421	75	176	394	27	JTTDCMut+F+I+G4
EOG092D2BH7	460	78	250	426	34	JTT+F+I+G4
EOG092D4CIY	202	72	69	189	13	JTT+I+G4
EOG092D483D	173	80	120	157	16	LG+G4
EOG092D1NX0	558	80	313	460	98	LG+F+I+G4
EOG092D1CL2	604	82	267	527	77	JTT+F+I+G4
EOG092D3KT6	484	80	49	363	121	LG+F+I+G4
EOG092D2C3H	419	72	276	382	37	LG+I+G4
EOG092D1B2T	736	78	289	732	4	JTT+F+I+G4
EOG092D1SC1	529	79	294	402	127	LG+I+G4
EOG092D0FF8	960	81	381	950	10	JTT+I+G4
EOG092D181M	672	83	211	643	29	JTT+I+G4
EOG092D4IDA	192	83	132	176	16	LG+I+G4
EOG092D24UI	588	67	349	472	116	LG+I+G4
EOG092D3D5H	388	78	264	355	33	LG+F+I+G4
EOG092D3UZB	422	82	228	382	40	WAG+I+G4
EOG092D0JYR	842	81	204	773	69	JTT+F+I+G4
EOG092D03RC	1377	83	747	1180	197	LG+F+I+G4
EOG092D4AAG	272	80	60	260	12	JTTDCMut+F+I+G4
EOG092D0MFC	805	64	476	700	105	LG+I+G4
EOG092D01QP	1902	82	757	1807	95	JTT+F+I+G4
EOG092D45SG	244	81	142	223	21	LG+I+G4
EOG092D3MZ3	294	82	160	233	61	LG+I+G4
EOG092D3GX9	289	76	167	283	6	LG+I+G4
EOG092D0PK7	501	78	385	474	27	LG+I+G4
EOG092D2B4A	487	80	236	363	124	JTTDCMut+I+G4
EOG092D1ZGV	546	77	274	517	29	LG+I+G4
EOG092D41FH	339	81	199	325	14	LG+I+G4
EOG092D3VL7	155	80	102	137	18	LG+I+G4
EOG092D01ZK	1450	81	450	1440	10	JTT+F+I+G4
EOG092D02MN	1712	82	691	1680	32	LG+F+I+G4
EOG092D2X9K	1006	82	92	957	49	JTT+F+I+G4
EOG092D3F1T	227	78	149	210	17	LG+I+G4
EOG092D44X0	137	75	42	122	15	LG+F+I+G4
EOG092D2WL3	566	80	271	548	18	JTTDCMut+I+G4
EOG092D06C2	1197	75	852	1164	33	JTT+F+I+G4
EOG092D3SWM	202	60	52	126	76	LG+G4

Supplementary Table 7: Overview of alignments of genes used for phylogenomic reconstruction. Table generated with phylociraptor. This information is also provided in the Supplementary-Data-3 file in EXCEL format. *(continued)*

gene	length	no. of sequences	no. of parsimony informative sites	no. of variable sites	no. of fixed sites	best model
EOG092D08R4	1072	82	511	909	163	LG+I+G4
EOG092D3IQI	448	69	224	432	16	LG+I+G4
EOG092D31TX	351	80	154	288	63	JTT+F+I+G4
EOG092D4PES	122	67	84	109	13	LG+I+G4
EOG092D4877	161	76	111	139	22	LG+I+G4
EOG092D06BS	1368	78	1060	1295	73	JTTDCMut+I+G4
EOG092D4HIU	214	82	57	204	10	LG+F+G4
EOG092D1DJJ	654	77	330	540	114	LG+I+G4
EOG092D409L	222	82	114	179	43	LG+I+G4
EOG092D0BFU	1093	83	661	885	208	LG+F+I+G4
EOG092D2ACV	512	79	165	312	200	JTT+I+G4
EOG092D1C4I	500	70	244	398	102	LG+I+G4
EOG092D1HSA	603	83	229	536	67	LG+F+I+G4
EOG092D1BH6	553	78	179	349	204	LG+I+G4
EOG092D0AI2	976	83	503	836	140	JTT+I+G4
EOG092D12N2	608	80	392	527	81	LG+I+G4
EOG092D1SQB	748	82	424	654	94	JTT+I+G4
EOG092D0HBI	912	80	464	643	269	JTTDCMut+F+I+G4
EOG092D0Q8X	996	81	451	883	113	LG+F+I+G4
EOG092D03NE	1885	71	675	1870	15	JTT+F+I+G4
EOG092D3VOQ	283	80	177	235	48	Dayhoff+I+G4
EOG092D0564	1331	82	977	1235	96	LG+I+G4
EOG092D48PX	188	78	124	157	31	LG+I+G4
EOG092D3HV9	332	79	176	294	38	LG+I+G4
EOG092D2SV5	351	80	136	339	12	JTT+F+I+G4
EOG092D1RSV	597	78	249	533	64	LG+I+G4
EOG092D35RA	517	80	262	437	80	LG+F+I+G4
EOG092D182U	1198	67	337	1169	29	JTT+I+G4
EOG092D3RVC	202	81	54	171	31	JTTDCMut+F+I+G4
EOG092D34F6	810	82	363	702	108	JTT+I+G4
EOG092D36TR	988	81	644	951	37	JTT+I+G4
EOG092D0951	964	81	432	738	226	LG+I+G4
EOG092D2IG4	453	80	268	447	6	LG+I+G4
EOG092D2V2V	451	72	257	398	53	LG+F+I+G4
EOG092D34RI	286	82	154	240	46	LG+I+G4
EOG092D3ZTV	267	82	165	248	19	LG+I+G4
EOG092D3TZJ	413	83	129	344	69	LG+F+I+G4
EOG092D0QW3	1090	57	544	1004	86	LG+F+I+G4
EOG092D45Q9	165	21	76	118	47	LG+G4
EOG092D3SSR	222	72	153	194	28	LG+I+G4
EOG092D0BA6	1354	82	462	1185	169	JTT+I+G4
EOG092D4U2S	119	82	64	103	16	LG+I+G4
EOG092D3QDK	251	79	119	211	40	LG+I+G4
EOG092D1HYF	638	80	225	578	60	LG+I+G4
EOG092D3MYJ	263	80	45	78	185	DCMut+F+I+G4
EOG092D4KGF	298	77	182	287	11	LG+I+G4
EOG092D46GG	159	79	77	130	29	LG+I+G4
EOG092D2OSE	392	81	182	298	94	LG+I+G4
EOG092D0S7U	745	82	177	699	46	LG+I+G4
EOG092D04FZ	1786	72	613	1779	7	JTT+F+I+G4
EOG092D2J1E	889	80	329	830	59	JTT+I+G4
EOG092D4JJW	116	81	48	75	41	LG+G4
EOG092D4M39	166	70	49	143	23	JTT+I+G4
EOG092D1LUK	660	81	273	453	207	JTTDCMut+I+G4
EOG092D3IT4	376	82	203	355	21	JTTDCMut+I+G4
EOG092D4LPB	194	74	63	171	23	JTTDCMut+I+G4

Supplementary Table 7: Overview of alignments of genes used for phylogenomic reconstruction. Table generated with phylociraptor. This information is also provided in the Supplementary-Data-3 file in EXCEL format. *(continued)*

gene	length	no. of sequences	no. of parsimony informative sites	no. of variable sites	no. of fixed sites	best model
EOG092D4K5O	116	78	49	74	42	LG+G4
EOG092D0RY3	1097	81	511	1050	47	JTT+F+I+G4
EOG092D4199	184	76	108	153	31	LG+G4
EOG092D3X0O	299	80	154	269	30	LG+I+G4
EOG092D0CTT	1571	83	665	1523	48	JTT+F+I+G4
EOG092D0R2T	728	80	428	636	92	LG+I+G4
EOG092D3OJ2	255	77	44	95	160	JTTDCMut+I+G4
EOG092D3NPG	250	79	55	98	152	LG+I+G4
EOG092D0R62	870	83	256	813	57	JTTDCMut+F+I+G4
EOG092D05PP	1173	82	473	1114	59	JTT+I+G4
EOG092D3RKB	283	81	118	239	44	LG+I+G4
EOG092D07QT	1127	82	318	671	456	LG+I+G4
EOG092D042R	1298	80	857	1279	19	JTT+F+I+G4
EOG092D3VNW	197	75	127	171	26	LG+G4
EOG092D4DJ2	283	82	98	275	8	JTT+I+G4
EOG092D16QX	624	72	299	556	68	LG+I+G4
EOG092D1ZWB	456	74	188	388	68	LG+F+I+G4
EOG092D1UOB	562	81	279	449	113	JTTDCMut+I+G4
EOG092D1I2I	399	81	207	389	10	LG+I+G4
EOG092D2E2U	599	81	218	514	85	JTT+F+I+G4
EOG092D13SA	654	83	276	509	145	JTTDCMut+I+G4
EOG092D47YD	241	79	190	221	20	JTTDCMut+I+G4
EOG092D22TO	483	81	229	461	22	LG+F+I+G4
EOG092D37RT	171	81	112	143	28	LG+I+G4
EOG092D13G3	599	78	372	516	83	LG+I+G4
EOG092D3KBF	247	75	125	183	64	LG+I+G4
EOG092D167J	503	79	271	420	83	JTT+I+G4
EOG092D2W4H	374	81	169	240	134	LG+I+G4
EOG092D4DWW	251	55	110	246	5	JTT+I+G4
EOG092D0EC9	1473	81	650	1288	185	LG+I+G4
EOG092D4GNM	191	74	105	187	4	LG+I+G4
EOG092D2SCW	351	81	190	305	46	LG+I+G4
EOG092D3HQ3	280	81	108	244	36	LG+F+I+G4
EOG092D2HE0	492	79	203	415	77	LG+I+G4
EOG092D4HZE	122	79	79	100	22	LG+I+G4
EOG092D3NQF	420	62	190	404	16	LG+F+I+G4
EOG092D18W8	572	75	304	512	60	LG+F+I+G4
EOG092D1ML3	560	80	219	533	27	LG+F+G4
EOG092D4AOP	216	80	130	195	21	JTTDCMut+I+G4
EOG092D3942	716	78	379	675	41	JTT+F+I+G4
EOG092D1V0I	540	81	209	323	217	LG+I+G4
EOG092D2RBH	347	82	222	298	49	LG+I+G4
EOG092D21TB	520	80	292	449	71	LG+I+G4
EOG092D4GQ5	240	82	145	210	30	LG+I+G4
EOG092D42PJ	220	81	139	177	43	LG+I+G4
EOG092D3LA0	295	82	185	280	15	LG+I+G4
EOG092D092M	1070	82	491	726	344	LG+I+G4
EOG092D4679	176	81	119	165	11	JTT+I+G4
EOG092D20HF	604	79	391	538	66	JTTDCMut+I+G4
EOG092D2Z0K	613	80	246	571	42	JTT+F+I+G4
EOG092D0KIC	997	81	539	766	231	JTTDCMut+I+G4
EOG092D3QQQ	1606	83	269	1554	52	JTT+F+I+G4
EOG092D0GS4	1026	80	426	969	57	JTT+F+I+G4
EOG092D2502	428	77	308	383	45	LG+I+G4
EOG092D3GZM	200	81	107	161	39	LG+G4
EOG092D0OYE	998	82	457	909	89	JTT+I+G4

Supplementary Table 7: Overview of alignments of genes used for phylogenomic reconstruction. Table generated with phylociraptor. This information is also provided in the Supplementary-Data-3 file in EXCEL format. *(continued)*

gene	length	no. of sequences	no. of parsimony informative sites	no. of variable sites	no. of fixed sites	best model
EOG092D0HR5	670	77	373	642	28	JTT+F+I+G4
EOG092D2A2K	908	81	275	798	110	LG+F+I+G4
EOG092D28W9	969	79	477	914	55	JTT+F+I+G4
EOG092D1M7G	760	81	394	677	83	JTTDCMut+I+G4
EOG092D06WH	521	82	273	465	56	LG+I+G4
EOG092D326X	589	82	116	583	6	JTT+I+G4
EOG092D3F3H	720	80	148	702	18	JTT+F+I+G4
EOG092D3TIF	310	81	139	231	79	LG+F+I+G4
EOG092D4JCC	151	59	71	133	18	LG+G4
EOG092D1OF2	444	81	293	413	31	LG+F+I+G4
EOG092D14TO	846	73	203	812	34	JTT+F+I+G4
EOG092D1LYU	549	79	349	488	61	LG+I+G4
EOG092D3SXY	297	82	150	283	14	LG+F+I+G4
EOG092D2Y6K	317	78	172	225	92	LG+I+G4
EOG092D25D9	378	72	248	352	26	LG+I+G4
EOG092D3CIT	216	74	133	193	23	WAG+I+G4
EOG092D0SSQ	1627	81	261	1378	249	JTTDCMut+I+G4
EOG092D1BBT	795	82	250	738	57	JTT+I+G4
EOG092D3IQU	247	81	86	164	83	LG+F+I+G4
EOG092D0R2H	819	82	328	770	49	JTT+F+I+G4
EOG092D1L93	494	80	259	407	87	LG+I+G4
EOG092D2BBJ	531	81	245	461	70	JTTDCMut+I+G4
EOG092D3076	349	79	197	238	111	LG+I+G4
EOG092D26XB	394	72	216	369	25	JTTDCMut+I+G4
EOG092D2RFS	282	75	214	261	21	LG+I+G4

Supplementary Table 8: Gene family expansion analysis summary for different runs

Supplementary Table 8: Table summarizing singinficantly expanded CAZyme families in different CAFE5 runs.

run	rate	error_model	expanded_families
1	single_rate		GH43:p=0.004 AA3:p=0 CE10:p=0 AA7:p=0
2	single_rate	yes	GH43:p=0 AA9:p=0.014 AA3:p=0 CE10:p=0 AA7:p=0
3	single_rate		GH43:p=0.005 AA3:p=0 CE10:p=0 AA7:p=0
4	single_rate	yes	GH43:p=0 AA9:p=0.016 AA3:p=0 CE10:p=0 AA7:p=0
5	single_rate		GH43:p=0.006 AA3:p=0 CE10:p=0 AA7:p=0
6	single_rate	yes	GH43:p=0.001 AA9:p=0.018 AA3:p=0 CE10:p=0 AA7:p=0
7	single_rate		GH43:p=0.008 AA3:p=0 CE10:p=0 AA7:p=0
8	single_rate	yes	GH43:p=0 AA9:p=0.013 AA3:p=0 CE10:p=0 AA7:p=0
9	single_rate		GH43:p=0.006 AA3:p=0 CE10:p=0 AA7:p=0
10	single_rate	yes	GH43:p=0 AA9:p=0.023 AA3:p=0 CE10:p=0 AA7:p=0
11	two_rates		GH43:p=0.005 AA3:p=0 CE10:p=0 AA7:p=0
12	two_rates	yes	GH18:p=0.039 GH43:p=0.001 AA9:p=0.021 AA3:p=0 CE10:p=0 AA7:p=0
13	two_rates		GH43:p=0.006 AA3:p=0 CE10:p=0 AA7:p=0
14	two_rates	yes	GH18:p=0.041 GH43:p=0 AA9:p=0.019 AA3:p=0 CE10:p=0 AA7:p=0
15	two_rates		GH43:p=0.006 AA3:p=0 CE10:p=0 AA7:p=0
16	two_rates	yes	GH43:p=0.001 AA9:p=0.024 AA3:p=0 CE10:p=0 AA7:p=0
17	two_rates		GH43:p=0.007 AA3:p=0 CE10:p=0 AA7:p=0

Supplementary Table 8: Table summarizing singinficantly expanded CAZyme families in different CAFE5 runs. *(continued)*

run	rate	error_model	expanded_families
18	two_rates	yes	GH18:p=0.045 GH43:p=0.001 AA9:p=0.027 AA3:p=0 CE10:p=0 AA7:p=0
19	two_rates		GH43:p=0.006 AA3:p=0 CE10:p=0 AA7:p=0
20	two_rates	yes	GH18:p=0.047 GH43:p=0.001 AA9:p=0.021 AA3:p=0 CE10:p=0 AA7:p=0

Supplementary Table 9, 10 and 11: Comparison of CAZyme number of LFS groups and other fungi

Supplementary Table 9: Mean total number of genes found in different groups of genomes following their phylogenetic placement in Figure 1 for different CAZyme classes. Relative difference (%) between gene numbers calculated for LFS groups and other Ascomycete groups included in this study.

CAZyme	Lecanoromycetes (mean)	Lecanoromycetidae (mean)	Ostropomycetidae (mean)	Ostropomycetidae w/o OG clade (mean)	OG clade (mean)	other fungi (mean)	diff. Lecanoromycetes vs. other fungi (%)	diff. Lecanoromycetidae vs. other fungi (%)	diff. Ostropomycetidae vs. other fungi (%)	diff. Ostropomycetidae (w/o OG clade) vs. other fungi (%)	diff. OG clade vs. other fungi (%)	diff. Lecanoromycetidae vs. Ostropomycetidae (%)
AA	40.739	41.045	40.458	34.421	63.400	63.297	43.366	42.651	44.025	59.101	0.162	1.441
CBM	4.457	4.318	4.583	3.789	7.600	9.568	72.890	75.608	70.444	86.518	22.922	5.957
CE	24.391	19.773	28.625	22.947	50.200	43.405	56.092	74.813	41.039	61.664	14.518	36.581
GH	100.326	84.318	115.000	98.737	176.800	169.162	51.087	66.943	38.121	52.576	4.415	30.787
GT	64.196	64.182	64.208	64.684	62.400	67.243	4.637	4.659	4.618	3.879	7.472	0.041
PL	0.457	0.091	0.792	0.105	3.400	6.784	174.779	194.711	158.198	193.888	66.454	158.798

Supplementary Table 10: Statistical comparison of mean total numbers of genes found in different groups of genomes following their phylogenetic placement in Figure 1 for different CAZyme classes. p-values for between group comparison were calculated using two-sided Wilcoxon rank-sum tests.

CAZyme	Lecanoromycetes (mean)	Lecanoromycetidae (mean)	Ostropomycetidae (mean)	Ostropomycetidae w/o OG clade (mean)	OG clade (mean)	other fungi (mean)	p-value Lecanoromycetes vs. other fungi	p-value Lecanoromycetidae vs. other fungi	p-value Ostropomycetidae vs. other fungi	p-value Ostropomycetidae (w/o OG clade) vs. other fungi	p-value OG clade vs. other fungi	p-value Lecanoromycetidae vs. Ostropomycetidae
AA	40.739	41.045	40.458	34.421	63.400	63.297	0.004	0.026	0.009	0.001	0.741	0.567
CBM	4.457	4.318	4.583	3.789	7.600	9.568	0.000	0.002	0.005	0.000	0.572	0.982
CE	24.391	19.773	28.625	22.947	50.200	43.405	0.000	0.000	0.011	0.001	0.351	0.024
GH	100.326	84.318	115.000	98.737	176.800	169.162	0.000	0.000	0.005	0.000	0.614	0.000
GT	64.196	64.182	64.208	64.684	62.400	67.243	0.180	0.265	0.270	0.435	0.259	0.956
PL	0.457	0.091	0.792	0.105	3.400	6.784	0.000	0.000	0.000	0.000	0.495	0.130

Supplementary Table 11: Relative increase (positive values) and decrease (negative values) of mean CAZyme numbers for different groups of genomes following their phylogenetic placement in Figure 1 and for different CAZyme classes.

CAZyme	Lecanoromycetes (mean)	Lecanoromycetidae (mean)	Ostropomycetidae (mean)	Ostropomycetidae w/o OG clade (mean)	OG clade (mean)	other fungi (mean)	incr. Lecanoromycetes vs. other fungi (%)	incr. Lecanoromycetidae vs. other fungi (%)	incr. Ostropomycetidae vs. other fungi (%)	incr. Ostropomycetidae (w/o OG clade) vs. other fungi (%)	incr. OG clade vs. other fungi (%)	incr. Lecanoromycetidae vs. Ostropomycetidae (%)
AA	40.739	41.045	40.458	34.421	63.400	63.297	-35.638	-35.154	-36.082	-45.620	0.162	-1.430
CBM	4.457	4.318	4.583	3.789	7.600	9.568	-53.421	-54.866	-52.095	-60.393	-20.565	6.140
CE	24.391	19.773	28.625	22.947	50.200	43.405	-43.806	-54.446	-34.052	-47.132	15.654	44.770
GH	100.326	84.318	115.000	98.737	176.800	169.162	-40.692	-50.155	-32.018	-41.632	4.515	36.388
GT	64.196	64.182	64.208	64.684	62.400	67.243	-4.532	-4.553	-4.513	-3.806	-7.203	0.041
PL	0.457	0.091	0.792	0.105	3.400	6.784	-93.270	-98.660	-88.330	-98.448	-49.880	770.833

Supplementary Figure 1: Metrics of studied genomes

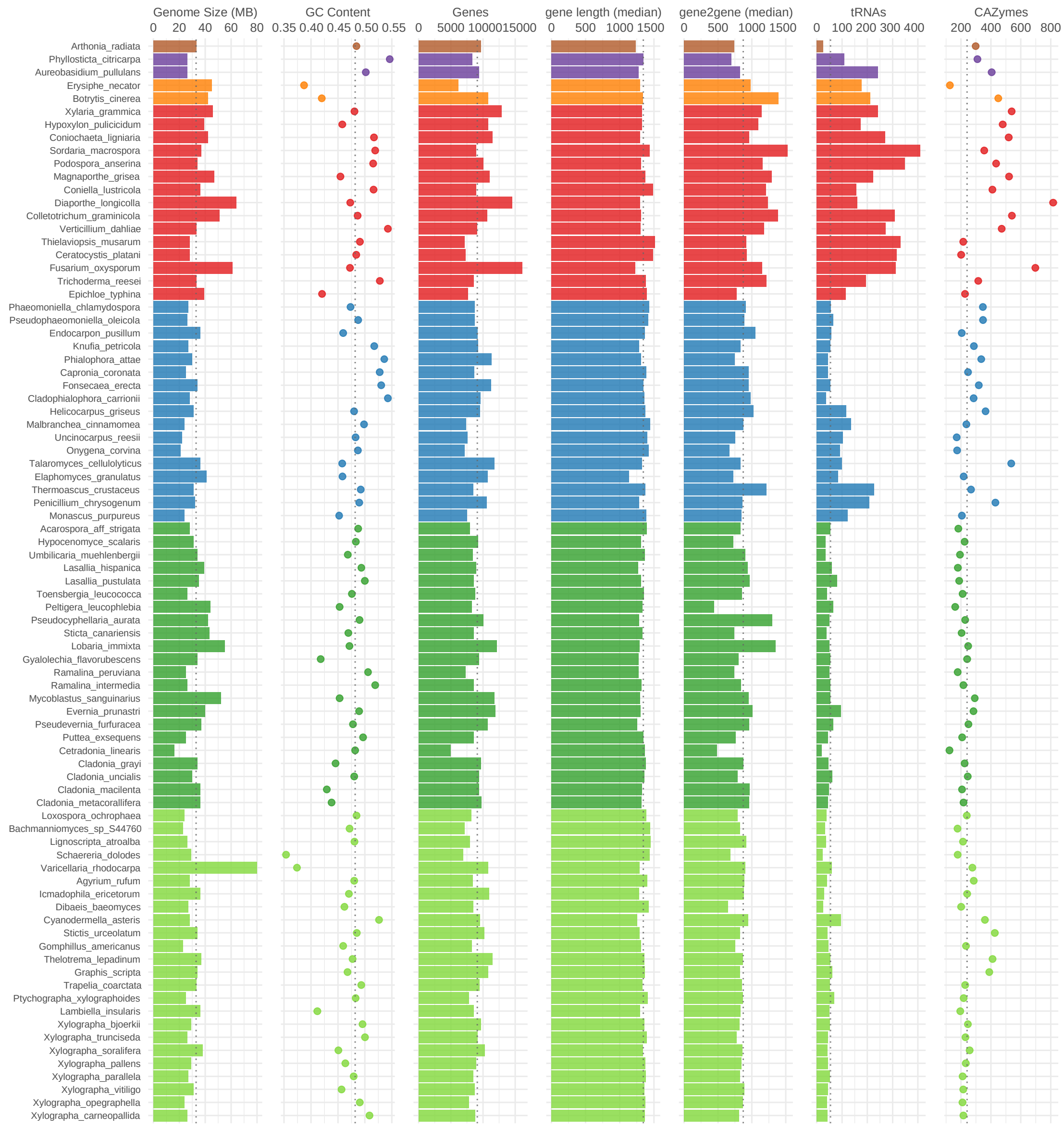

Supplementary Figure 1: Different characteristics of the 83 fungal genomes studied here. Colors refer to different taxonomic groups. Brown - Arthoniomycetes, purple - Dothideomycetes, orange - Leotiomycetes, red - Sordariomycetes, blue - Eurotiomycetes, dark green - Lecanoromycetidae, light green - Ostropomycetidae

Supplementary Figure 2: BUSCO completeness of studied genomes

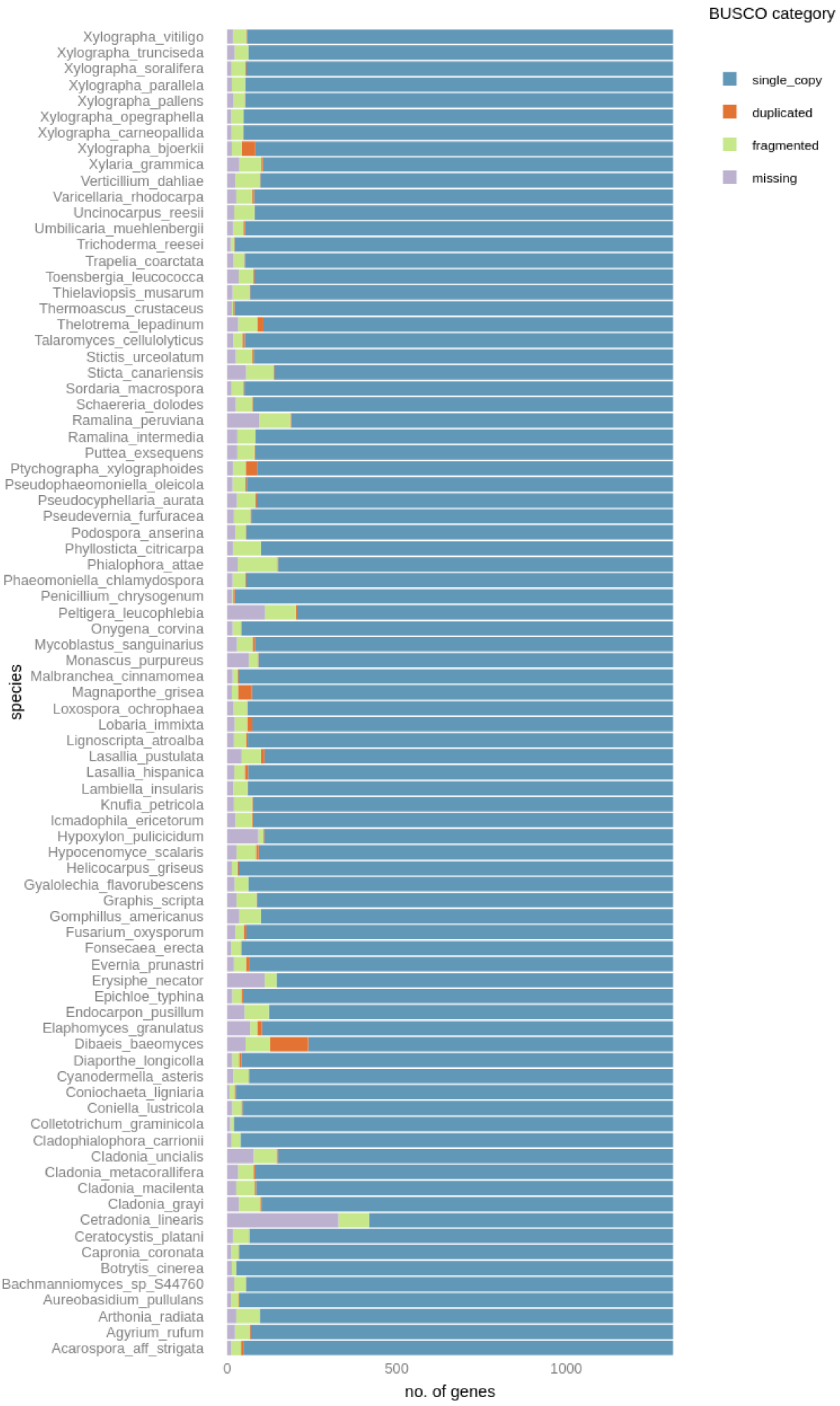

Supplementary Figure 2: BUSCO completeness of core Ascomycota genes (ascomycota_odb9) in the 83 studied genomes. Figure generated with phylociraptor.

Supplementary Figure 3 and 4: Phylogenomic trees

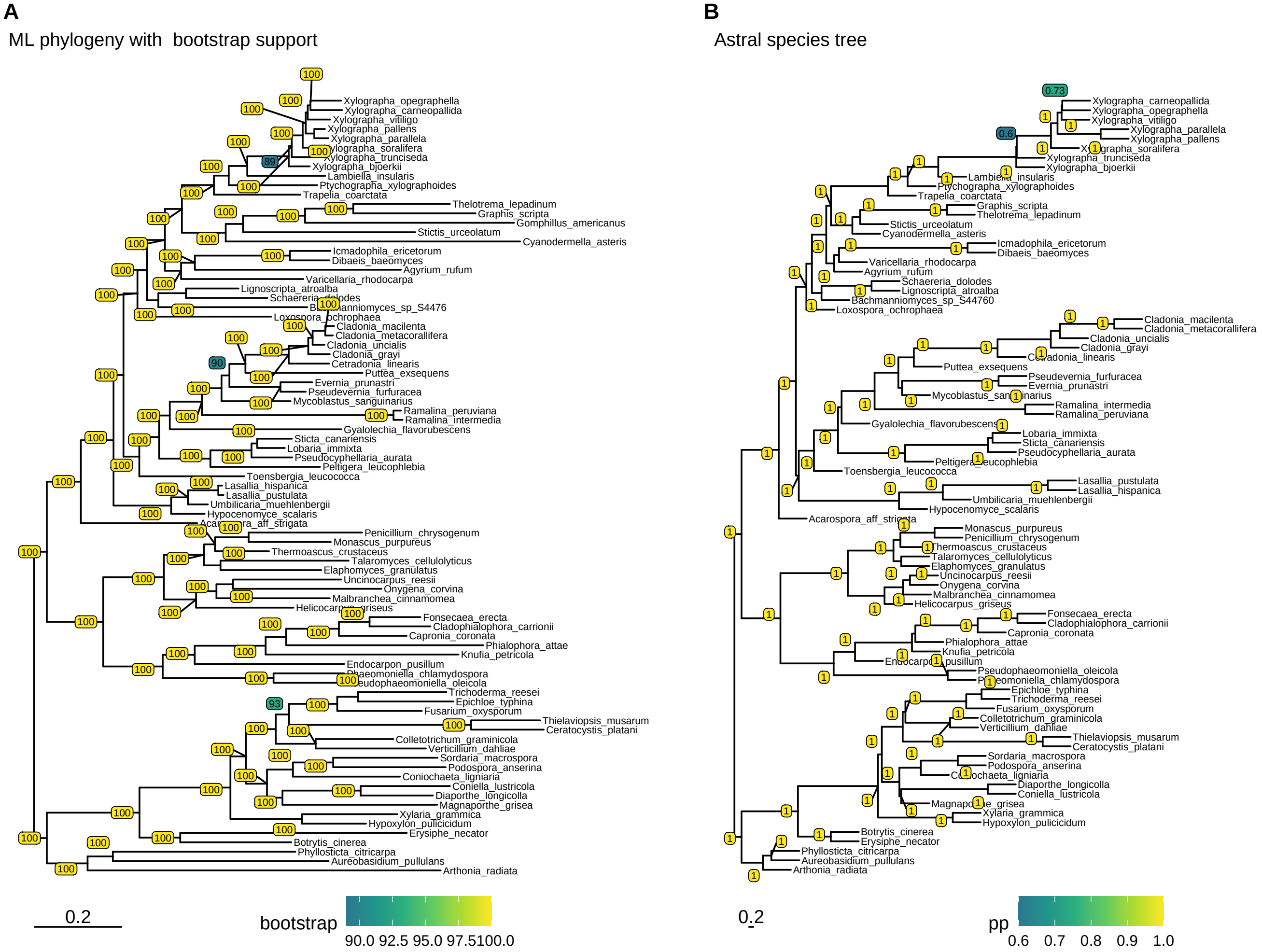

Supplementary Figure 3: Phylogenomic trees of higher Ascomycetes based on 1310 alignments of single-copy genes. A) IQ-Tree maximum-likelihood tree of a concatenated supermatrix alignment with bootstrap node-support values. B) ASTRAL species tree calculated from individual maximum-likelihood gene-trees. Node support given as Posterior Probabilities (PP).

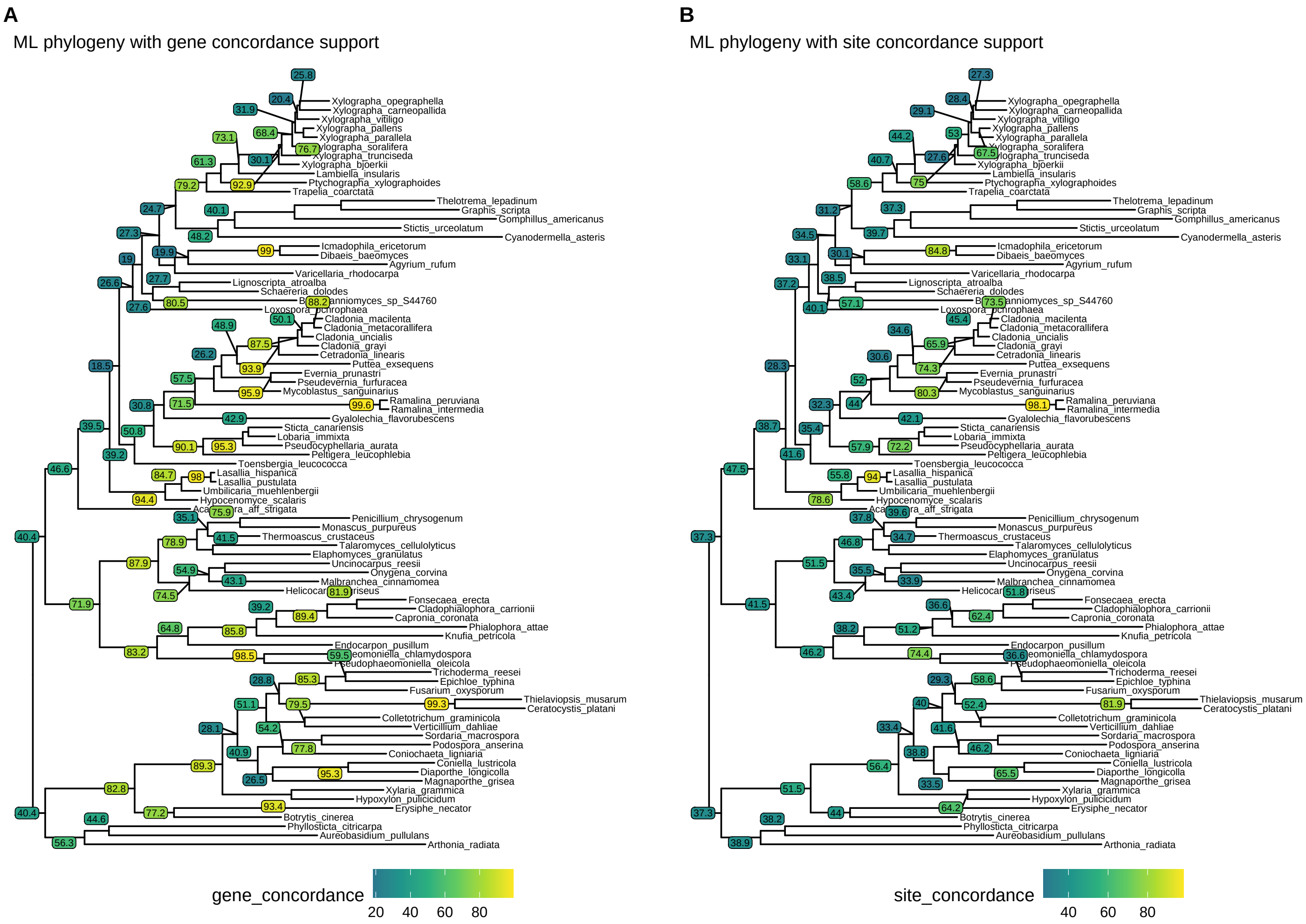

Supplementary Figure 4: Maximum-Likelihood phylogenomic tree based in a concatenated alignment of 1310 singl-copy gene alignments. Node-support given as gene-concordance and site-concordance factors calculated with IQ-Tree.

Supplementary Figure 5: Distribution of sugar-transporter orthologues

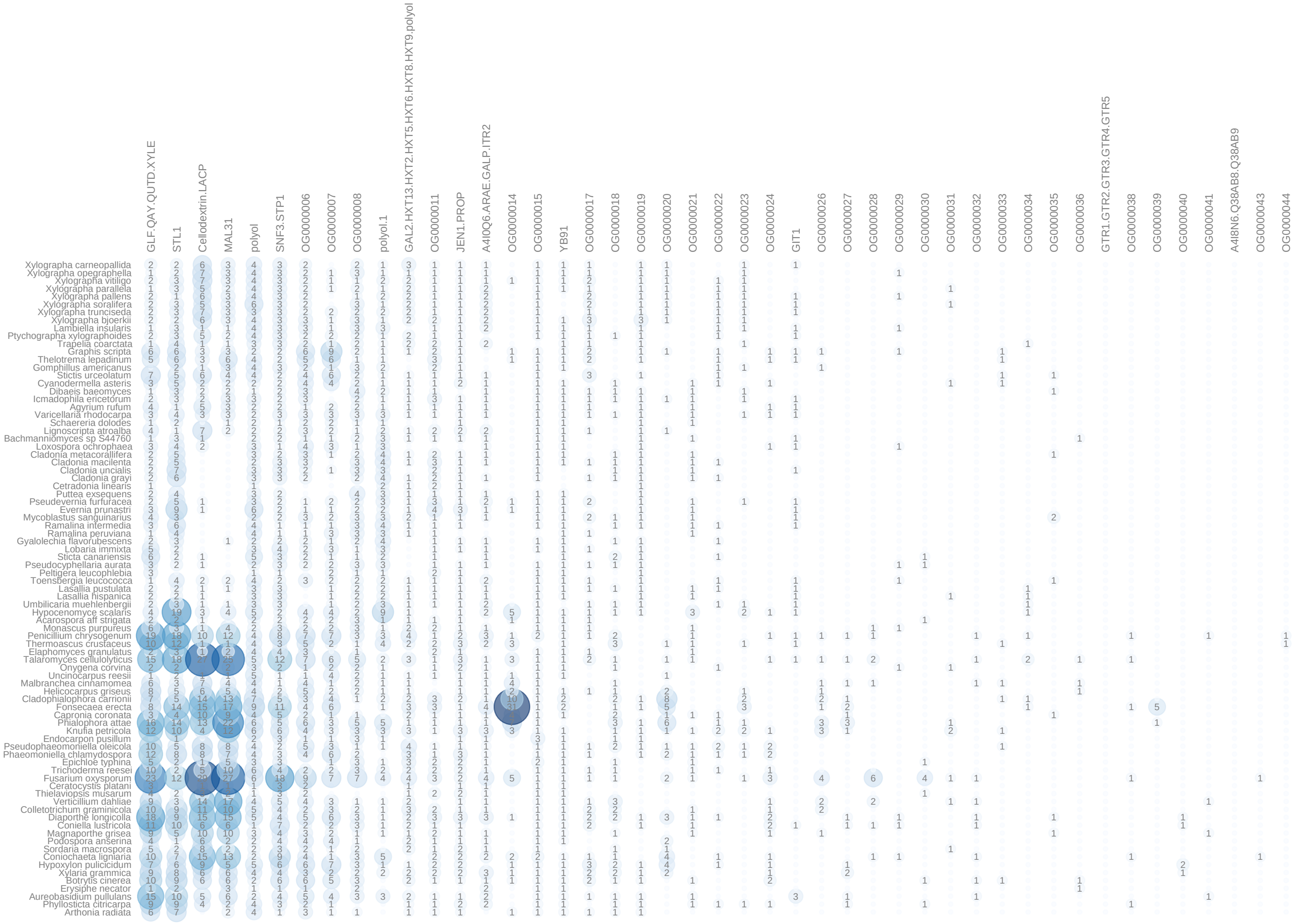

Supplementary Figure 5: Overview of sugar transporters with PF00083 annotations in 83 studied genomes. Each column represents one orthogroup inferred by Orthofinder. Names of columns were assigned based on the presence of characterized transporter sequences in the respective orthogroup. Columns without any characterized transporter sequences still have names assigned by Orthofinder.

Supplementary Figure 6: Distribution of peroxidase orthologues

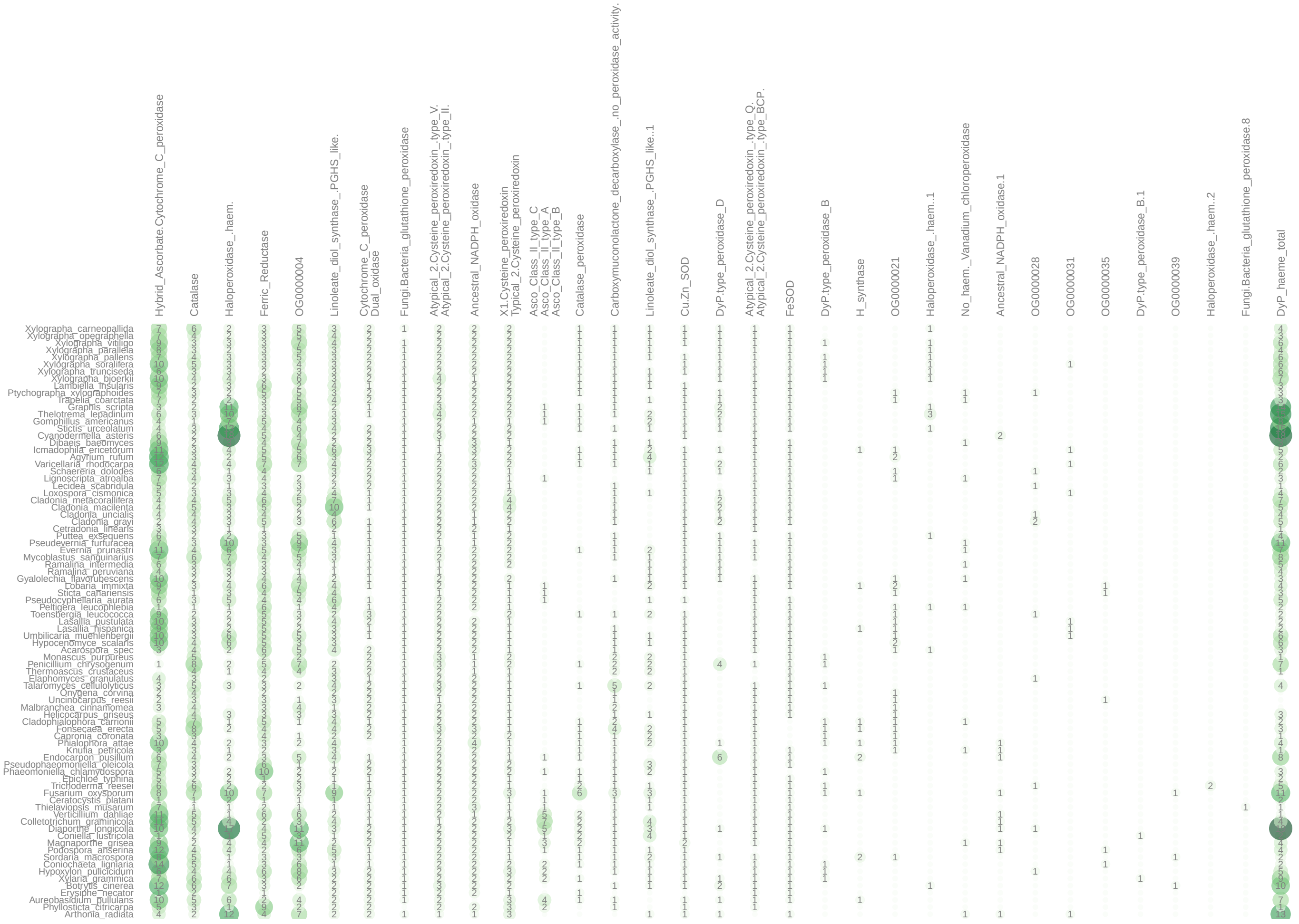

Supplementary Figure 6: Overview of fungal peroxidases downloaded from RedOxibase in 83 genomes. Each column represents one orthogroup inferred by Orthofinder. Names of columns were assigned based on the presence of characterized peroxidase sequences in the respective orthogroup. Columns without any characterized peroxidase sequences still have names assigned by Orthofinder. Peroxidases which had no orthologs in any of the 83 studied genomes were excluded from the figure.

Supplementary Figure 7: Ancestral state reconstruction results for (hemi-)cellulose degrading CAZymes

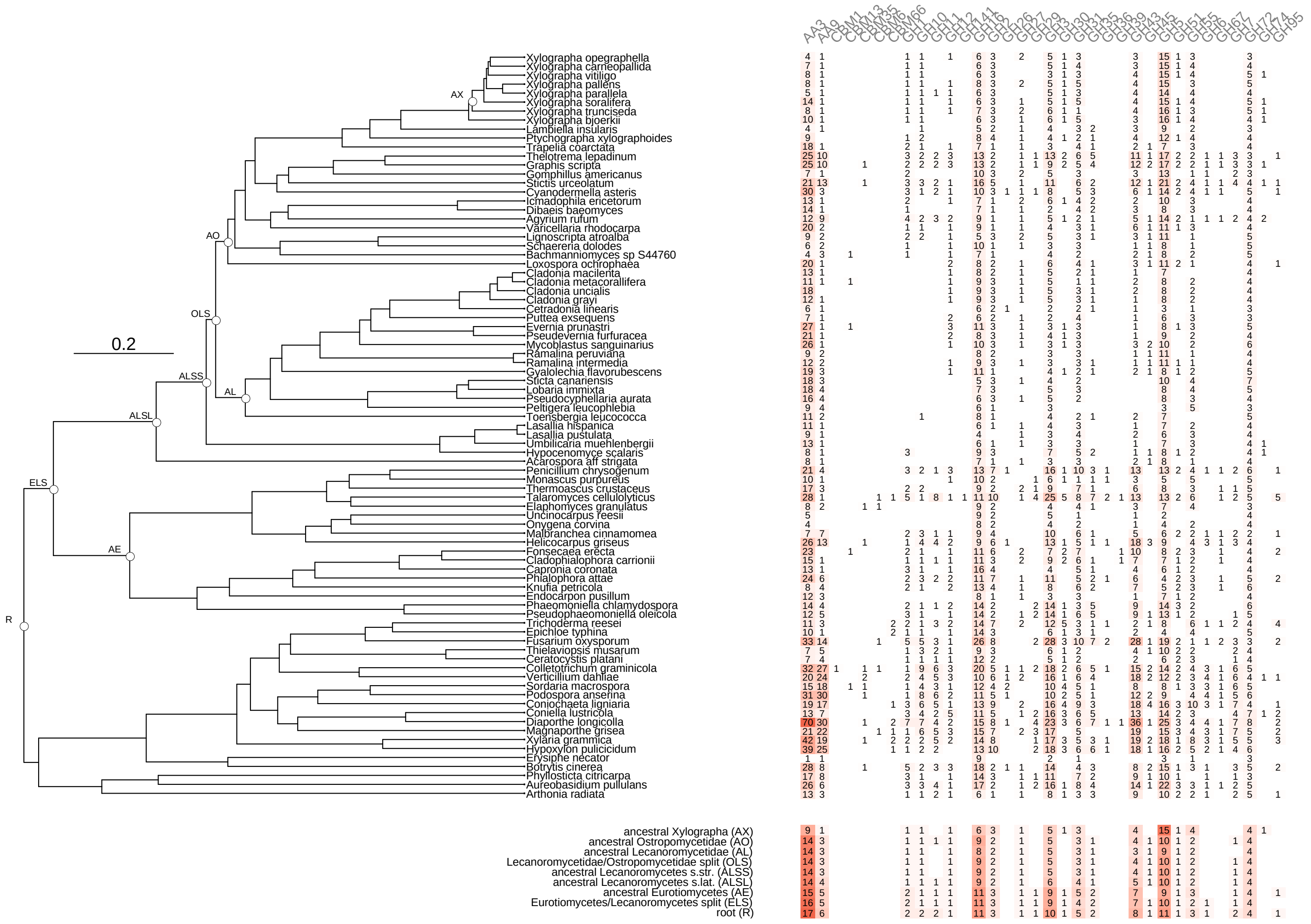

Supplementary Figure 7: Heatmap of extant CAZyme numbers of families involved in cellulose and hemicellulose breakdown according to Table 6. Values below the tree are taken from ancestral state reconstructions of family sizes at different nodes.

Supplementary Figure 8: Ancestral state reconstruction results for pectin degrading CAZymes

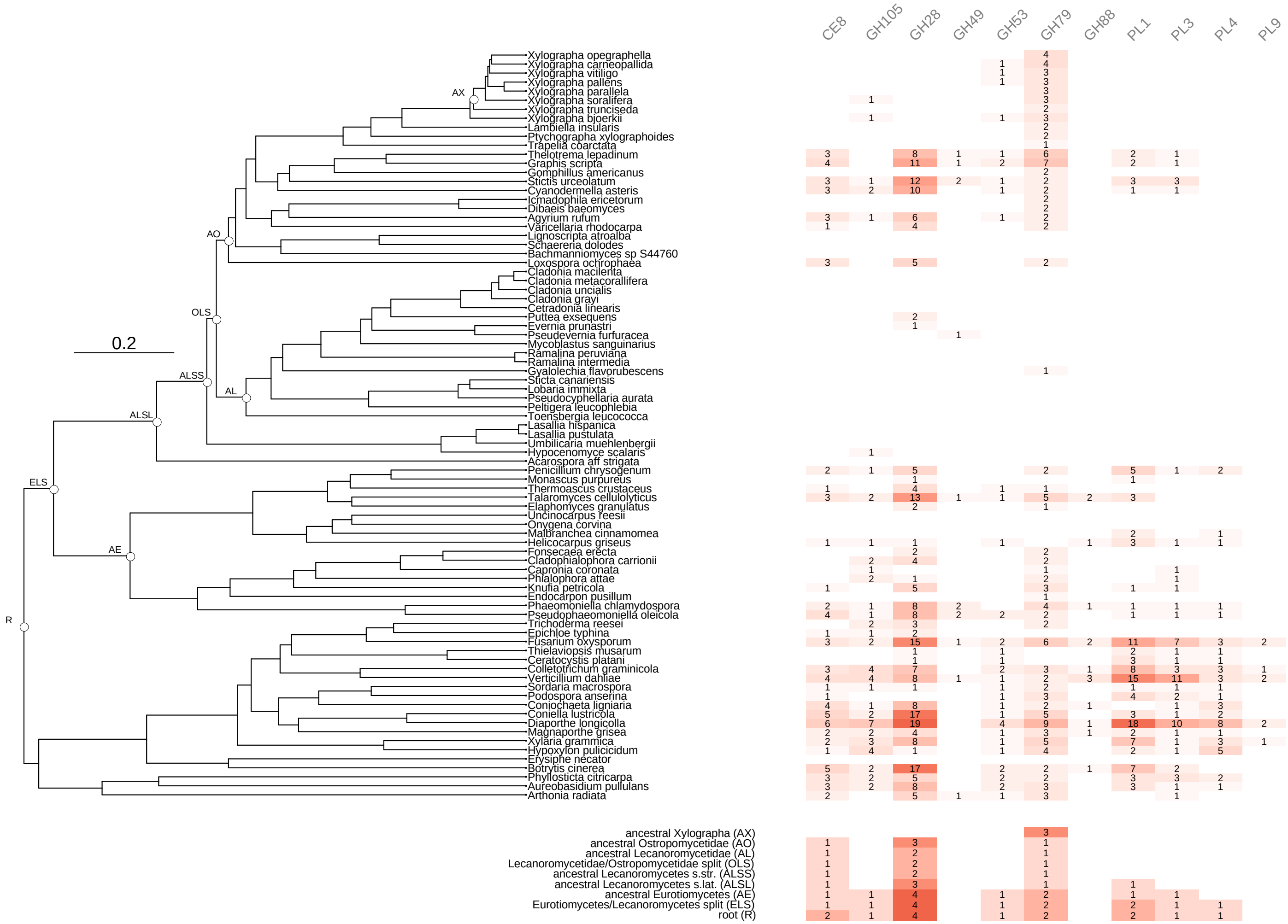

Supplementary Figure 8: Heatmap of extant CAZyme numbers of families involved in pectin breakdown according to Table 6. Values below the tree are taken from ancestral state reconstructions of family sizes at different nodes.

Supplementary Figure 9: Ancestral state reconstruction results for lignin degrading CAZymes

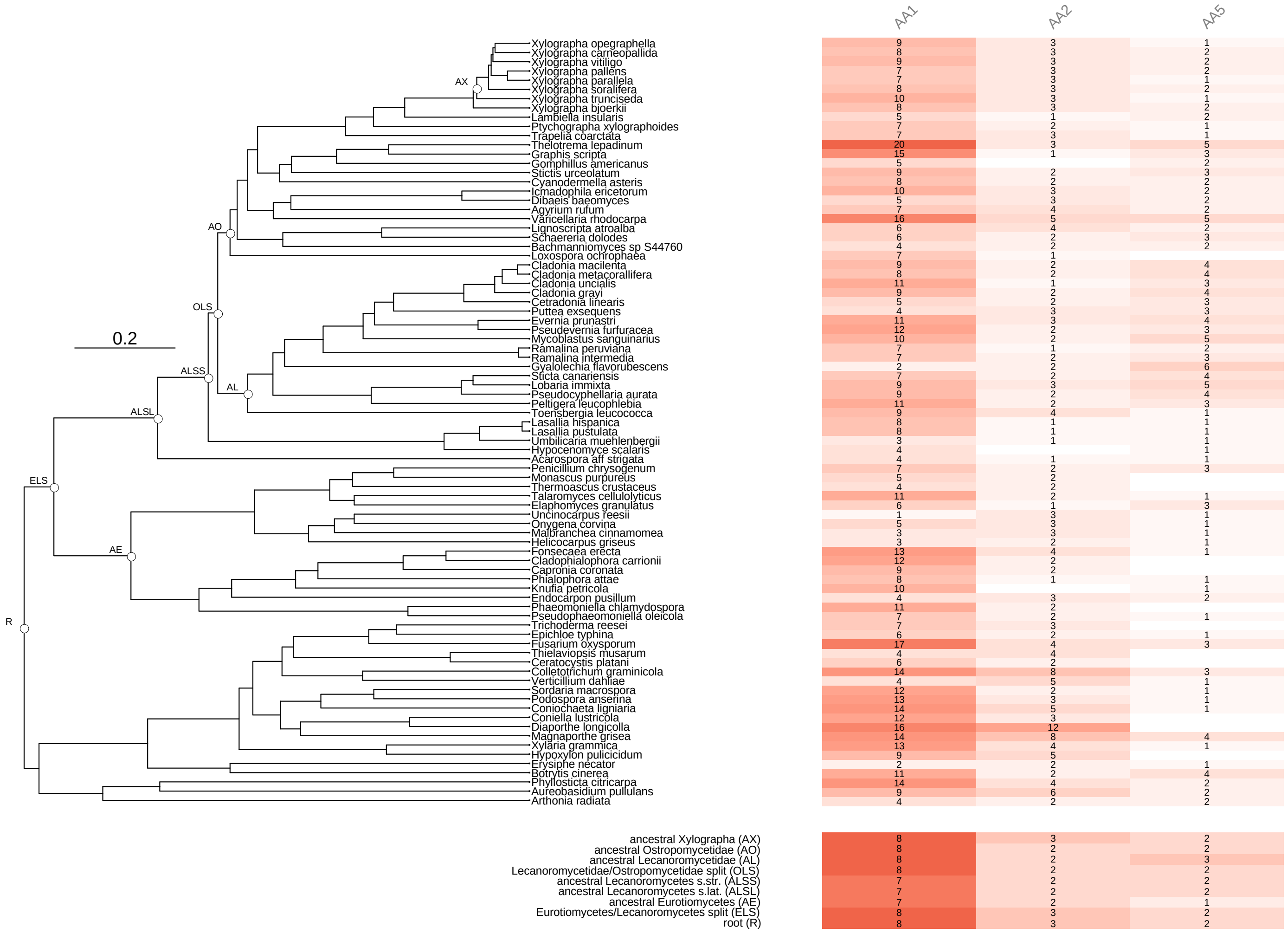

Supplementary Figure 9: Heatmap of extant CAZyme numbers of families involved in lignin breakdown according to Table 6. Values below the tree are taken from ancestral state reconstructions of family sizes at different nodes.

Supplementary Figure 10: Similarity of CAZyme sets based on PCA

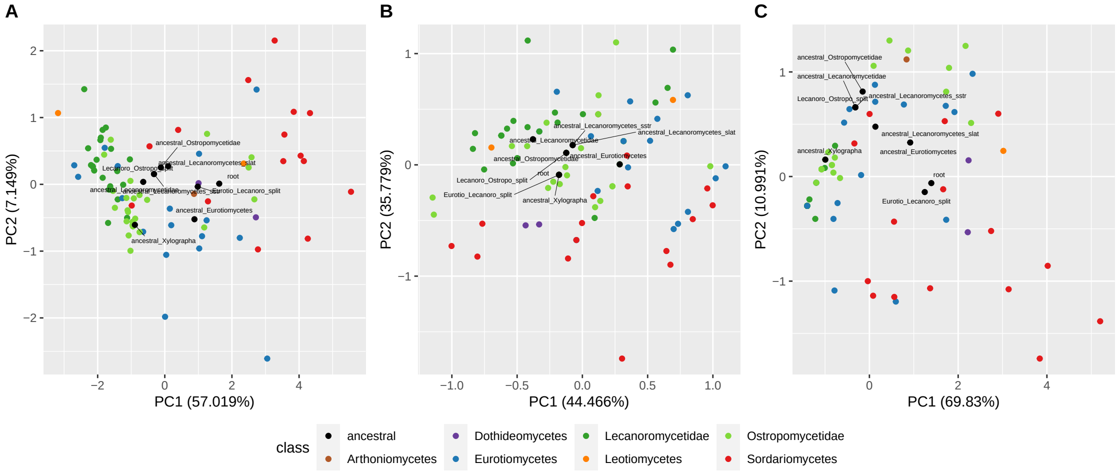

Supplementary Figure 10: Overall similarity of CAZyme sets involved in (hemi)cellulose (A), lignin(B) and pectin(C) breakdown. Each dot represents the CAZyme composition of one genome. Dots are colored by broad-scale taxonomic assignment on class-level or subclass level (Lecanoromycetes). Labeled black dots refer to the composition reconstructed at ancestral nodes.

Supplementary Figure 11: Distribution and ancestral states of all CAZyme families

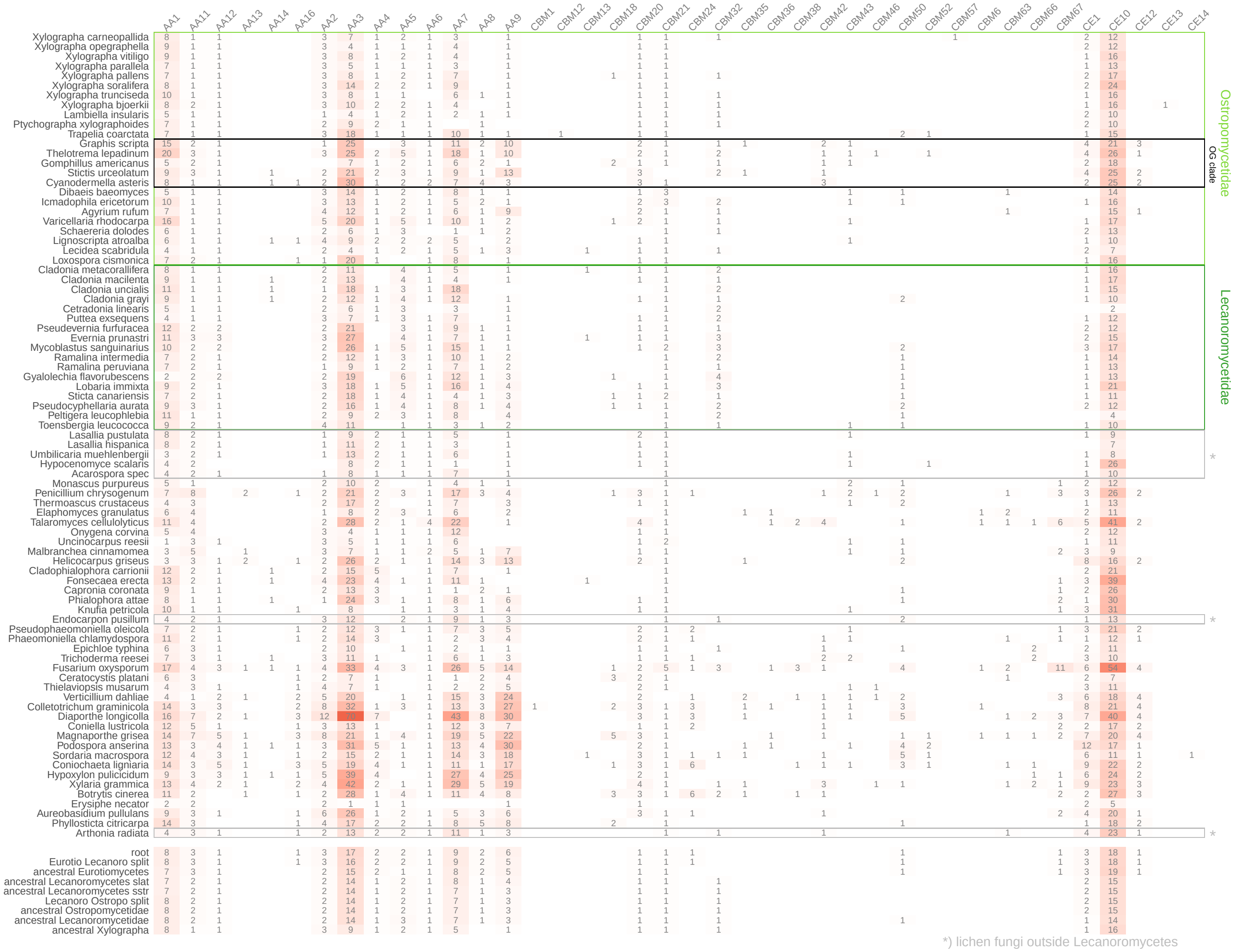

(a) part 1 of 5

Supplementary Figure 11: Heatmap of gene numbers of all CAZyme families recovered in 83 genomes analyzed in this study and for ancestral nodes according to Figure 1. Darker colors indicate higher number of genes.

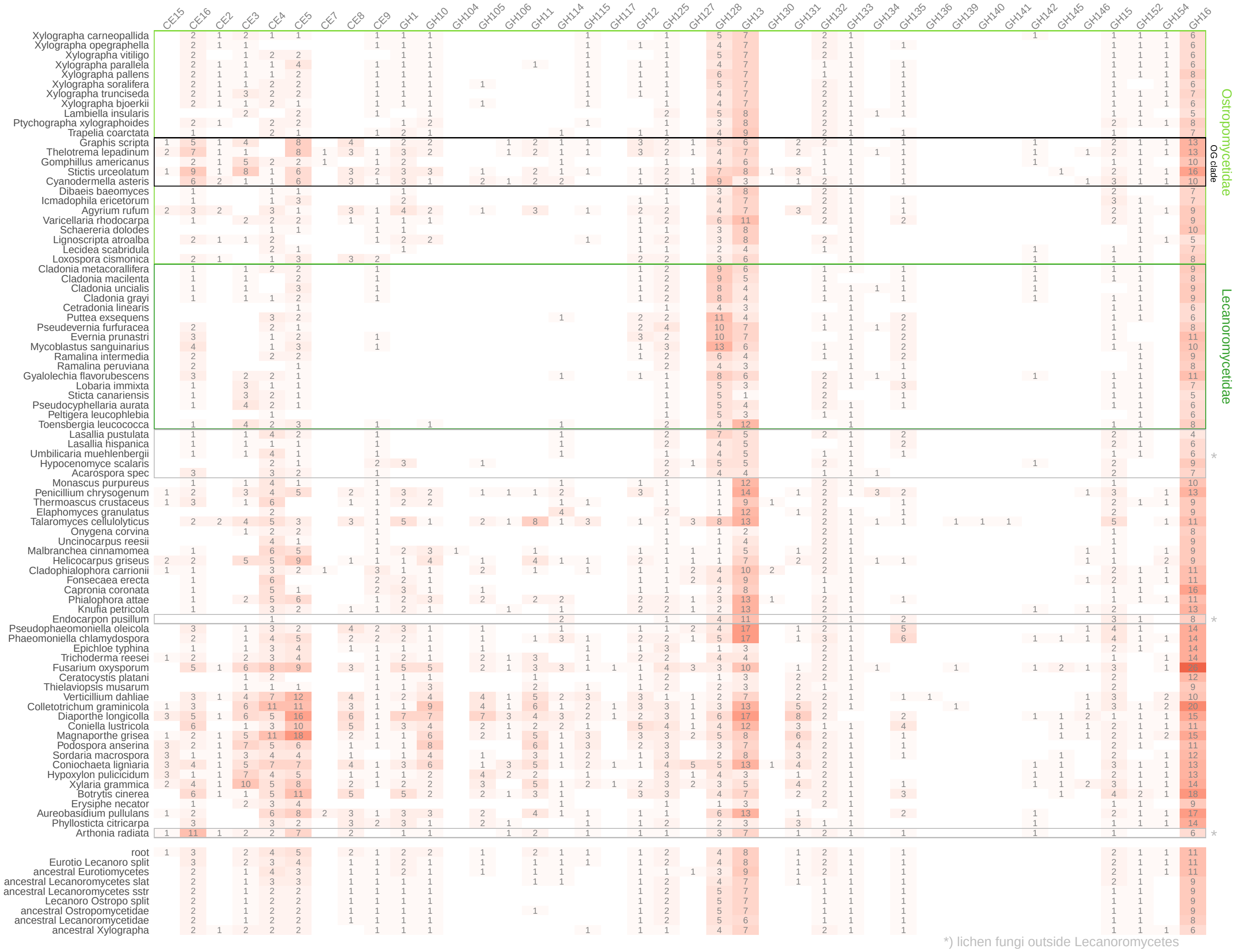

(b) part 2 of 5

Supplementary Figure 11: Heatmap of gene numbers of all CAZyme families recovered in 83 genomes analyzed in this study. Number is the bottom nine rows refer to reconstructed values at ancestral nodes according to Figure 1. Darker colors indicate higher number of genes.

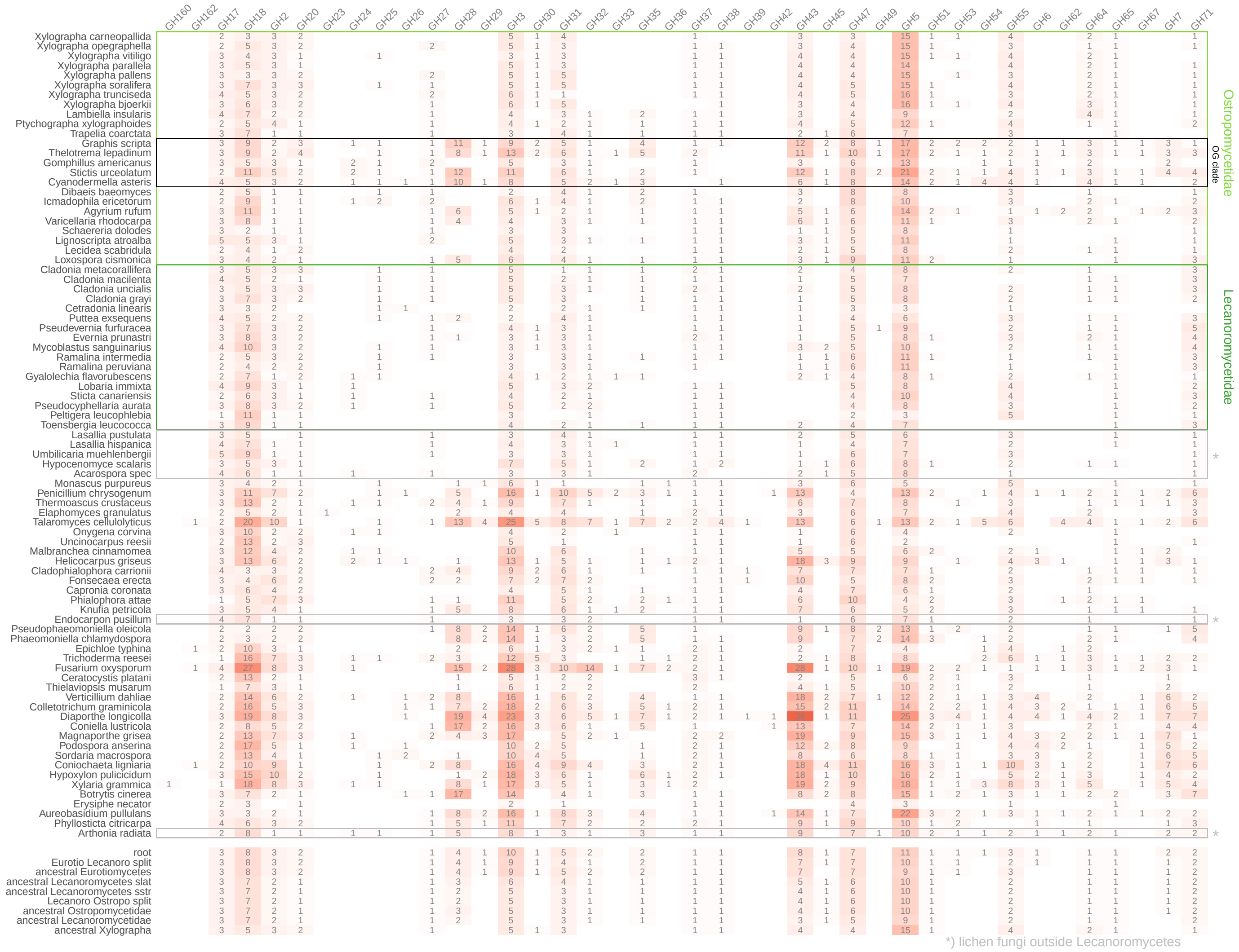

(c) part 3 of 5

Supplementary Figure 11: Heatmap of gene numbers of all CAZyme families recovered in 83 genomes analyzed in this study and for ancestral nodes according to Figure 1. Darker colors indicate higher number of genes.

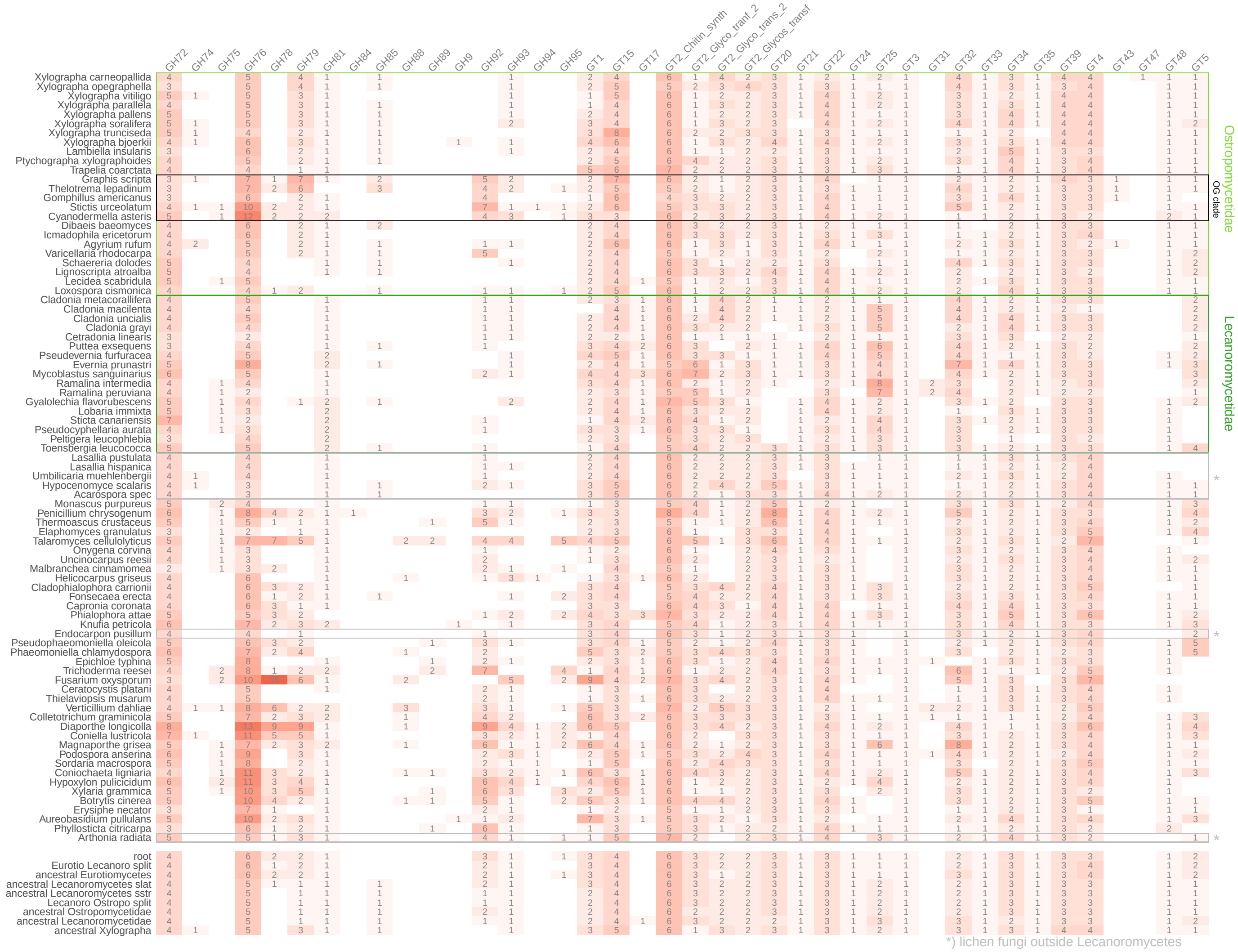

(d) part 4 of 5

Supplementary Figure 11: Heatmap of gene numbers of all CAZyme families recovered in 83 genomes analyzed in this study and for ancestral nodes according to Figure 1. Darker colors indicate more found genes.

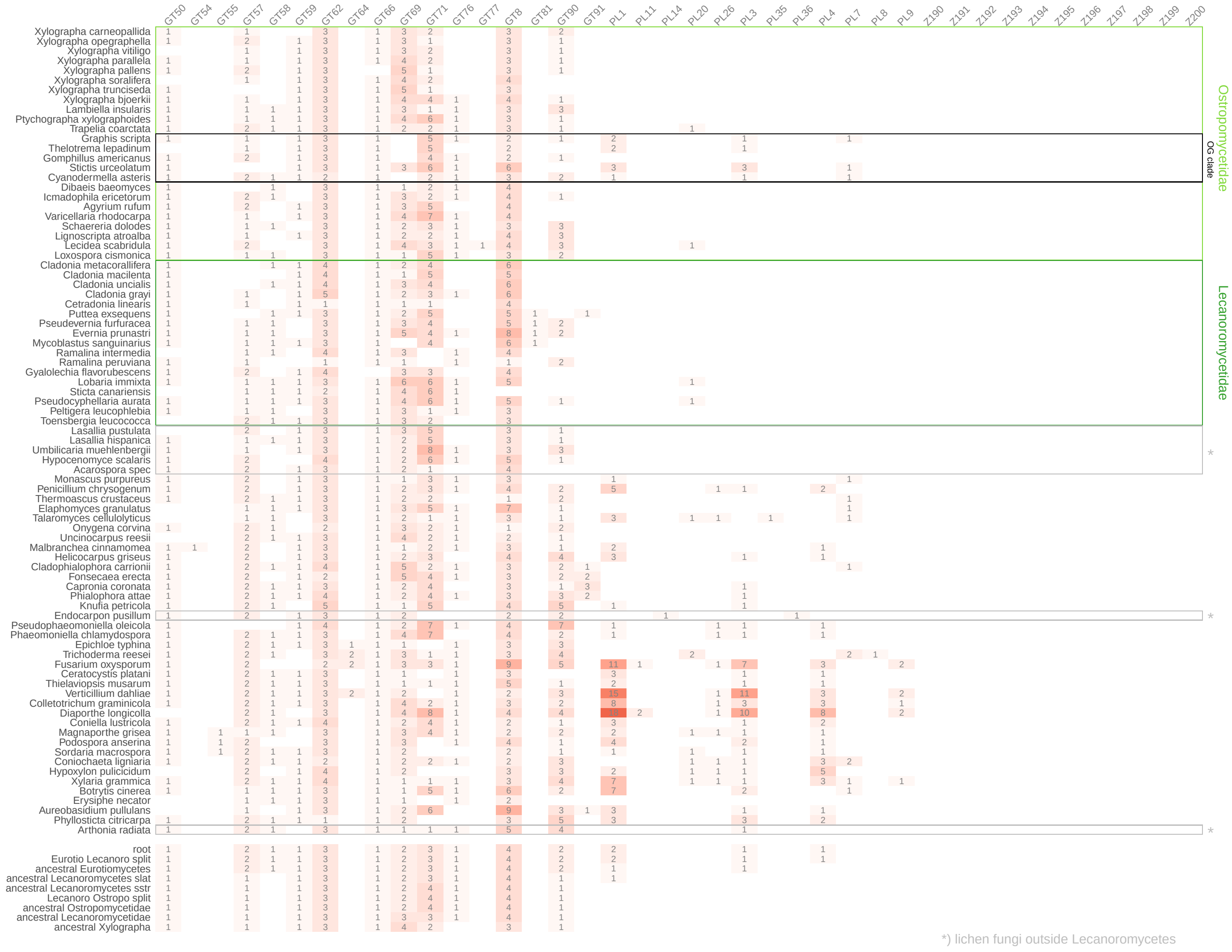

(e) part 5 of 5

Supplementary Figure 11: Heatmap of gene numbers of all CAZyme families recovered in 83 genomes analyzed in this study and for ancestral nodes according to Figure 1. Darker colors indicate higher number of genes.

Supplementary Figure 12: Overview of gene family expansion analyses results

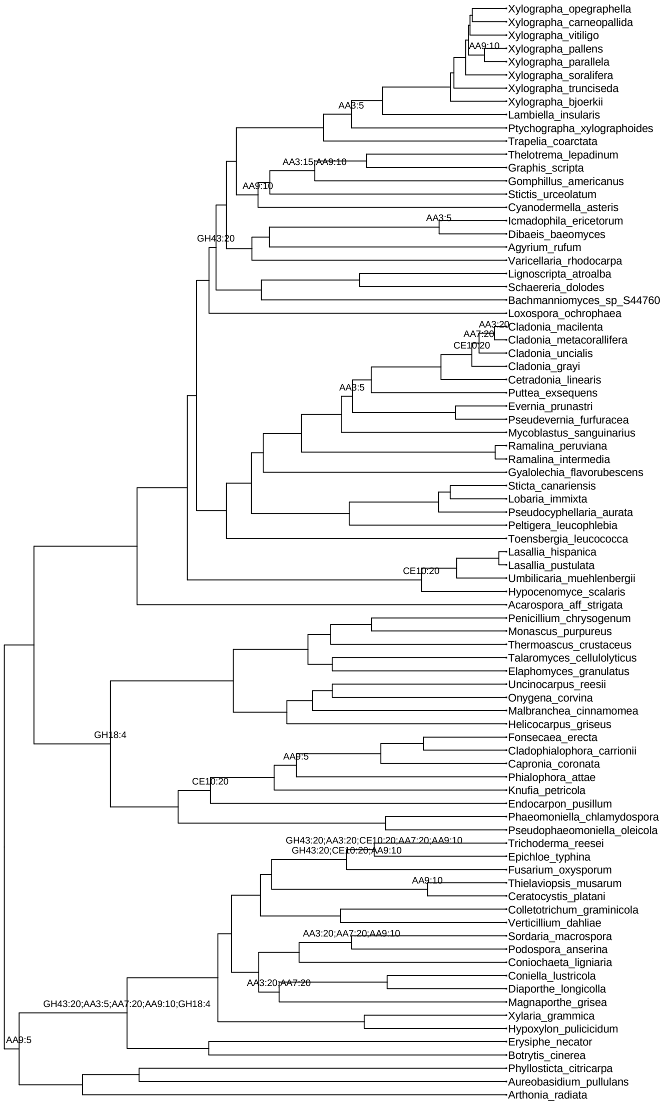

Supplementary Figure 12: This tree gives a summary of our gene family expansion analyses using CAFE5. Indicated at nodes are signficantly expanded gene families and the number of independet runs it was found to be expanded, seperated by a colon. This data was used to create Figure 1.

Supplementary Figure 13: Tree of putative invertases in GH32

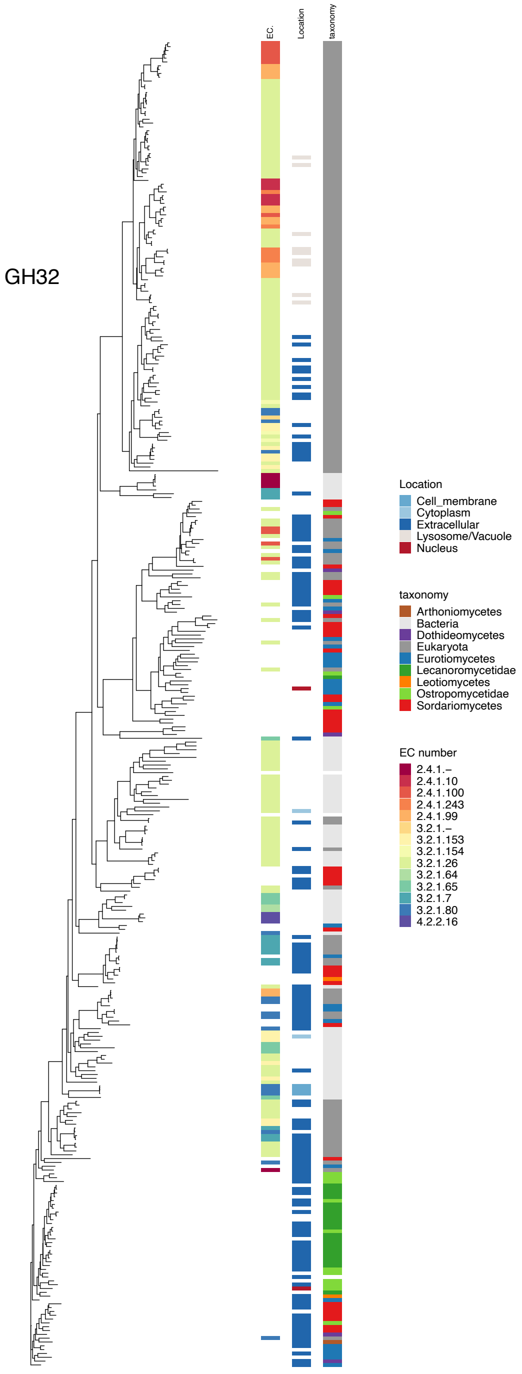

Supplementary Figure 13: Maximum-likelihood gene tree of the Glycoside Hydrolase family 32 containing invertases (EC 3.1.2.26). It includes all experimentally characterized GH32 sequences from cazy.org as well as all sequences with GH32 annotations from 83 genomes.

Supplementary Figures 14-61: Gene trees of CAZyme families generated with Saccharis

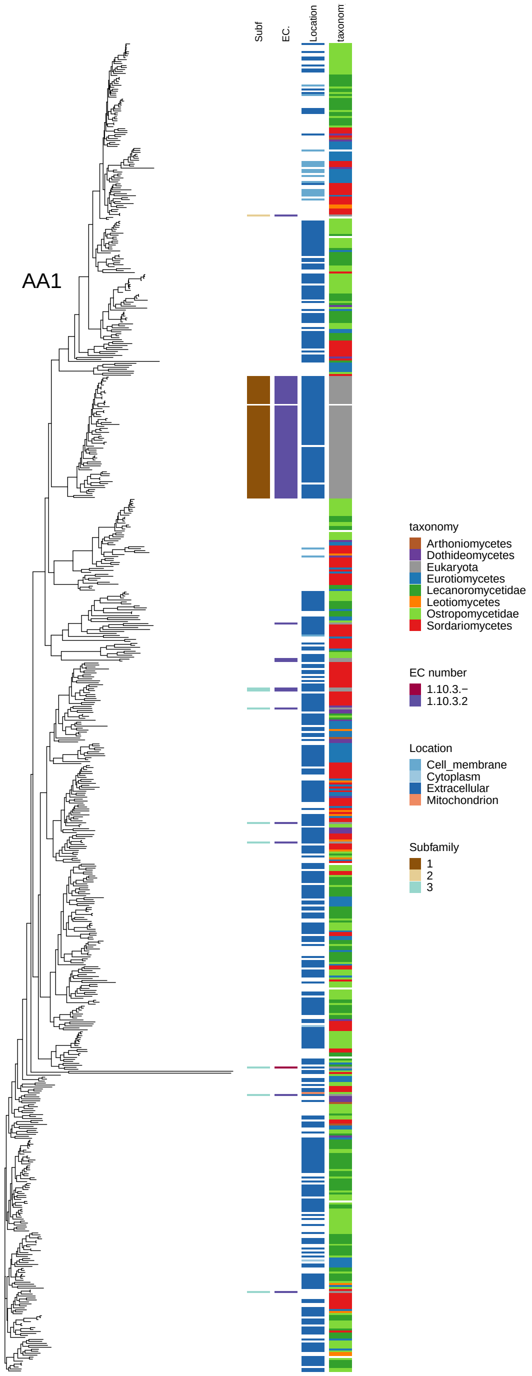

Supplementary Figure 14: Maximum-likelihood gene tree of the CAZyme family AA1. It includes all experimentally characterized sequences for that family downloaded from cazy.org as well as all sequences with AA1 annotations for in the 83 analyzed genomes including additional information where available.

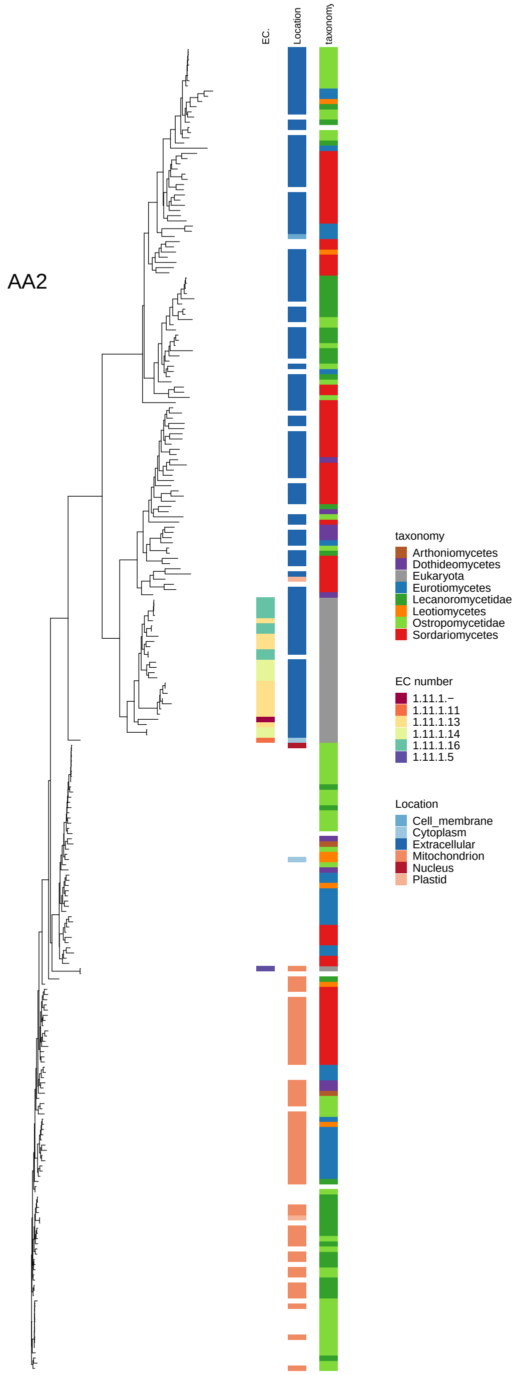

Supplementary Figure 15: Maximum-likelihood gene tree of the CAZyme family AA2. It includes all experimentally characterized sequences for that family downloaded from cazy.org as well as all sequences with AA2 annotations for in the 83 analyzed genomes including additional information where available.

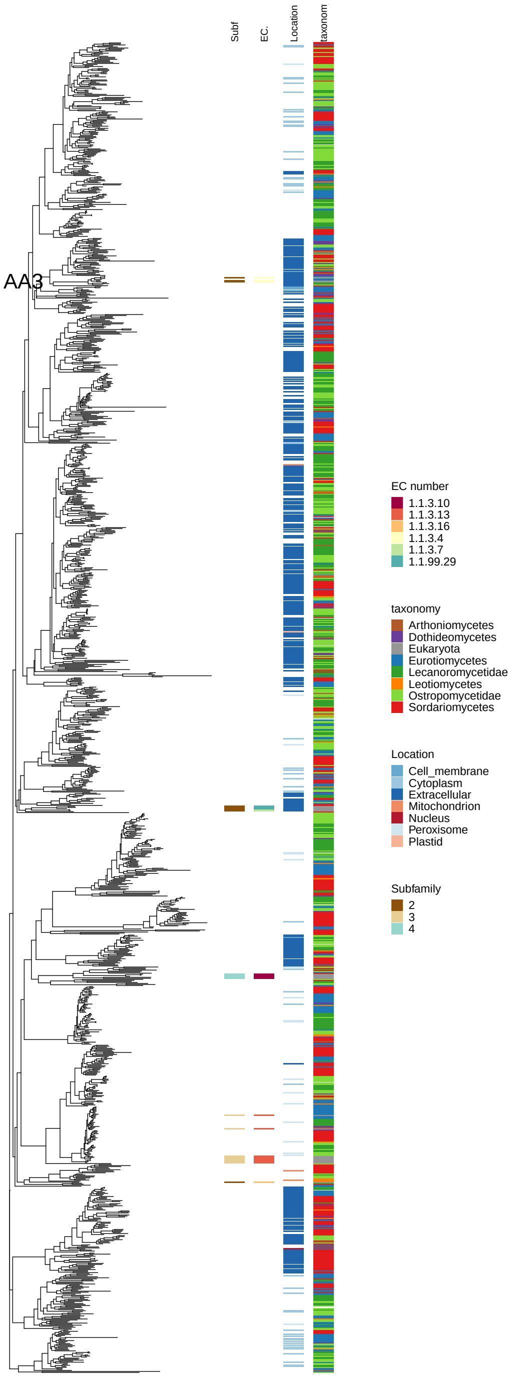

Supplementary Figure 16: Maximum-likelihood gene tree of the CAZyme family AA3. It includes all experimentally characterized sequences for that family downloaded from cazy.org as well as all sequences with AA3 annotations for in the 83 analyzed genomes including additional information where available.

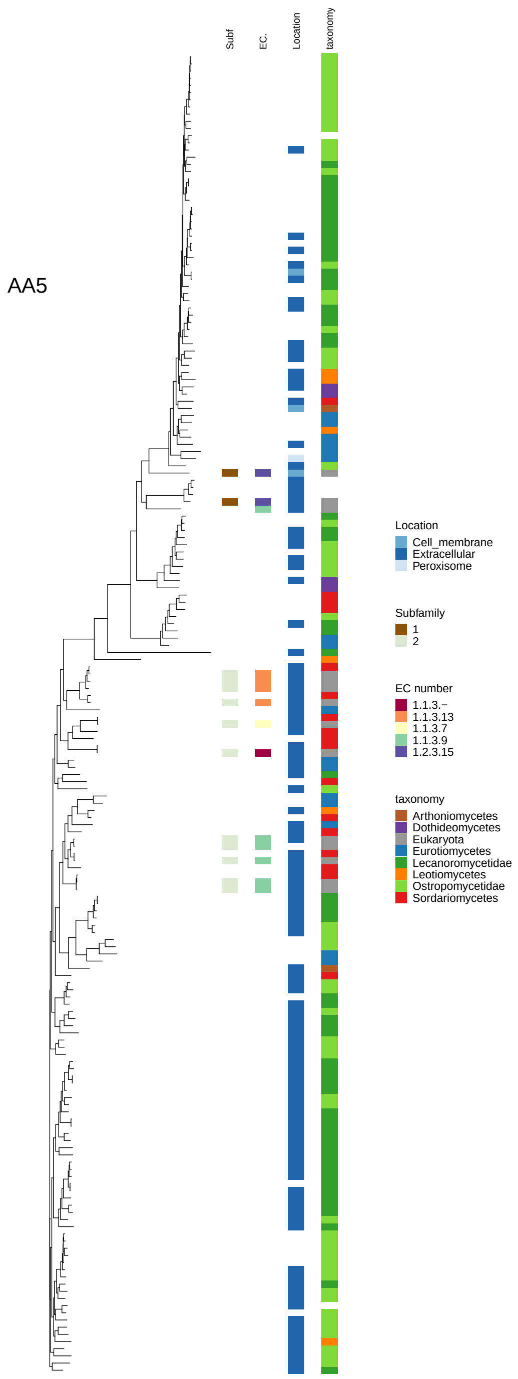

Supplementary Figure 17: Maximum-likelihood gene tree of the CAZyme family AA5. It includes all experimentally characterized sequences for that family downloaded from cazy.org as well as all sequences with AA5 annotations for in the 83 analyzed genomes including additional information where available.

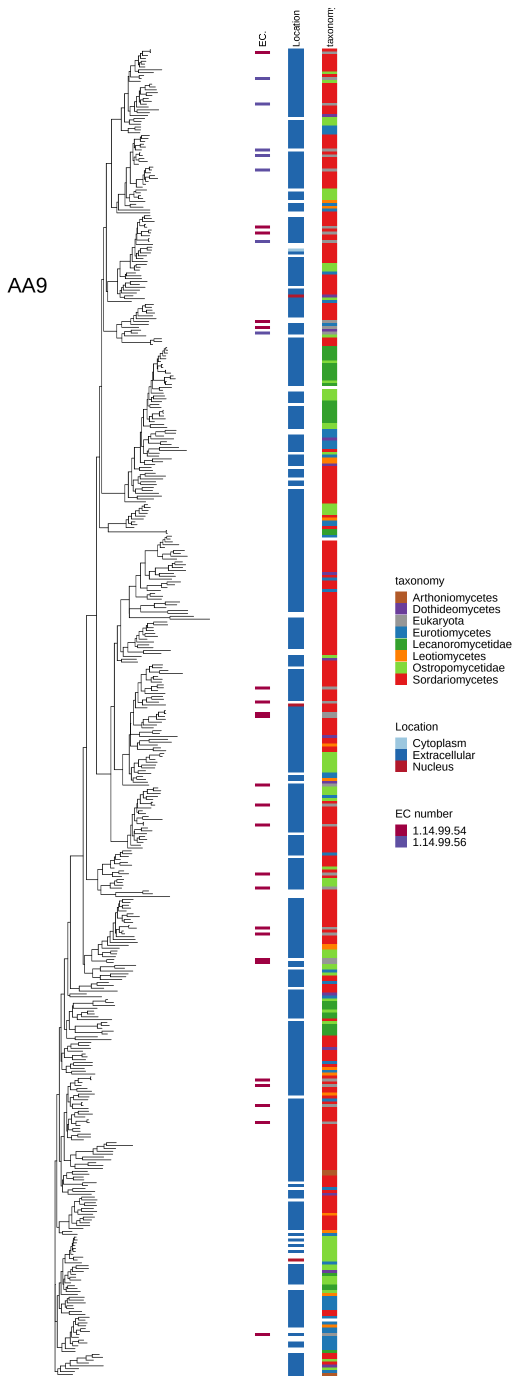

Supplementary Figure 18: Maximum-likelihood gene tree of the CAZyme family AA9. It includes all experimentally characterized sequences for that family downloaded from cazy.org as well as all sequences with AA9 annotations for in the 83 analyzed genomes including additional information where available.

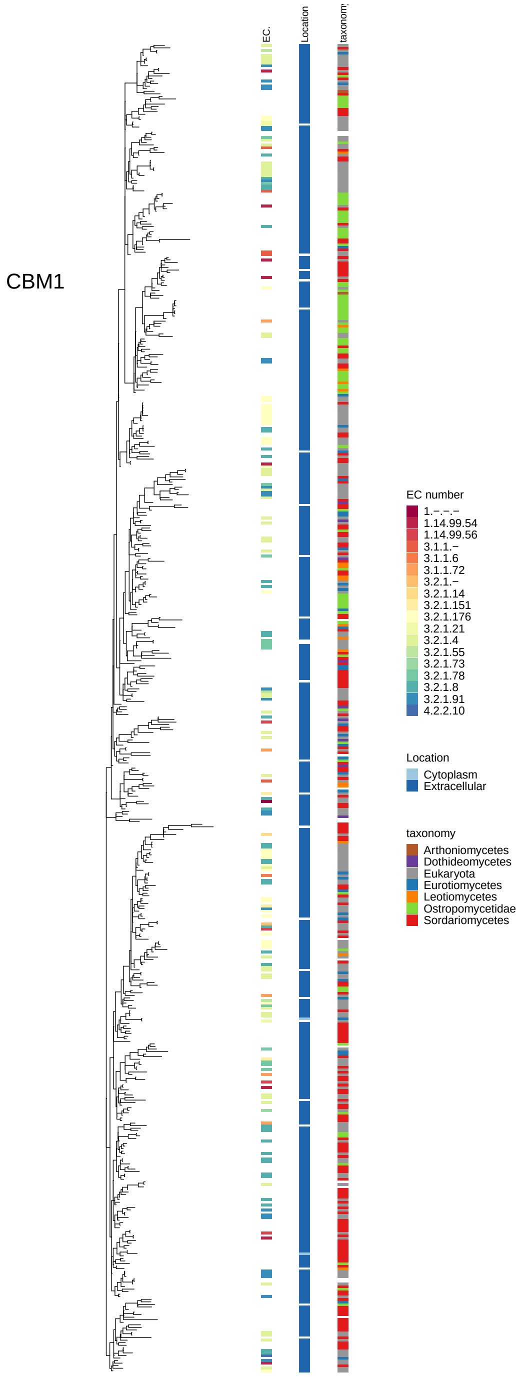

Supplementary Figure 19: Maximum-likelihood gene tree of the CAZyme family CBM1. It includes all experimentally characterized sequences for that family downloaded from cazy.org as well as all sequences with CBM1 annotations for in the 83 analyzed genomes including additional information where available.

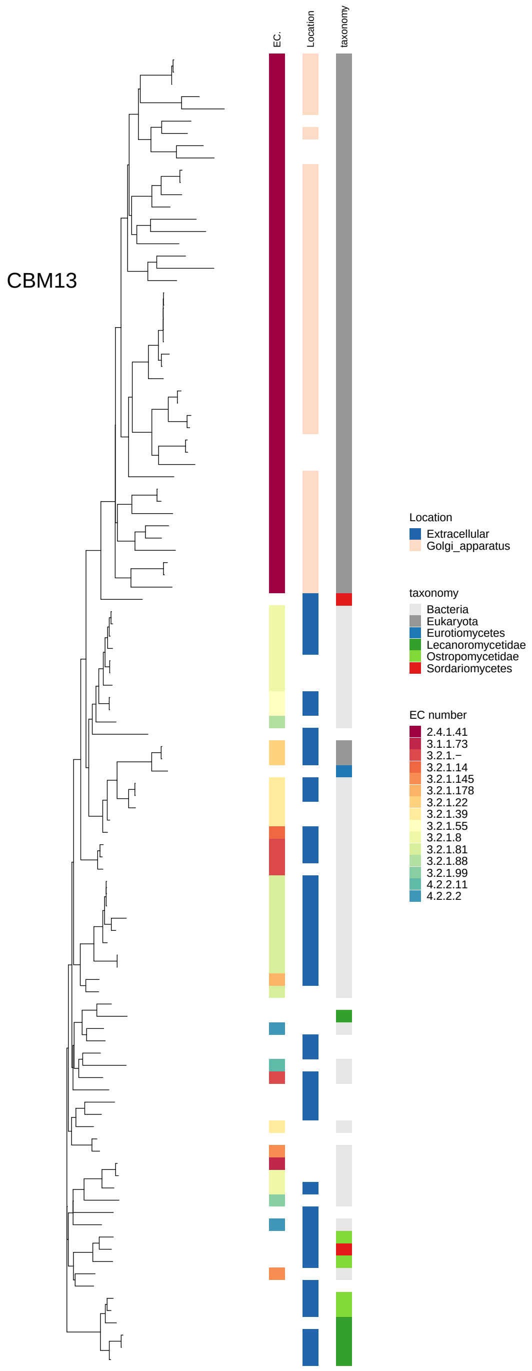

Supplementary Figure 20: Maximum-likelihood gene tree of the CAZyme family CBM13. It includes all experimentally characterized sequences for that family downloaded from cazy.org as well as all sequences with CBM13 annotations for in the 83 analyzed genomes including additional information where available.

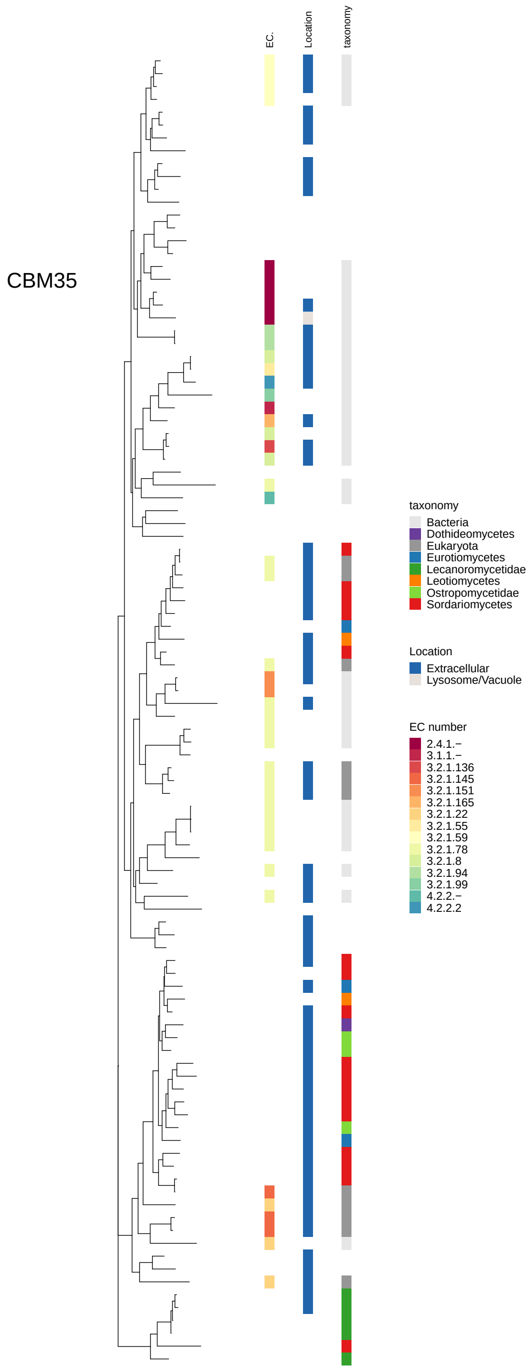

Supplementary Figure 21: Maximum-likelihood gene tree of the CAZyme family CBM35. It includes all experimentally characterized sequences for that family downloaded from cazy.org as well as all sequences with CBM35 annotations for in the 83 analyzed genomes including additional information where available.

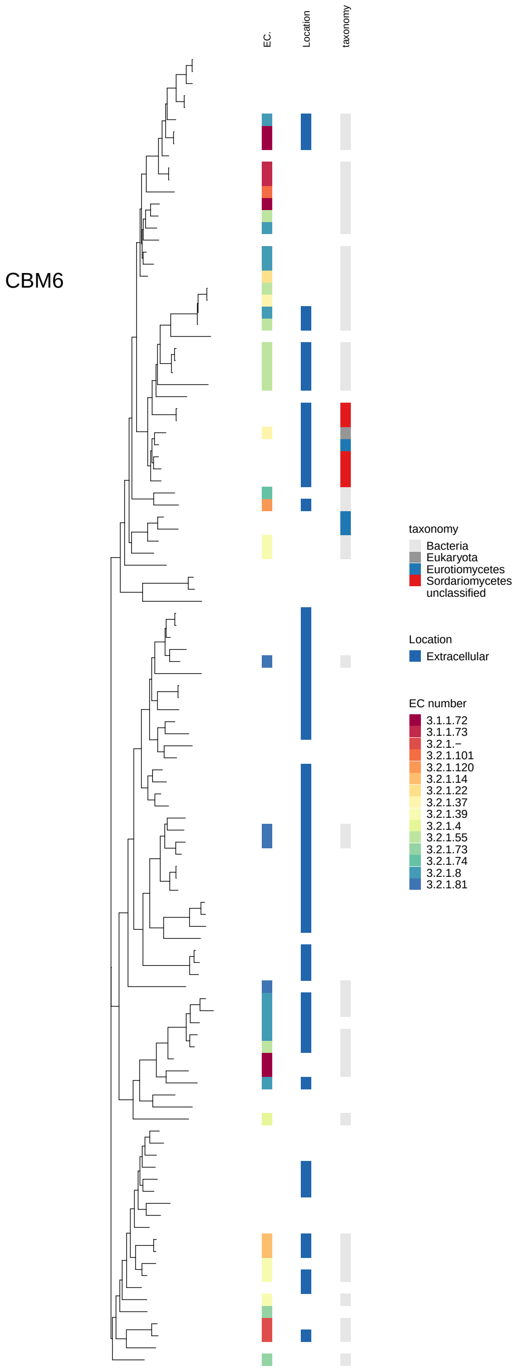

Supplementary Figure 22: Maximum-likelihood gene tree of the CAZyme family CBM6. It includes all experimentally characterized sequences for that family downloaded from cazy.org as well as all sequences with CBM6 annotations for in the 83 analyzed genomes including additional information where available.

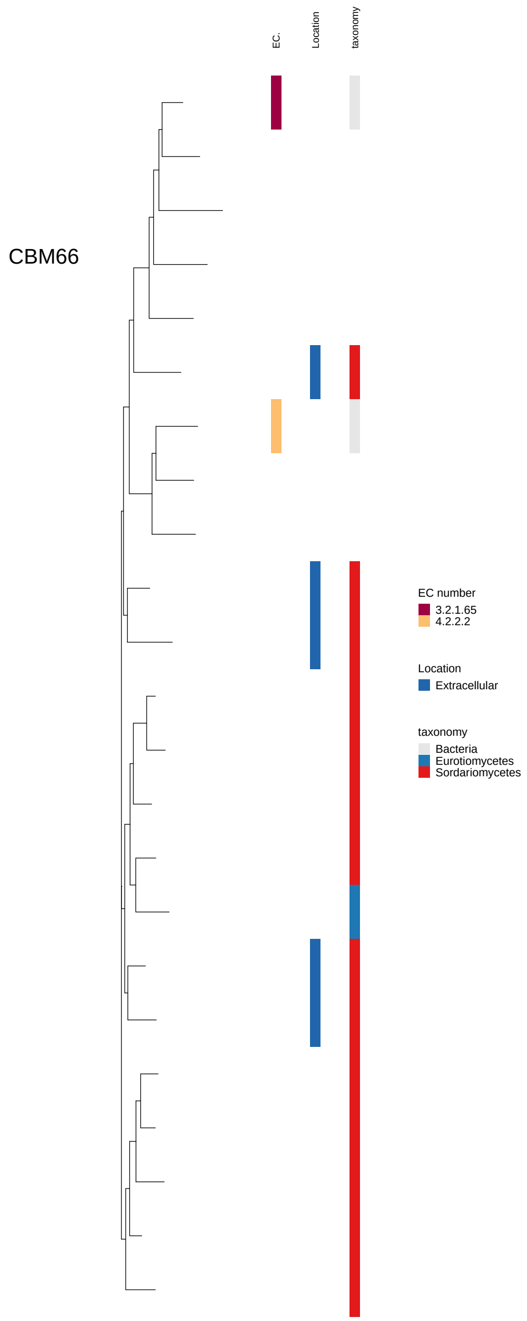

Supplementary Figure 23: Maximum-likelihood gene tree of the CAZyme family CBM66. It includes all experimentally characterized sequences for that family downloaded from cazy.org as well as all sequences with CBM66 annotations for in the 83 analyzed genomes including additional information where available.

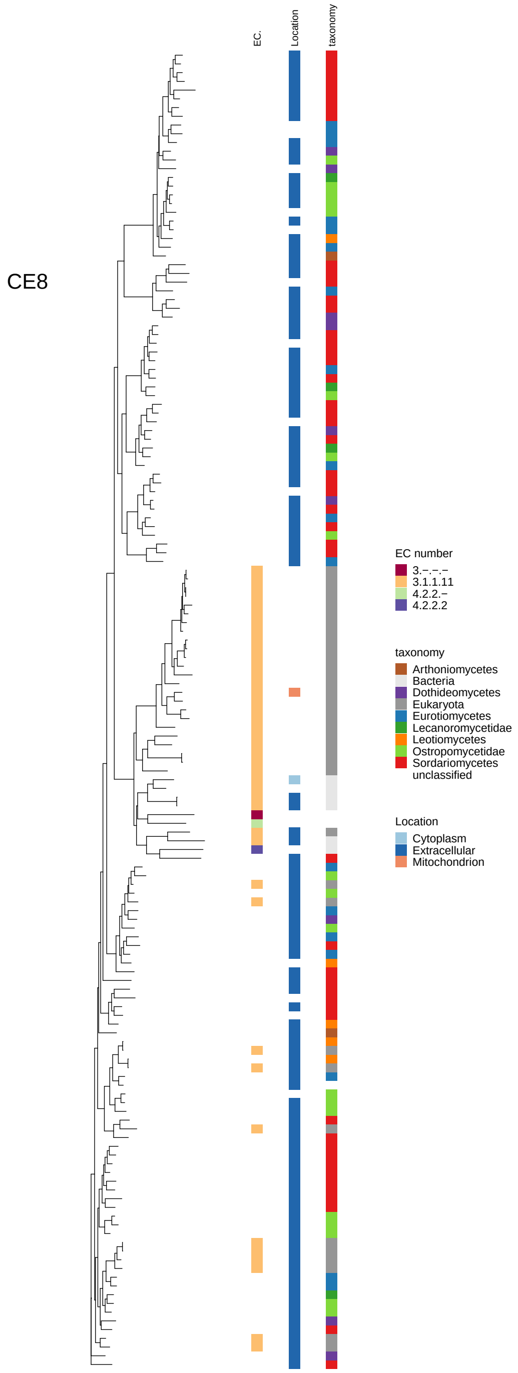

Supplementary Figure 24: Maximum-likelihood gene tree of the CAZyme family CE8. It includes all experimentally characterized sequences for that family downloaded from cazy.org as well as all sequences with CE8 annotations for in the 83 analyzed genomes including additional information where available.

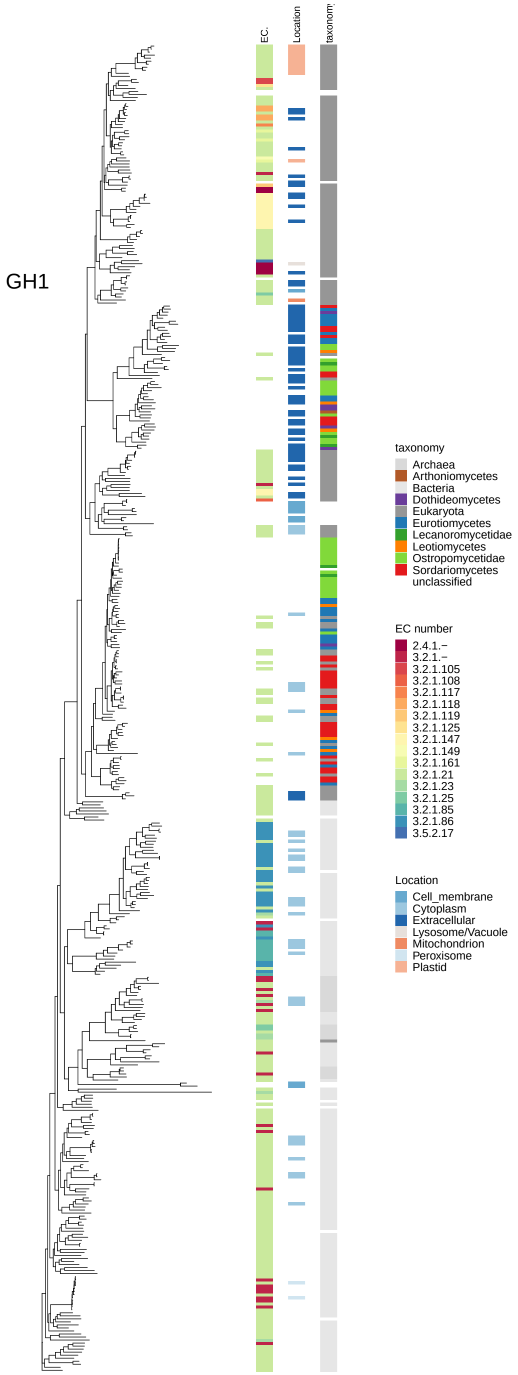

Supplementary Figure 25: Maximum-likelihood gene tree of the CAZyme family GH1. It includes all experimentally characterized sequences for that family downloaded from cazy.org as well as all sequences with GH1 annotations for in the 83 analyzed genomes including additional information where available.

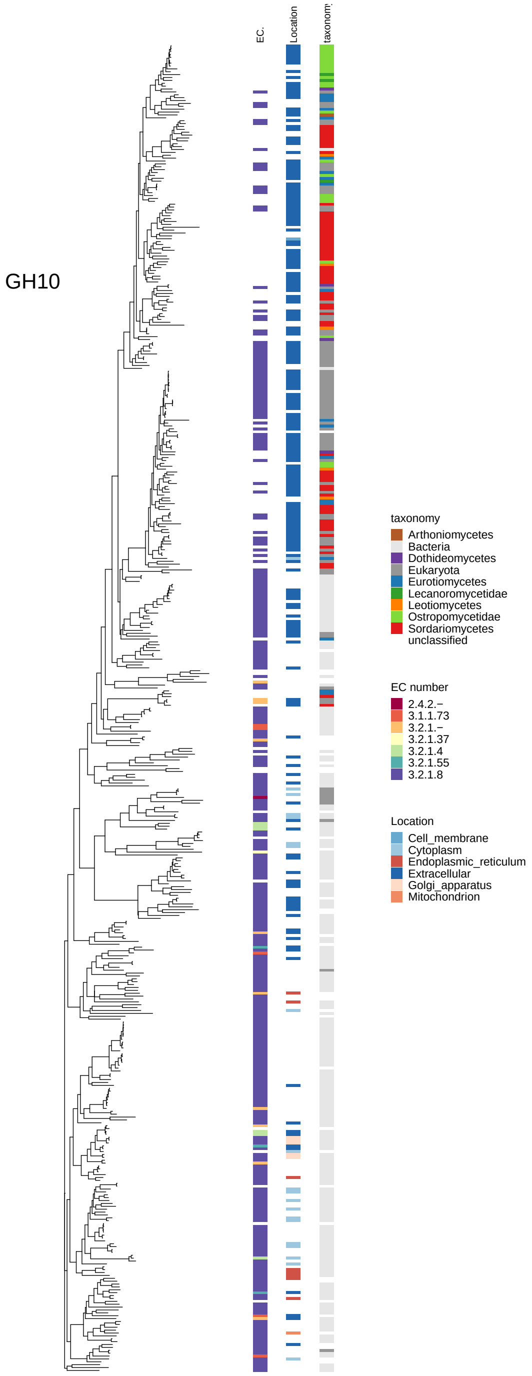

Supplementary Figure 26: Maximum-likelihood gene tree of the CAZyme family GH10. It includes all experimentally characterized sequences for that family downloaded from cazy.org as well as all sequences with GH10 annotations for in the 83 analyzed genomes including additional information where available.

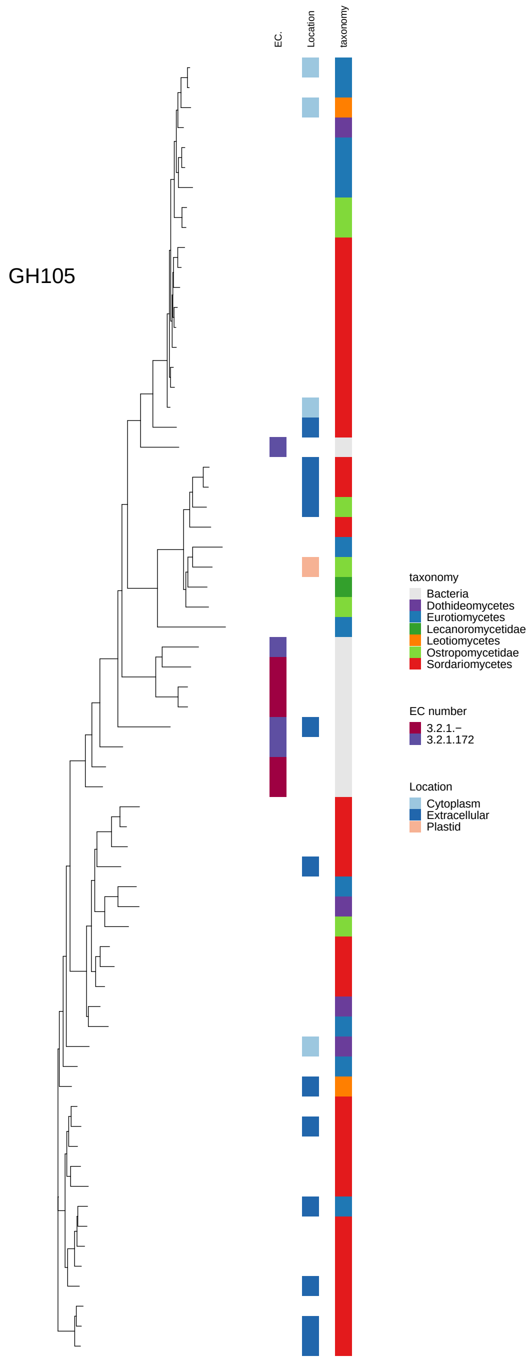

Supplementary Figure 27: Maximum-likelihood gene tree of the CAZyme family GH105. It includes all experimentally characterized sequences for that family downloaded from cazy.org as well as all sequences with GH105 annotations for in the 83 analyzed genomes including additional information where available.

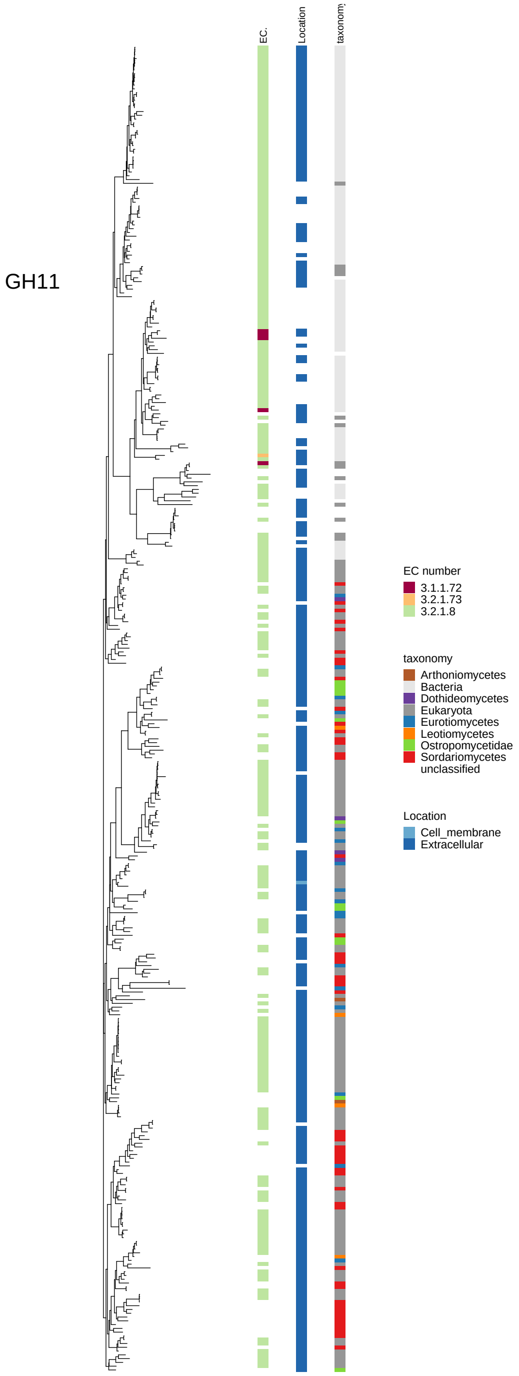

Supplementary Figure 28: Maximum-likelihood gene tree of the CAZyme family GH11. It includes all experimentally characterized sequences for that family downloaded from cazy.org as well as all sequences with GH11 annotations for in the 83 analyzed genomes including additional information where available.

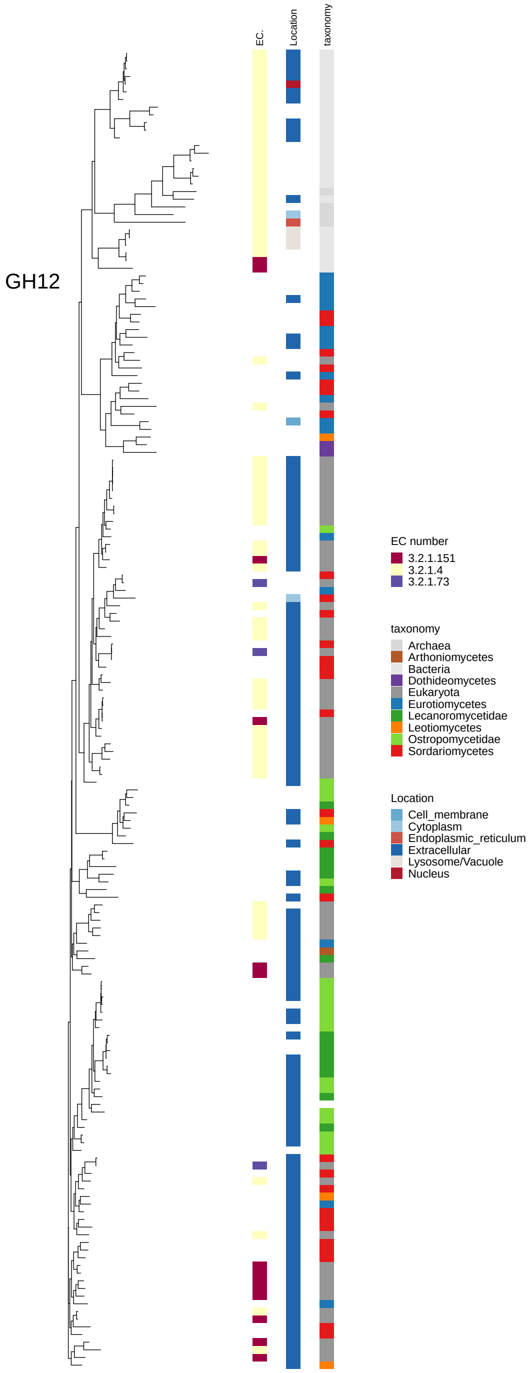

Supplementary Figure 29: Maximum-likelihood gene tree of the CAZyme family GH12. It includes all experimentally characterized sequences for that family downloaded from cazy.org as well as all sequences with GH12 annotations for in the 83 analyzed genomes including additional information where available.

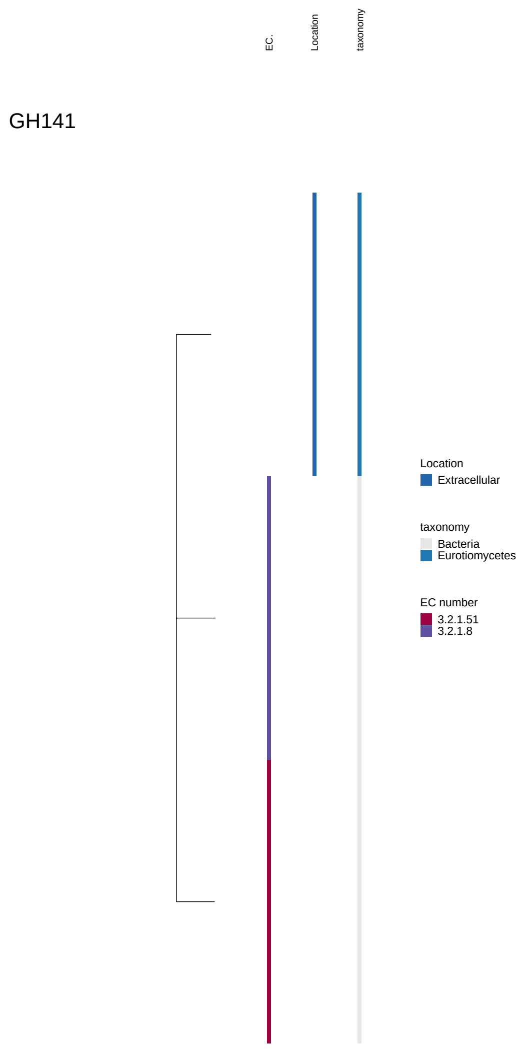

Supplementary Figure 30: Maximum-likelihood gene tree of the CAZyme family GH141. It includes all experimentally characterized sequences for that family downloaded from cazy.org as well as all sequences with GH141 annotations for in the 83 analyzed genomes including additional information where available.

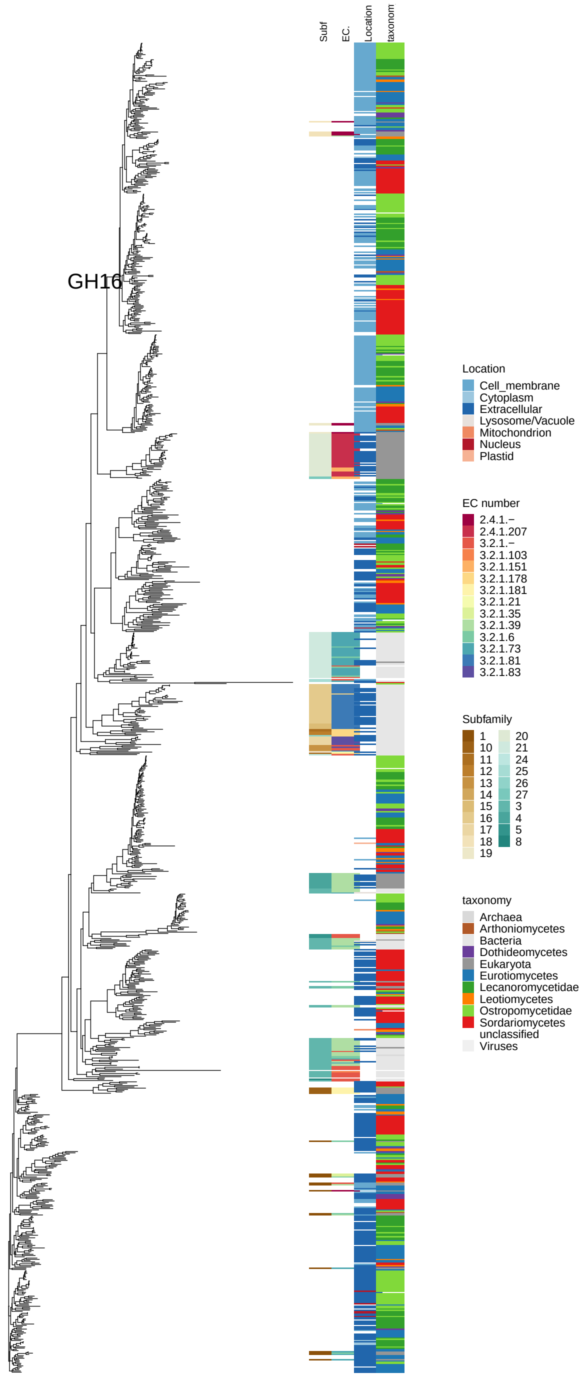

Supplementary Figure 31: Maximum-likelihood gene tree of the CAZyme family GH16. It includes all experimentally characterized sequences for that family downloaded from cazy.org as well as all sequences with GH16 annotations for in the 83 analyzed genomes including additional information where available.

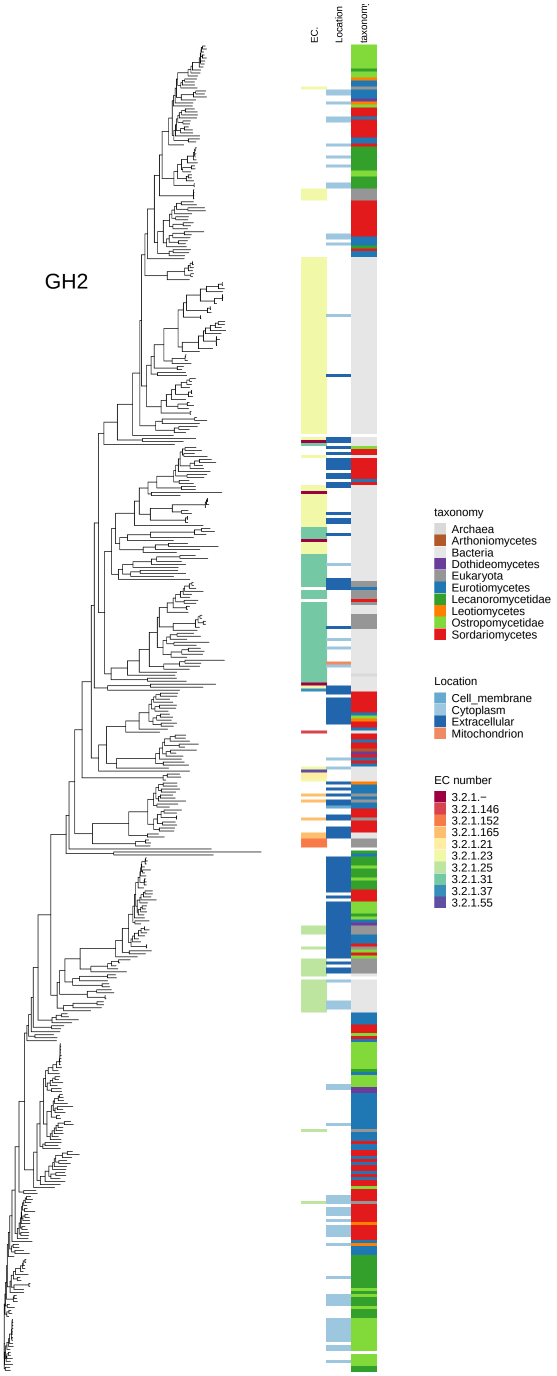

Supplementary Figure 32: Maximum-likelihood gene tree of the CAZyme family GH2. It includes all experimentally characterized sequences for that family downloaded from cazy.org as well as all sequences with GH2 annotations for in the 83 analyzed genomes including additional information where available.

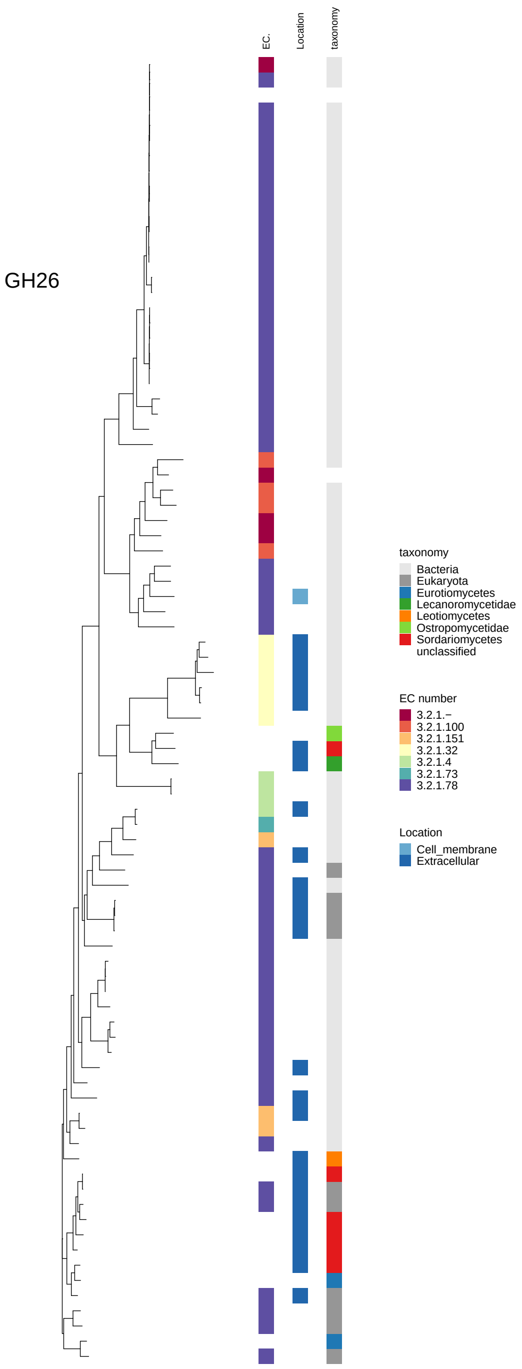

Supplementary Figure 33: Maximum-likelihood gene tree of the CAZyme family GH26. It includes all experimentally characterized sequences for that family downloaded from cazy.org as well as all sequences with GH26 annotations for in the 83 analyzed genomes including additional information where available.

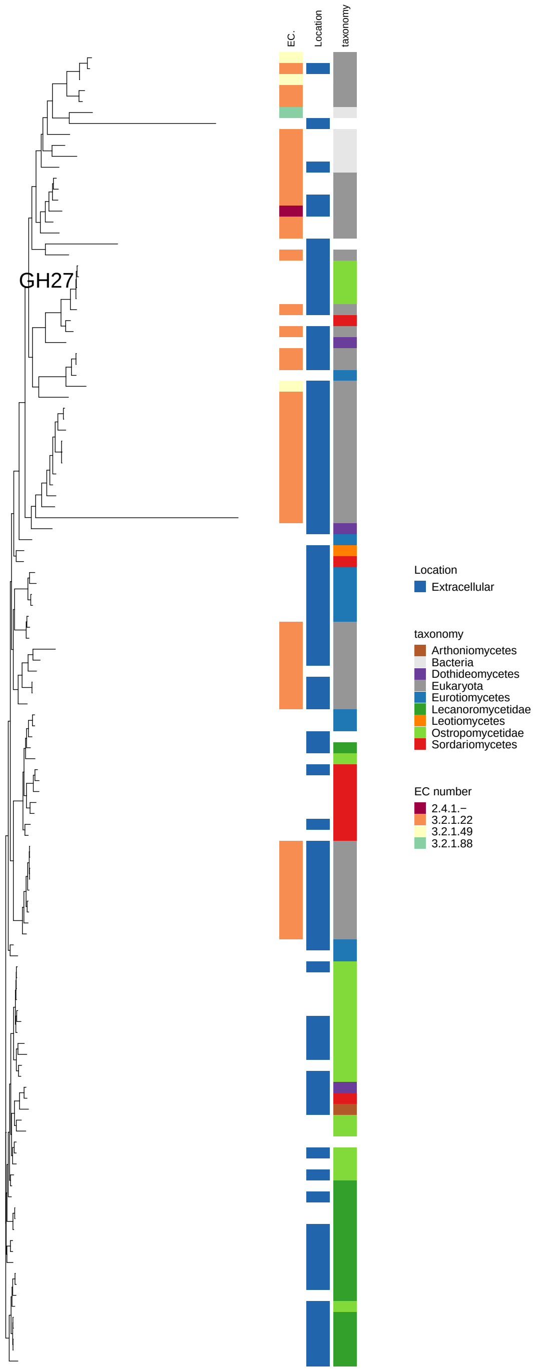

Supplementary Figure 34: Maximum-likelihood gene tree of the CAZyme family GH27. It includes all experimentally characterized sequences for that family downloaded from cazy.org as well as all sequences with GH27 annotations for in the 83 analyzed genomes including additional information where available.

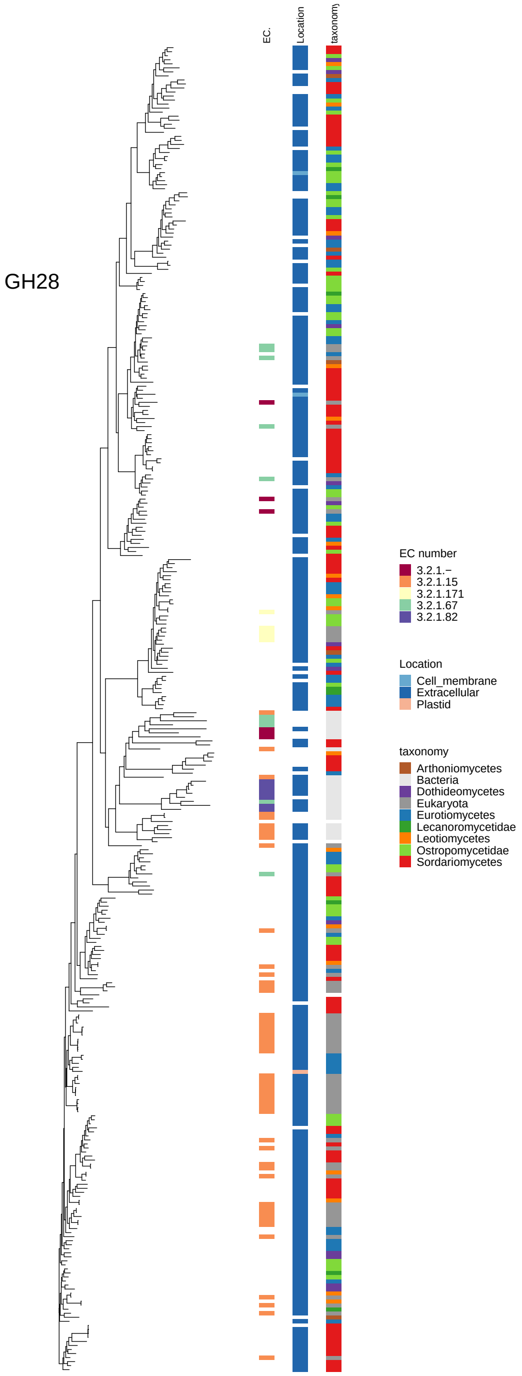

Supplementary Figure 35: Maximum-likelihood gene tree of the CAZyme family GH28. It includes all experimentally characterized sequences for that family downloaded from cazy.org as well as all sequences with GH28 annotations for in the 83 analyzed genomes including additional information where available.

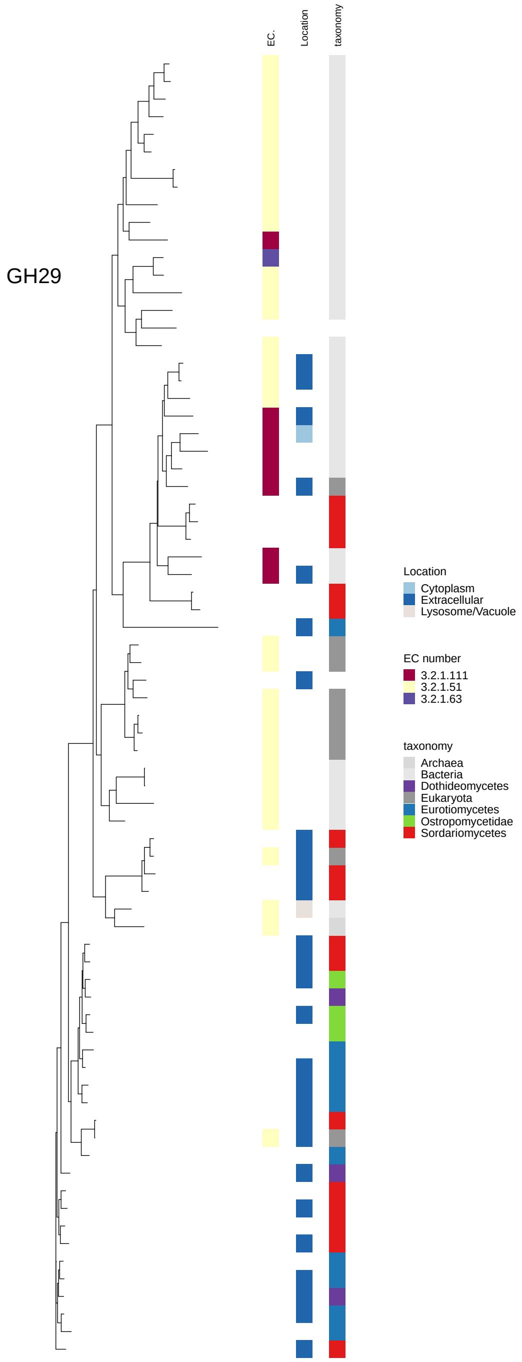

Supplementary Figure 36: Maximum-likelihood gene tree of the CAZyme family GH29. It includes all experimentally characterized sequences for that family downloaded from cazy.org as well as all sequences with GH29 annotations for in the 83 analyzed genomes including additional information where available.

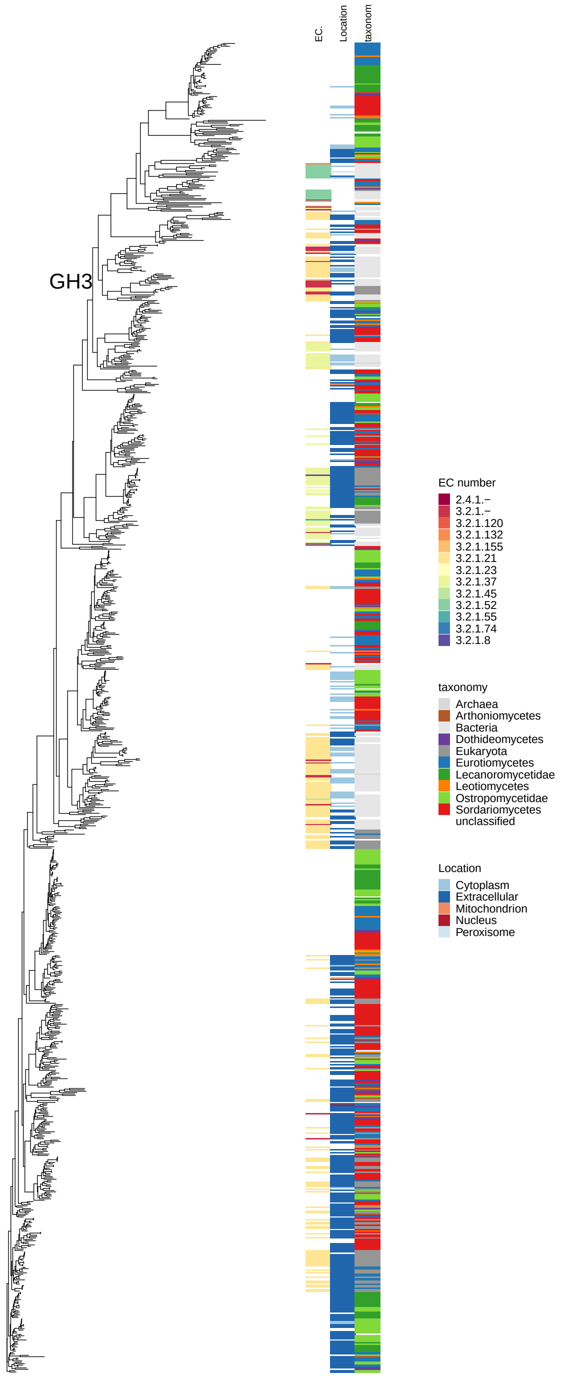

Supplementary Figure 37: Maximum-likelihood gene tree of the CAZyme family GH3. It includes all experimentally characterized sequences for that family downloaded from cazy.org as well as all sequences with GH3 annotations for in the 83 analyzed genomes including additional information where available.

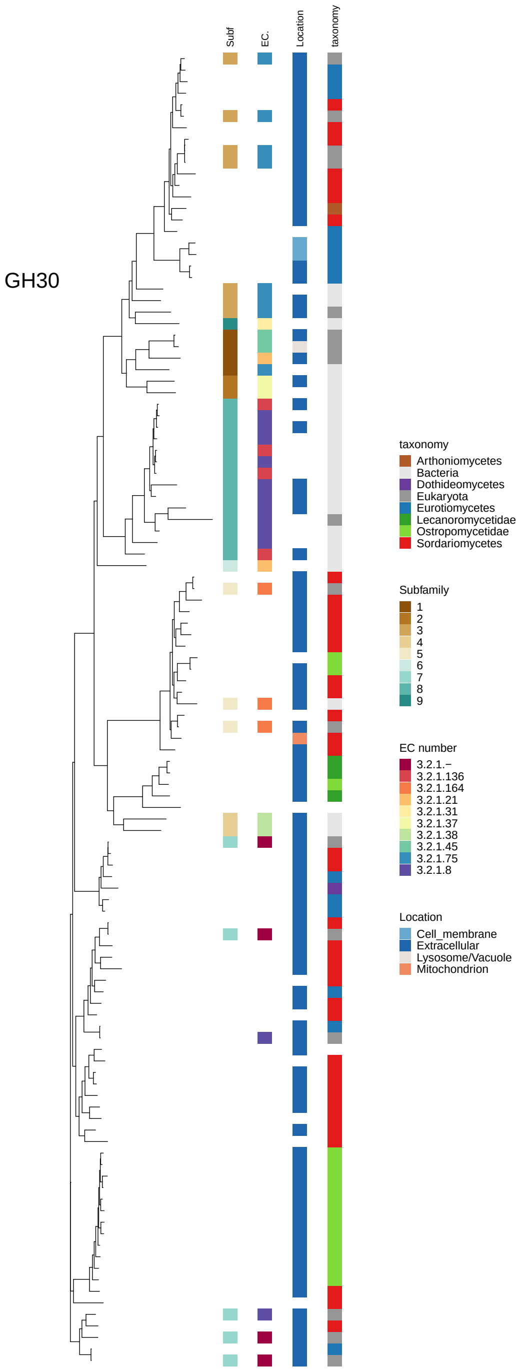

Supplementary Figure 38: Maximum-likelihood gene tree of the CAZyme family GH30. It includes all experimentally characterized sequences for that family downloaded from cazy.org as well as all sequences with GH30 annotations for in the 83 analyzed genomes including additional information where available.

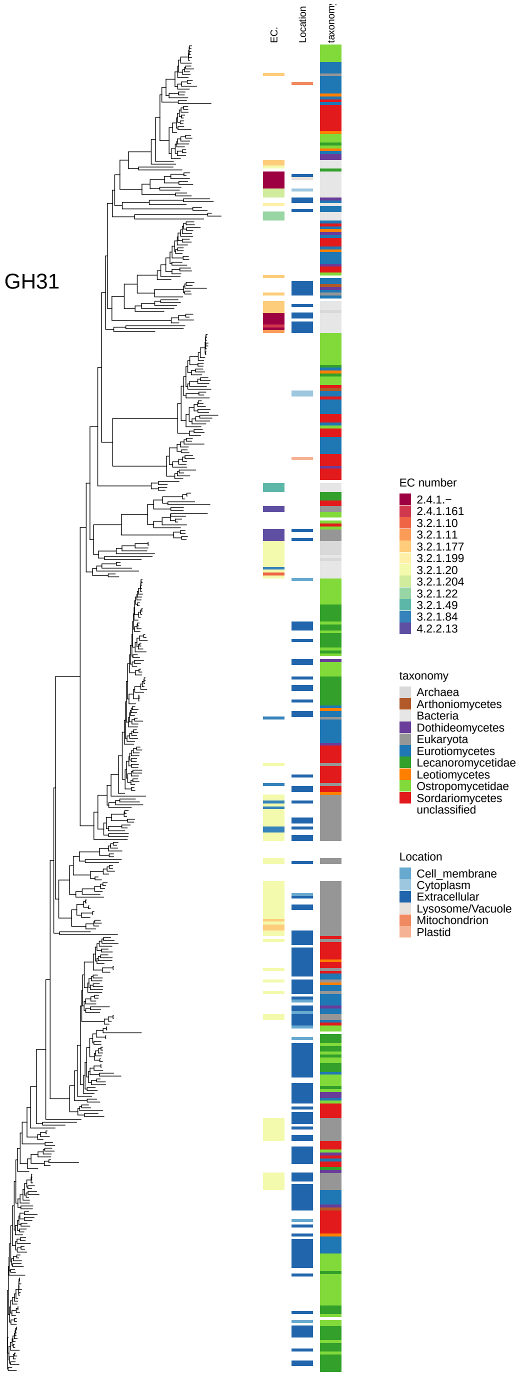

Supplementary Figure 39: Maximum-likelihood gene tree of the CAZyme family GH31. It includes all experimentally characterized sequences for that family downloaded from cazy.org as well as all sequences with GH31 annotations for in the 83 analyzed genomes including additional information where available.

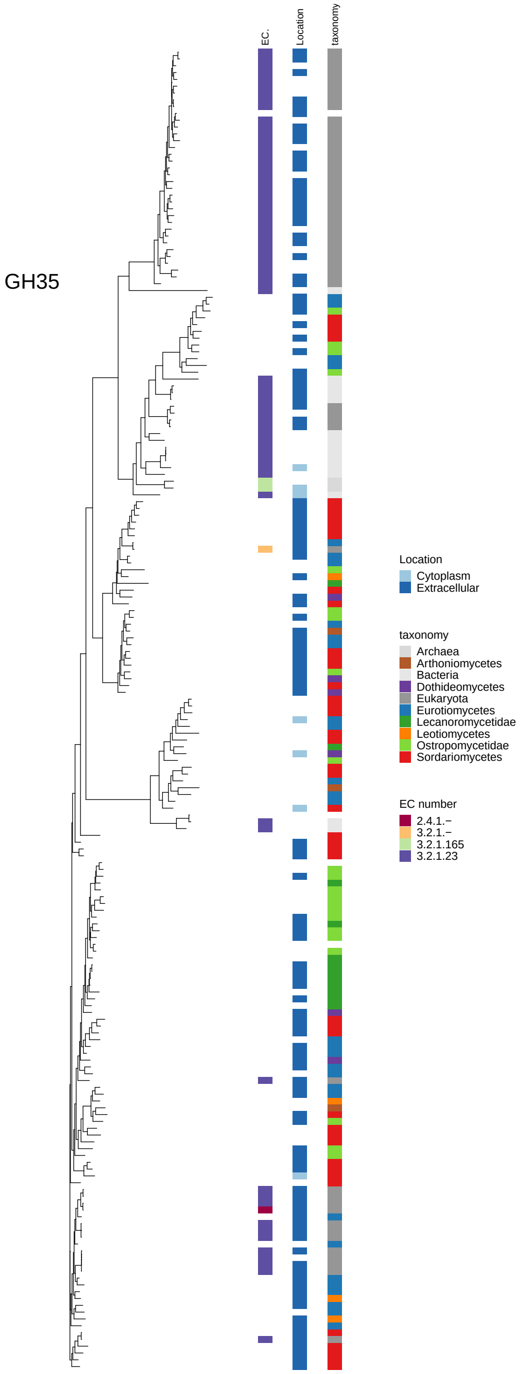

Supplementary Figure 40: Maximum-likelihood gene tree of the CAZyme family GH35. It includes all experimentally characterized sequences for that family downloaded from cazy.org as well as all sequences with GH35 annotations for in the 83 analyzed genomes including additional information where available.

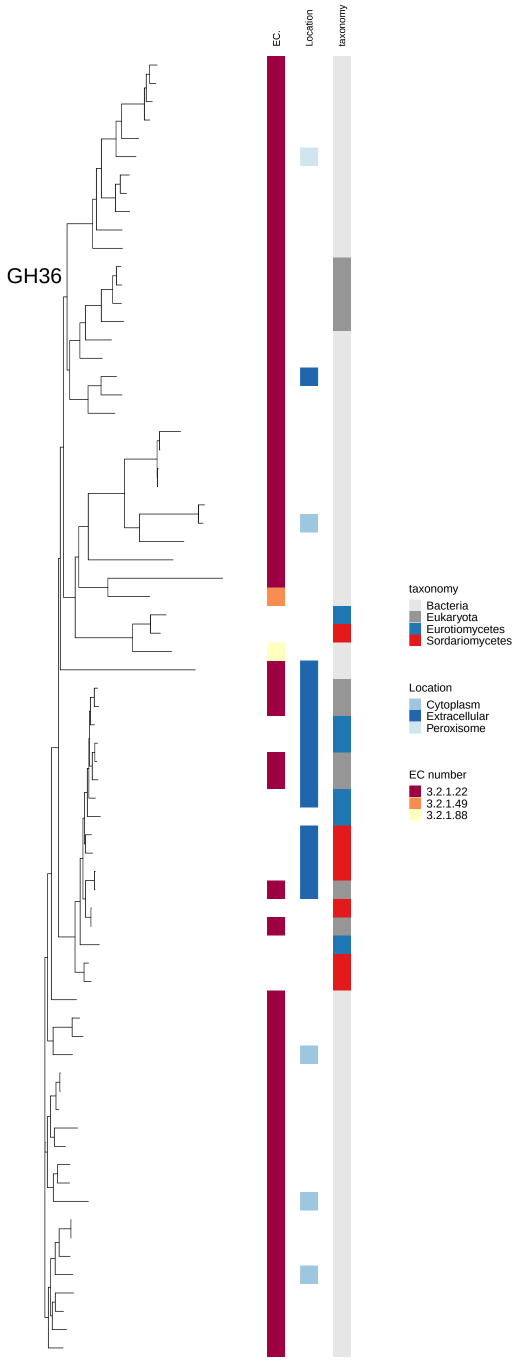

Supplementary Figure 41: Maximum-likelihood gene tree of the CAZyme family GH36. It includes all experimentally characterized sequences for that family downloaded from cazy.org as well as all sequences with GH36 annotations for in the 83 analyzed genomes including additional information where available.

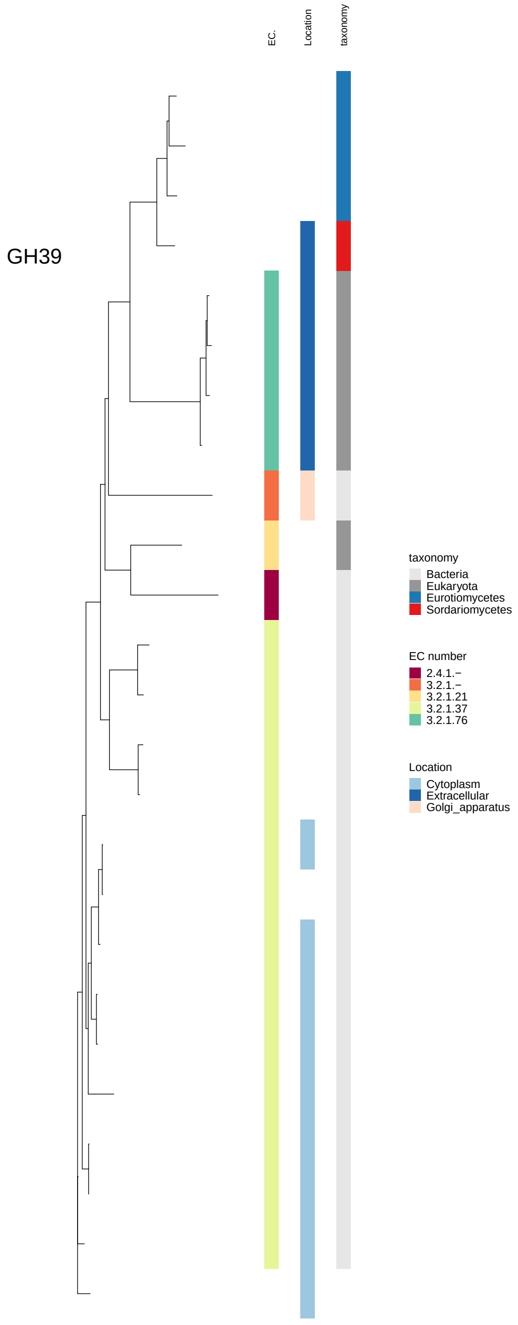

Supplementary Figure 42: Maximum-likelihood gene tree of the CAZyme family GH39. It includes all experimentally characterized sequences for that family downloaded from cazy.org as well as all sequences with GH39 annotations for in the 83 analyzed genomes including additional information where available.

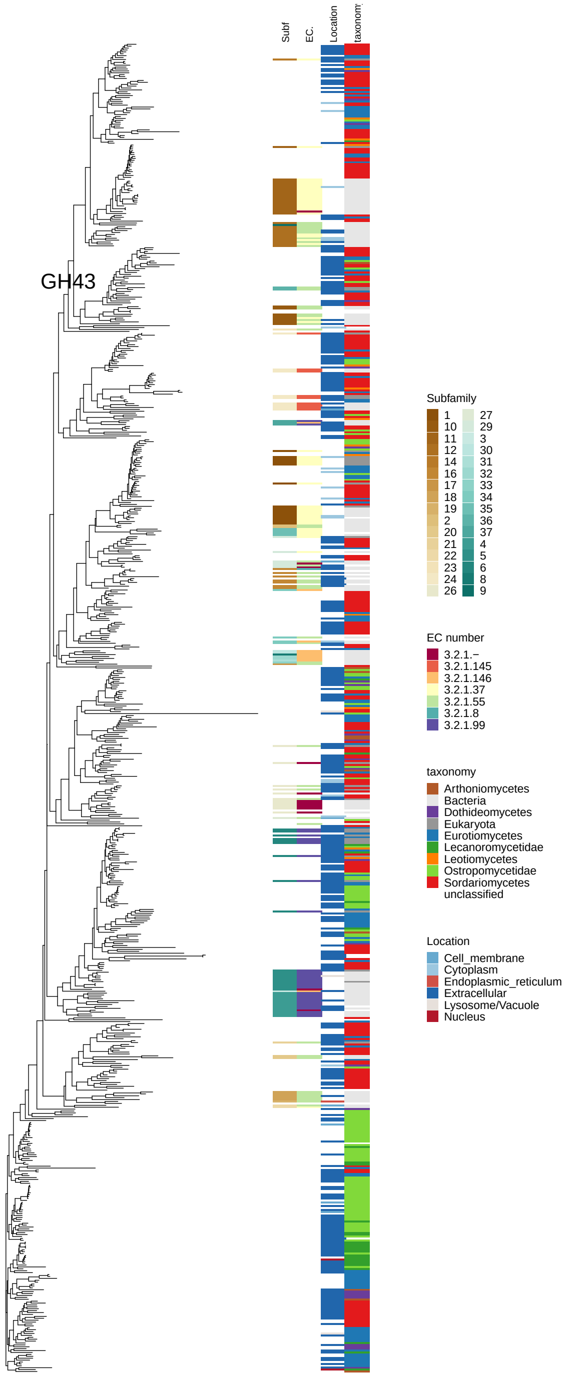

Supplementary Figure 43: Maximum-likelihood gene tree of the CAZyme family GH43. It includes all experimentally characterized sequences for that family downloaded from cazy.org as well as all sequences with GH43 annotations for in the 83 analyzed genomes including additional information where available.

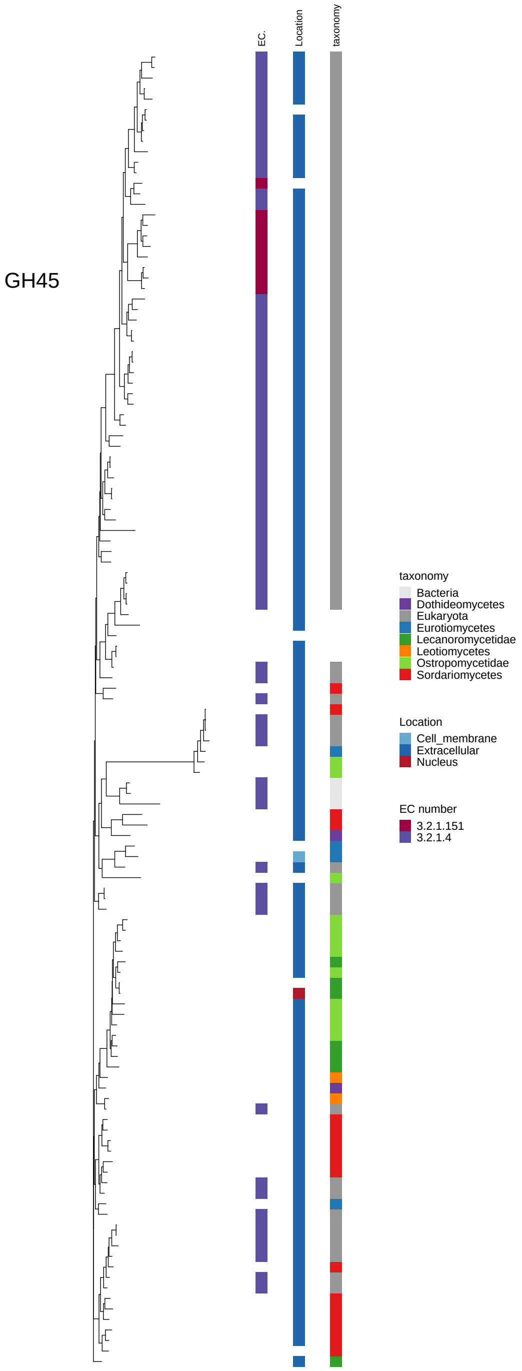

Supplementary Figure 44: Maximum-likelihood gene tree of the CAZyme family GH45. It includes all experimentally characterized sequences for that family downloaded from cazy.org as well as all sequences with GH45 annotations for in the 83 analyzed genomes including additional information where available.

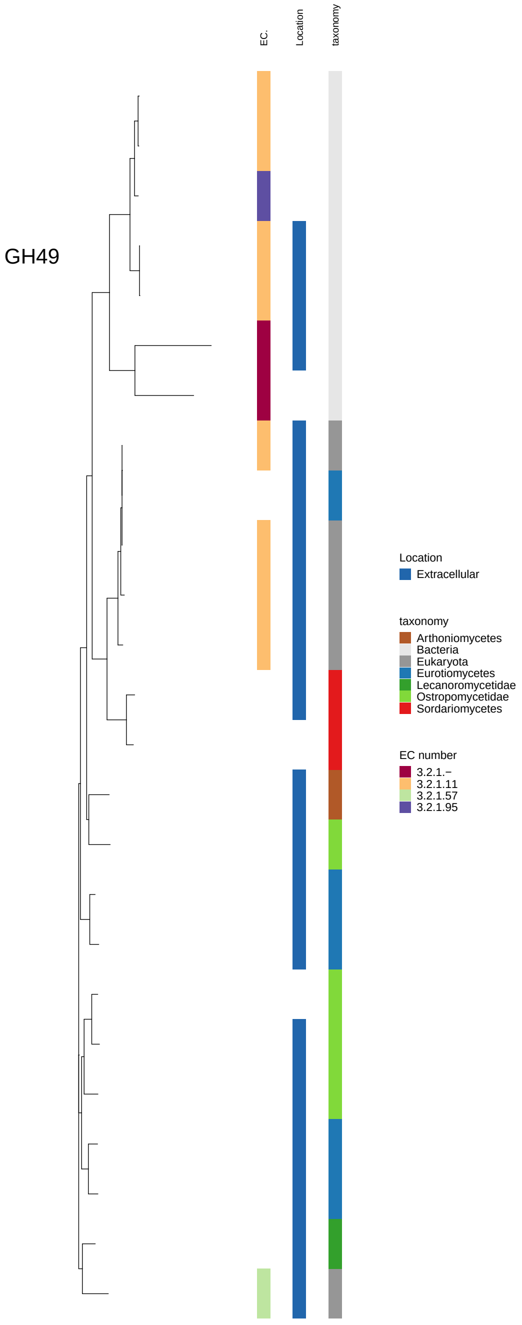

Supplementary Figure 45: Maximum-likelihood gene tree of the CAZyme family GH49. It includes all experimentally characterized sequences for that family downloaded from cazy.org as well as all sequences with GH49 annotations for in the 83 analyzed genomes including additional information where available.

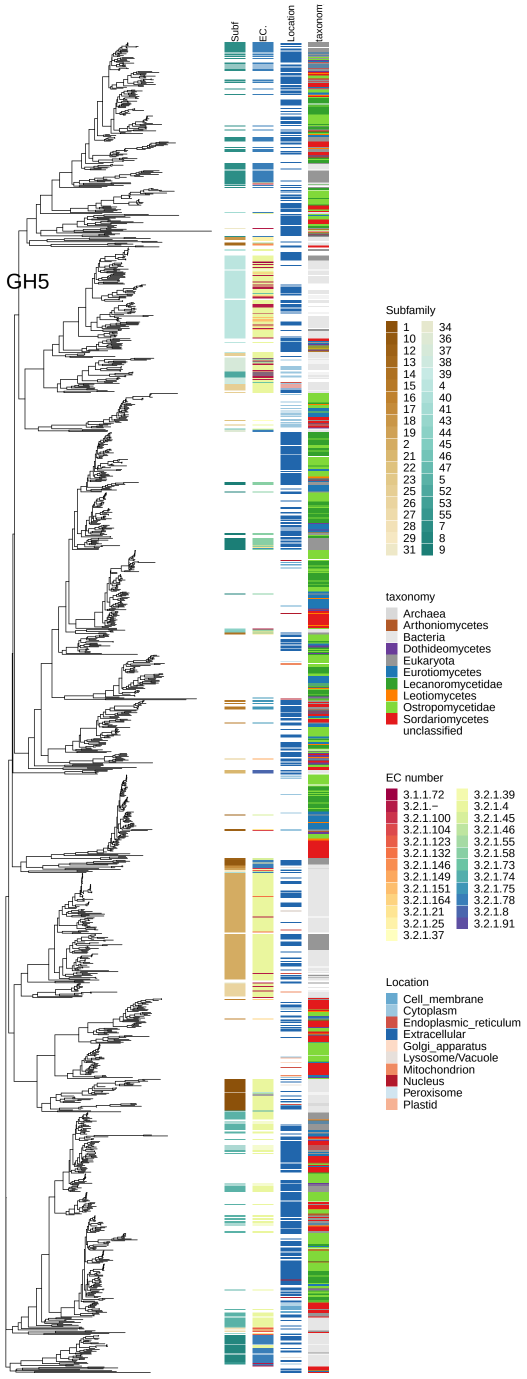

Supplementary Figure 46: Maximum-likelihood gene tree of the CAZyme family GH5. It includes all experimentally characterized sequences for that family downloaded from cazy.org as well as all sequences with GH5 annotations for in the 83 analyzed genomes including additional information where available.

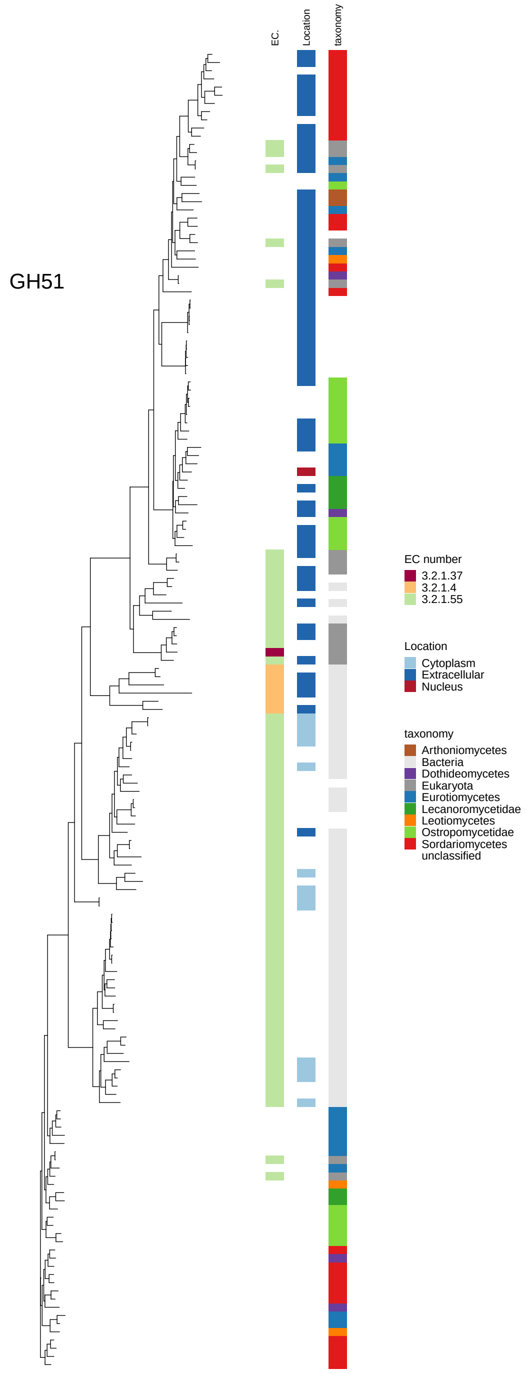

Supplementary Figure 47: Maximum-likelihood gene tree of the CAZyme family GH51. It includes all experimentally characterized sequences for that family downloaded from cazy.org as well as all sequences with GH51 annotations for in the 83 analyzed genomes including additional information where available.

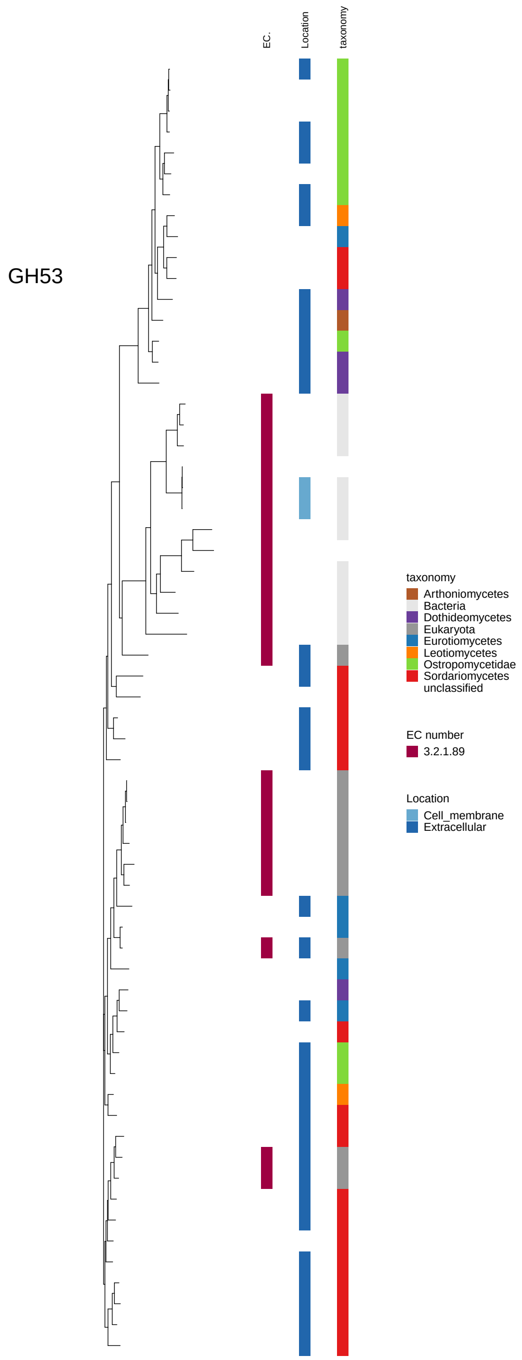

Supplementary Figure 48: Maximum-likelihood gene tree of the CAZyme family GH53. It includes all experimentally characterized sequences for that family downloaded from cazy.org as well as all sequences with GH53 annotations for in the 83 analyzed genomes including additional information where available.

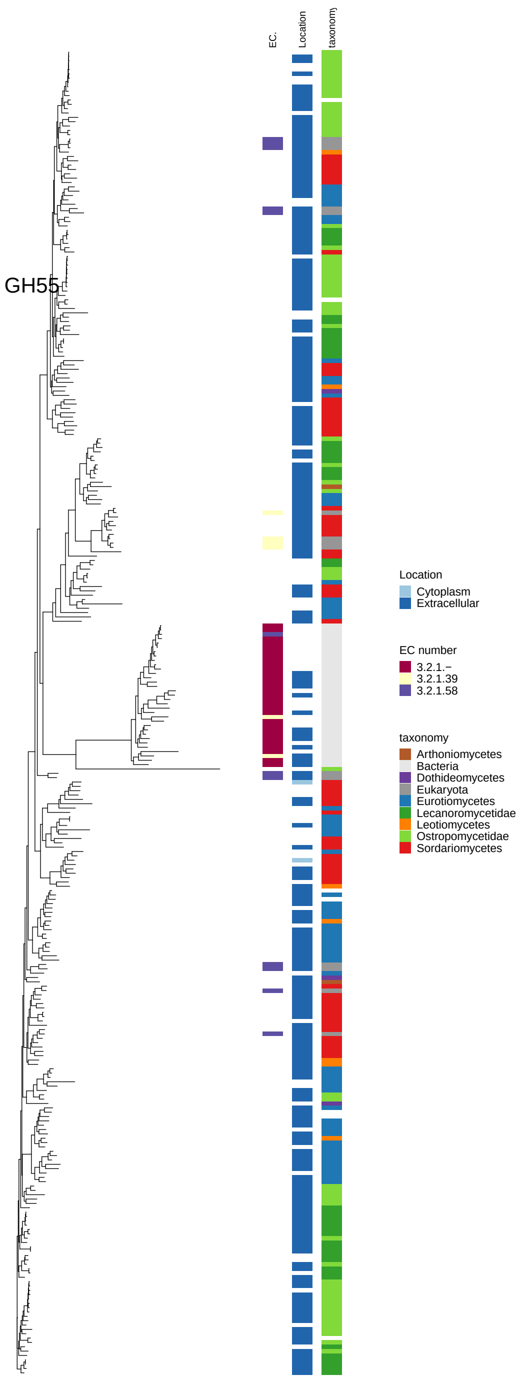

Supplementary Figure 49: Maximum-likelihood gene tree of the CAZyme family GH55. It includes all experimentally characterized sequences for that family downloaded from cazy.org as well as all sequences with GH55 annotations for in the 83 analyzed genomes including additional information where available.

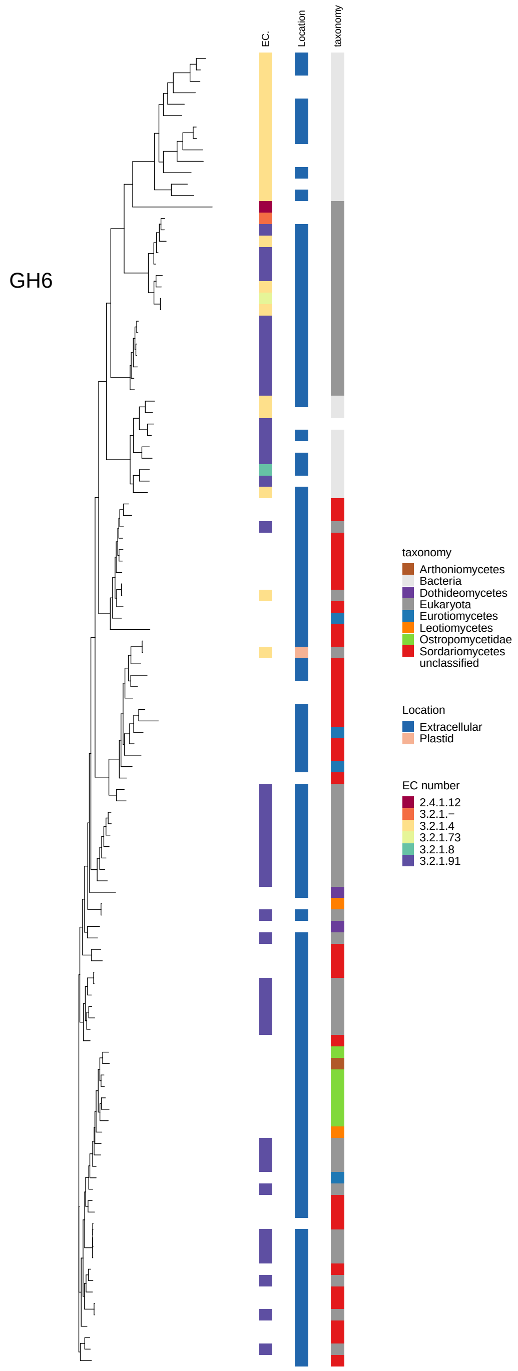

Supplementary Figure 50: Maximum-likelihood gene tree of the CAZyme family GH6. It includes all experimentally characterized sequences for that family downloaded from cazy.org as well as all sequences with GH6 annotations for in the 83 analyzed genomes including additional information where available.

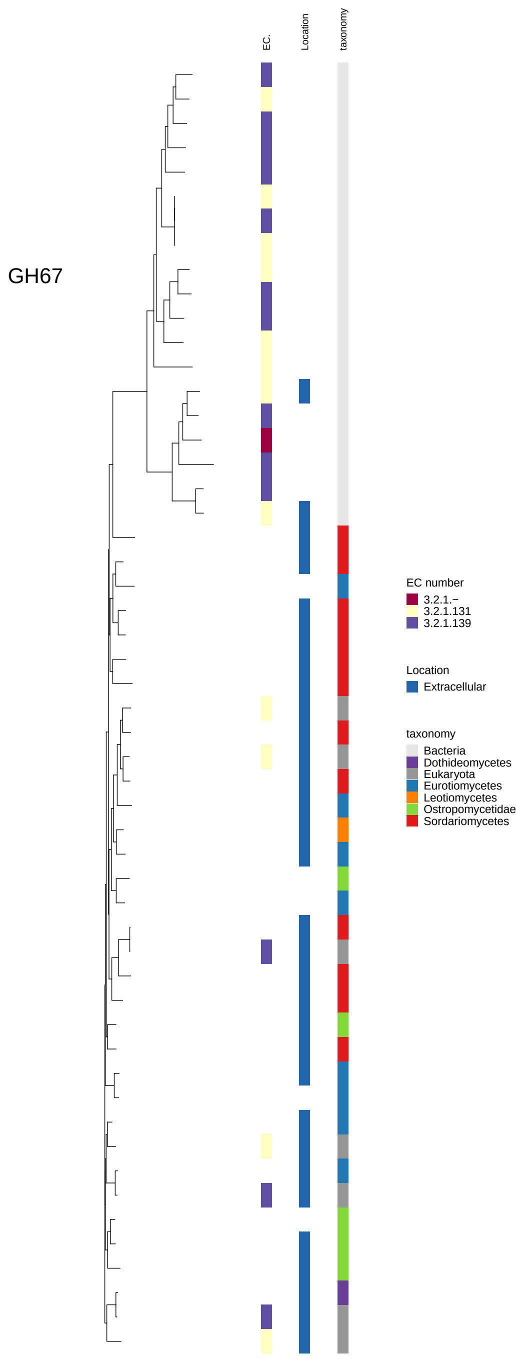

Supplementary Figure 51: Maximum-likelihood gene tree of the CAZyme family GH67. It includes all experimentally characterized sequences for that family downloaded from cazy.org as well as all sequences with GH67 annotations for in the 83 analyzed genomes including additional information where available.

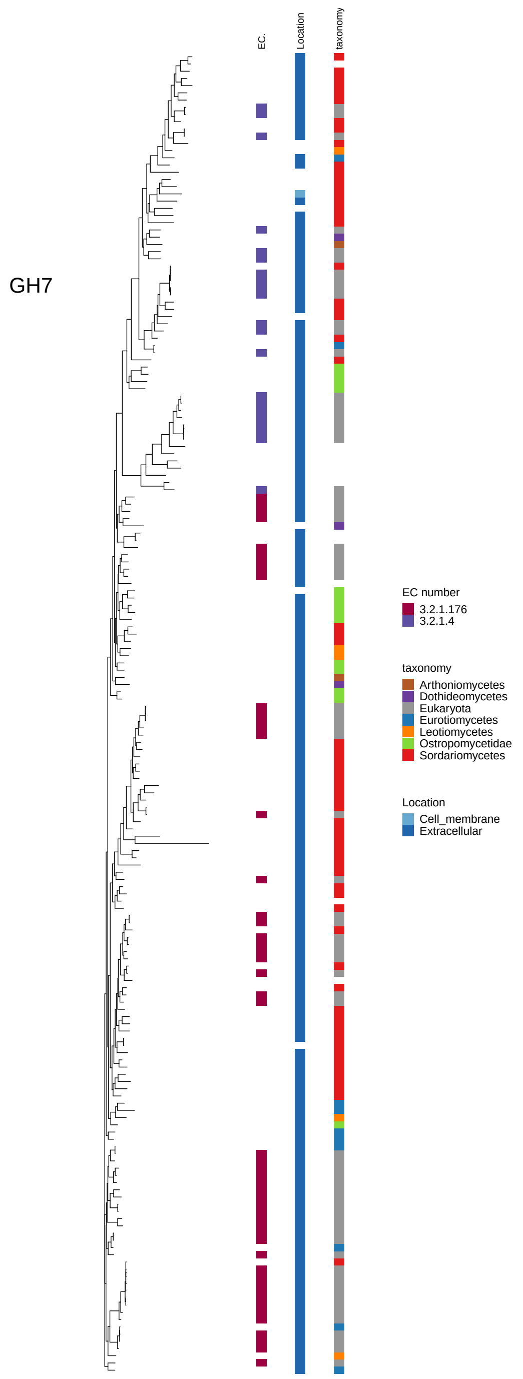

Supplementary Figure 52: Maximum-likelihood gene tree of the CAZyme family GH7. It includes all experimentally characterized sequences for that family downloaded from cazy.org as well as all sequences with GH7 annotations for in the 83 analyzed genomes including additional information where available.

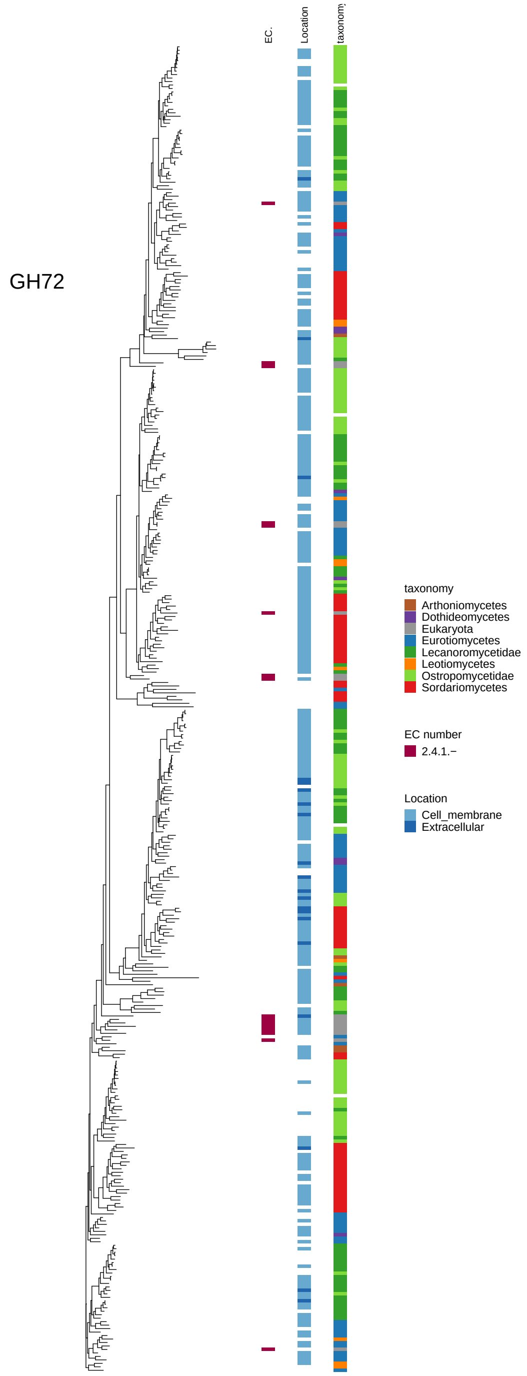

Supplementary Figure 53: Maximum-likelihood gene tree of the CAZyme family GH72. It includes all experimentally characterized sequences for that family downloaded from cazy.org as well as all sequences with GH72 annotations for in the 83 analyzed genomes including additional information where available.

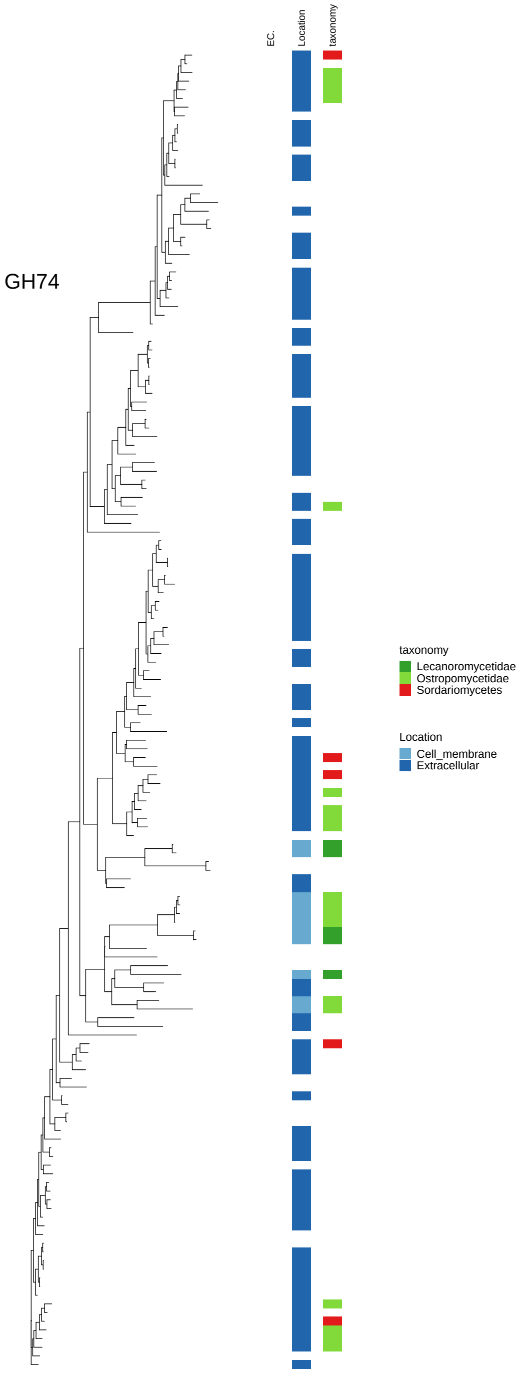

Supplementary Figure 54: Maximum-likelihood gene tree of the CAZyme family GH74. It includes all experimentally characterized sequences for that family downloaded from cazy.org as well as all sequences with GH74 annotations for in the 83 analyzed genomes including additional information where available.

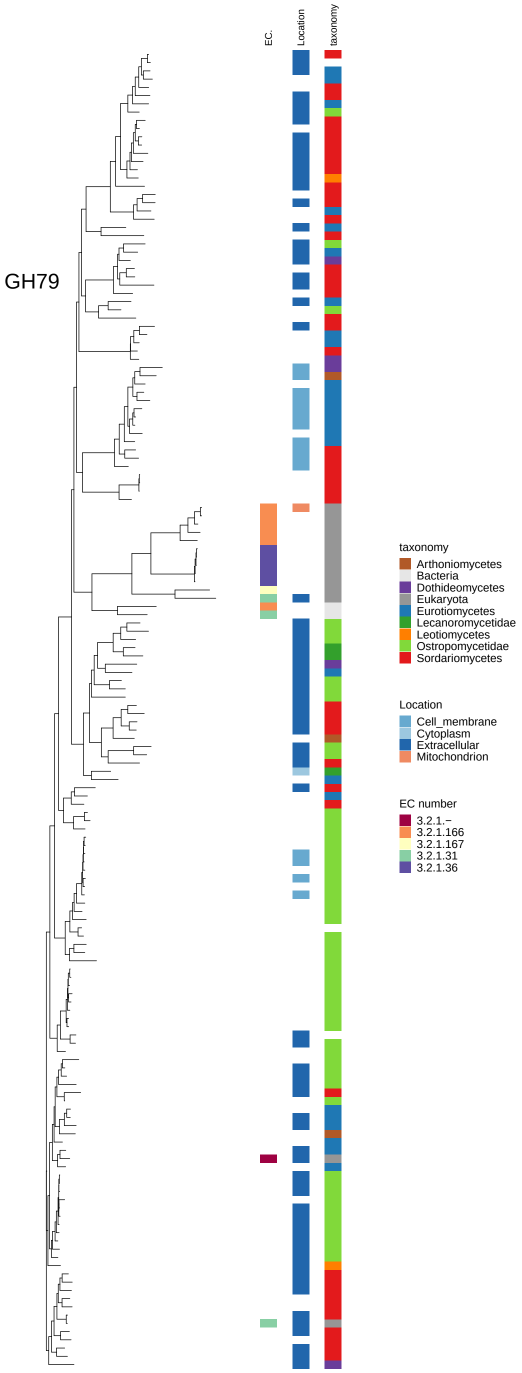

Supplementary Figure 55: Maximum-likelihood gene tree of the CAZyme family GH79. It includes all experimentally characterized sequences for that family downloaded from cazy.org as well as all sequences with GH79 annotations for in the 83 analyzed genomes including additional information where available.

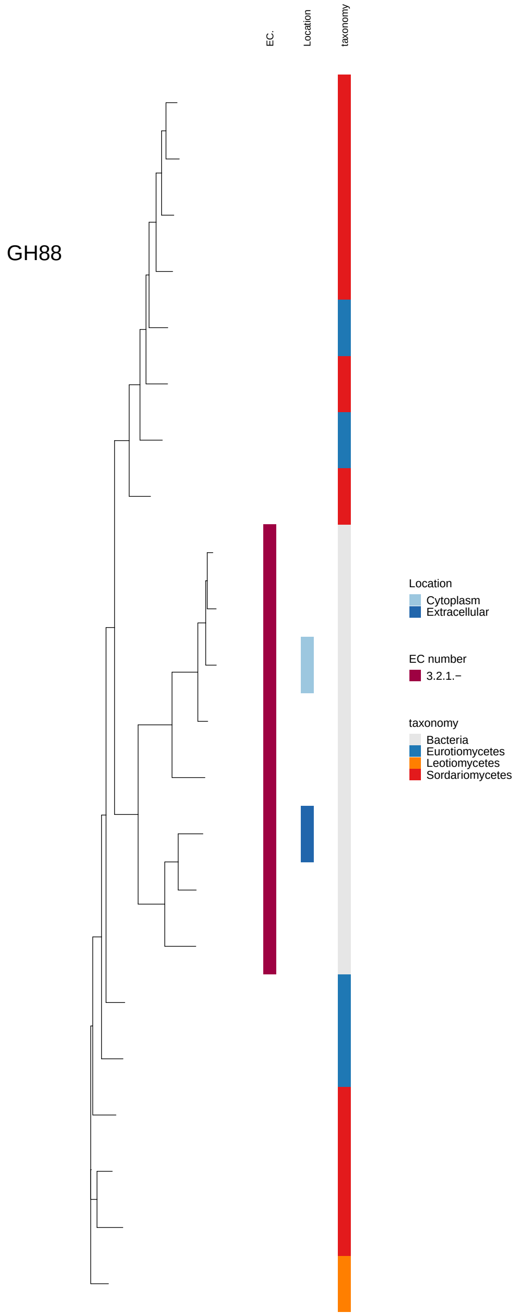

Supplementary Figure 56: Maximum-likelihood gene tree of the CAZyme family GH88. It includes all experimentally characterized sequences for that family downloaded from cazy.org as well as all sequences with GH88 annotations for in the 83 analyzed genomes including additional information where available.

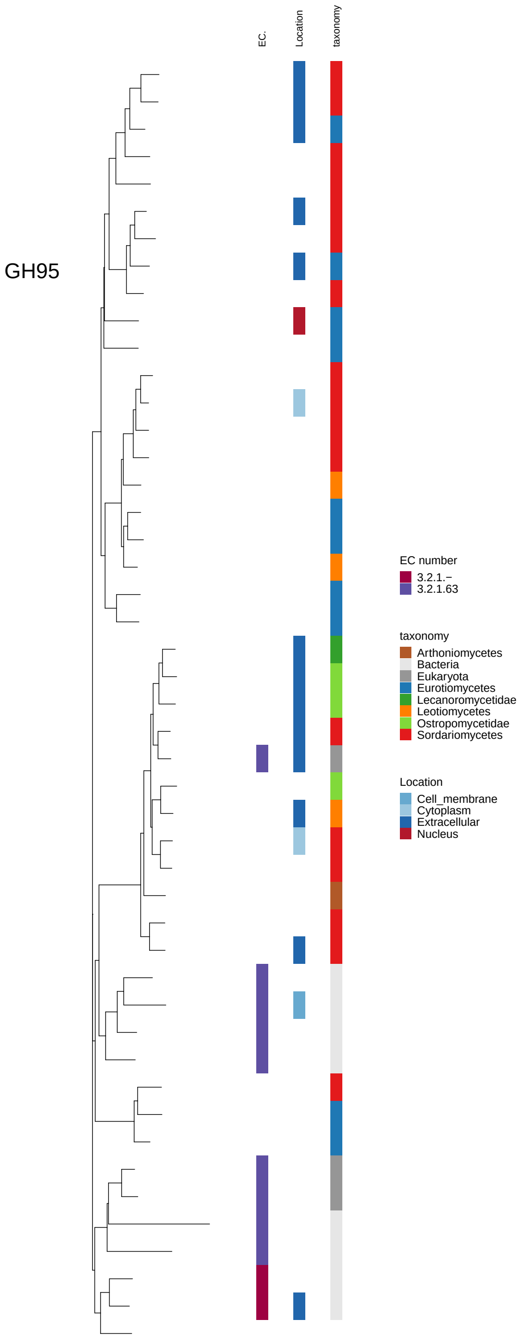

Supplementary Figure 57: Maximum-likelihood gene tree of the CAZyme family GH95. It includes all experimentally characterized sequences for that family downloaded from cazy.org as well as all sequences with GH95 annotations for in the 83 analyzed genomes including additional information where available.

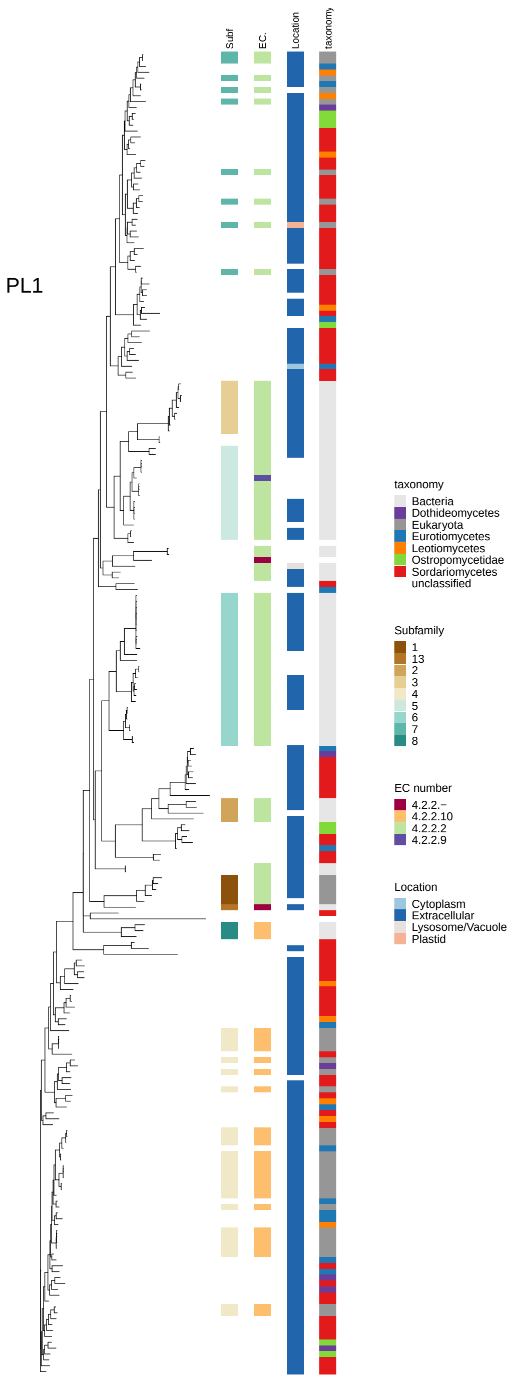

Supplementary Figure 58: Maximum-likelihood gene tree of the CAZyme family PL1. It includes all experimentally characterized sequences for that family downloaded from cazy.org as well as all sequences with PL1 annotations for in the 83 analyzed genomes including additional information where available.

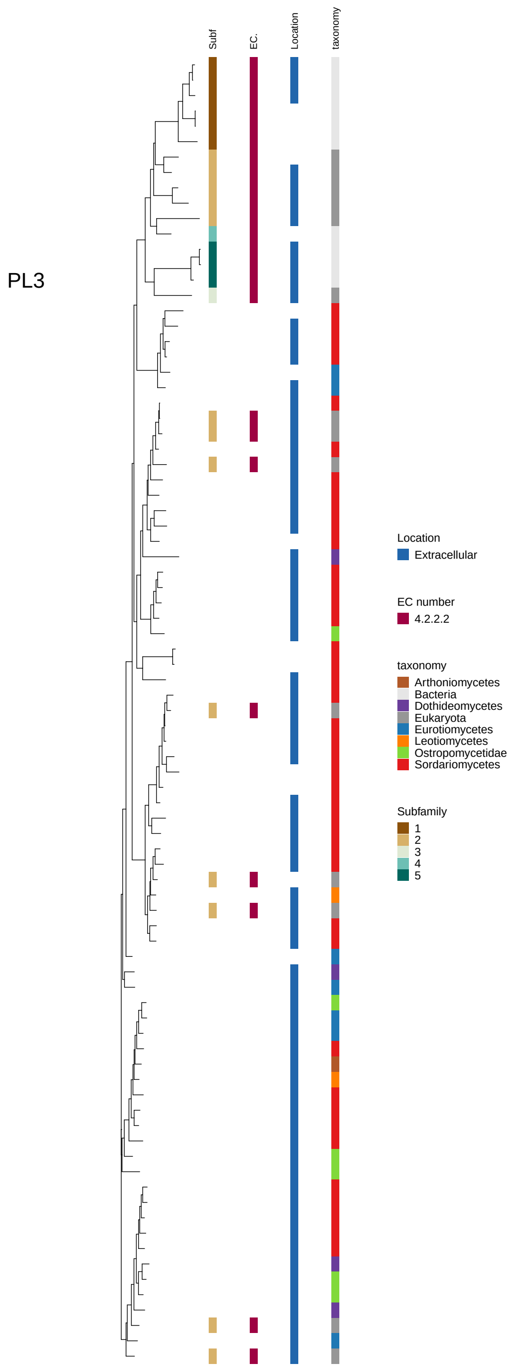

Supplementary Figure 59: Maximum-likelihood gene tree of the CAZyme family PL3. It includes all experimentally characterized sequences for that family downloaded from cazy.org as well as all sequences with PL3 annotations for in the 83 analyzed genomes including additional information where available.

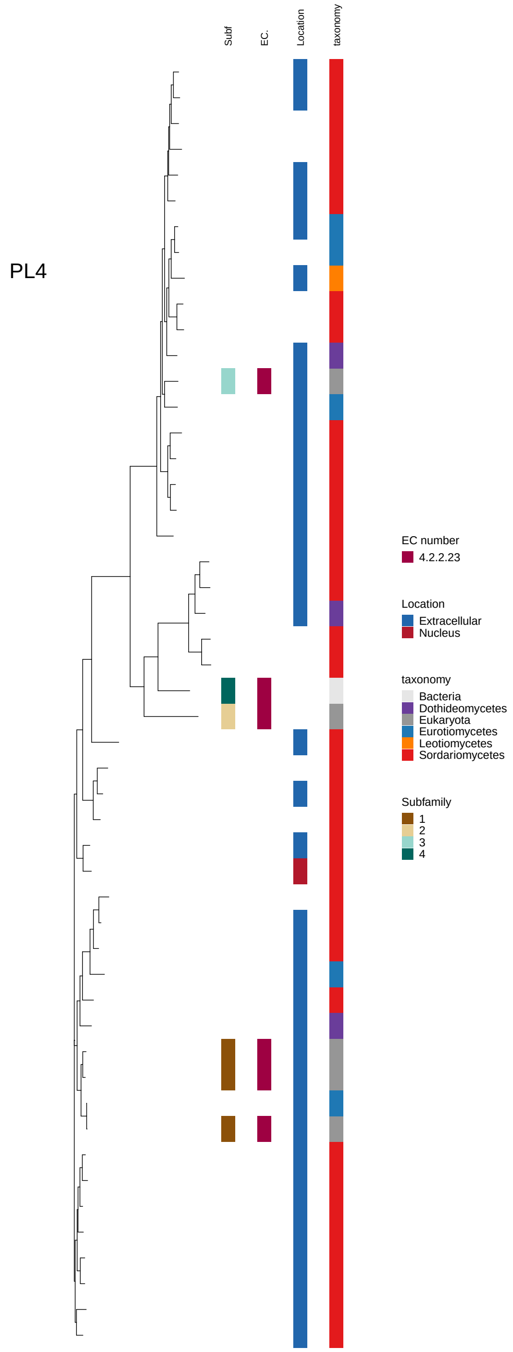

Supplementary Figure 60: Maximum-likelihood gene tree of the CAZyme family PL4. It includes all experimentally characterized sequences for that family downloaded from cazy.org as well as all sequences with PL4 annotations for in the 83 analyzed genomes including additional information where available.

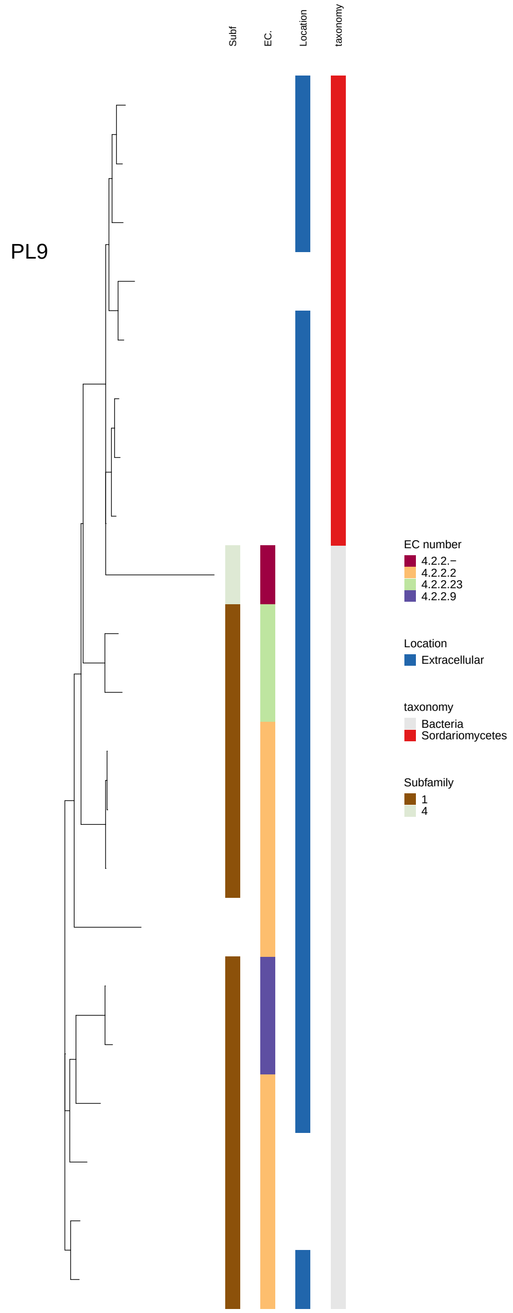

Supplementary Figure 61: Maximum-likelihood gene tree of the CAZyme family PL9. It includes all experimentally characterized sequences for that family downloaded from cazy.org as well as all sequences with PL9 annotations for in the 83 analyzed genomes including additional information where available.

References

1. Molina, M. del C. & Crespo, A. Comparison of development of axenic cultures of five species of lichen-forming fungi. *Mycological Research* (2000).

2. Pogoda, C. S., Keepers, K. G., Lendemer, J. C., Kane, N. C. & Tripp, E. A. Reductions in Complexity of Mitochondrial Genomes in Lichen-Forming Fungi Shed Light on Genome Architecture of Obligate Symbioses. *Molecular Ecology* (2018).

3. Hoang, D. T., biology, O. C. M. & 2017. UFBoot2: improving the ultrafast bootstrap approximation. *Molecular Biology and Evolution.*

4. Salichos, L. & Rokas, A. Inferring ancient divergences requires genes with strong phylogenetic signals. *Nature* (2013).

5. Minh, B. Q., Hahn, M. W. & Lanfear, R. New Methods to Calculate Concordance Factors for Phylogenomic Datasets. *Molecular Biology and Evolution* (2020).

6. Sanderson, M. J. r8s: inferring absolute rates of molecular evolution and divergence times in the absence of a molecular clock. *Bioinformatics* (2003).

7. Miyauchi, S. *et al.* Large-scale genome sequencing of mycorrhizal fungi provides insights into the early evolution of symbiotic traits. *Nature Communications* (2020).

8. Floudas, D. *et al.* The Paleozoic origin of enzymatic lignin decomposition reconstructed from 31 fungal genomes. *Science* (2012).

9. Revell, L. J. A comment on the use of stochastic character maps to estimate evolutionary rate variation in a continuously valued trait. *Systematic Biology* (2013).

10. Butler, M. A. & King, A. A. Phylogenetic comparative analysis: a modeling approach for adaptive evolution. *The American Naturalist* (2004).

11. Revell, L. J. Size-correction and principal components for interspecific comparative studies. *Evolution* (2009).

12. Wickham, H. *ggplot2: Elegant Graphics for Data Analysis.* (Springer-Verlag New York, 2016).

13. Henrissat, B., Claeyssens, M., Tomme, P., Lemesle, L. & Mornon, J. P. Cellulase Families Revealed by Hydrophobic Cluster-Analysis. *Gene* (1989).

14. Aspeborg, H., Coutinho, P. M., Wang, Y., Brumer, H. & Henrissat, B. Evolution, substrate specificity and subfamily classification of glycoside hydrolase family 5 (GH5). *BMC Evolutionary Biology* (2012).

15. Kohler, A. *et al.* Convergent losses of decay mechanisms and rapid turnover of symbiosis genes in mycorrhizal mutualists. *Nature Genetics* (2015).

16. Mewis, K., Lenfant, N., Lombard, V. & Henrissat, B. Dividing the Large Glycoside Hydrolase Family 43 into Subfamilies: a Motivation for Detailed Enzyme Characterization. *Applied and environmental Microbiology* (2016).

17. Emms, D. M. & Kelly, S. OrthoFinder: phylogenetic orthology inference for comparative genomics. (2019).

18. Mathé, C., Fawal, N., Roux, C. & Dunand, C. In silico definition of new ligninolytic peroxidase sub-classes in fungi and putative relation to fungal life style. *Scientific reports* (2019).

19. Huerta-Cepas, J. *et al.* Fast Genome-Wide Functional Annotation through Orthology Assignment by eggNOG-Mapper. *Molecular Biology and Evolution* (2017).

20. Wang, Y.-Y. *et al.* Genome characteristics reveal the impact of lichenization on lichen-forming fungus *Endocarpon pusillum* Hedwig (Verrucariales, Ascomycota). *BMC Genomics* (2014).

21. Ter-Hovhannisyan, V., Lomsadze, A., Chernoff, Y. O. & Borodovsky, M. Gene prediction in novel fungal genomes using an ab initio algorithm with unsupervised training. *Genome Research* (2008).

22. Park, S.-Y. *et al.* Draft genome sequence of *Umbilicaria muehlenbergii* KoLRILF000956, a lichen-Forming fungus amenable to genetic manipulation. *Genome Announcements* (2014).

23. Gurevich, A., Saveliev, V., Vyahhi, N. & Tesler, G. QUAST: quality assessment tool for genome assemblies. *Bioinformatics* (2013).

24. Resl, P. & Hahn, C. phylociraptor: rapid phylogenomic tree calculator. Available online: https://github.com/reslp/phylociraptor. (2021).

25. Price, M. N., Dehal, P. S. & Arkin, A. P. FastTree 2-Approximately Maximum-Likelihood Trees for Large Alignments. *PLoS ONE* (2010).

26. Stamatakis, A. RAxML version 8: A tool for Phylogenetic Analysis and Post-Analysis of Large Phylogenies. *Bioinformatics* (2014).

27. Edgar, R. C. MUSCLE: multiple sequence alignment with high accuracy and high throughput. *Nucleic Acids Research* (2004).

28. Stanke, M., Schöffmann, O., Morgenstern, B. & Waack, S. Gene prediction in eukaryotes with a generalized hidden Markov model that uses hints from external sources. *BMC Bioinformatics* (2006).

29. Majoros, W. H., Pertea, M. & Salzberg, S. L. TigrScan and GlimmerHMM: two open source ab initio eukaryotic gene-finders. *Bioinformatics* (2004).

30. Almagro Armenteros, J. J., Sønderby, C. K., Sønderby, S. K., Nielsen, H. & Winther, O. DeepLoc: prediction of protein subcellular localization using deep learning. *Bioinformatics* (2017).

31. Smit, A., Hubley, R. & Green, P. *RepeatMasker Open-4.0.* Available online: http://www.repeatmasker.org. (2013).

32. GitHub - reslp/binner: script for running multiple metagenome filtering programs using Docker containers. (2020).

33. Bankevich, A. *et al.* SPAdes: A new genome assembly algorithm and its applications to single-cell sequencing. *Journal of Computational Biology* (2012).

34. Smit, A. & Hubley, R. RepeatModeler Open-1.0. Available online: http://www.repeatmasker.org.

35. Zerbino, D. R. & Birney, E. Velvet: Algorithms for de novo short read assembly using de Bruijn graphs. *Genome Research* (2008).

36. Armaleo, D. *et al.* The lichen symbiosis re-viewed through the genomes of Cladonia grayi and its algal partner Asterochloris glomerata. *BMC Genomics* (2019).

37. Mirarab, S. *et al.* ASTRAL: genome-scale coalescent-based species tree estimation. *Bioinformatics* (2014).

38. Laetsch, D. R. & Blaxter, M. L. BlobTools: Interrogation of genome assemblies. *F1000Research* (2017).

39. Mendes, F. K., Vanderpool, D., Fulton, B. & Hahn, M. W. CAFE 5 models variation in evolutionary rates among gene families. *Bioinformatics* (2020).

40. Kajitani, R. *et al.* Efficient de novo assembly of highly heterozygous genomes from whole-genome shotgun short reads. *Genome Research* (2014).

41. Dal Grande, F. *et al.* The draft genome of the lichen-forming fungus Lasallia hispanica(Frey) Sancho & A. Crespo. *The Lichenologist* (2018).

42. McDonald, T. R., Mueller, O., Dietrich, F. S. & Lutzoni, F. High-throughput genome sequencing of lichenizing fungi to assess gene loss in the ammonium transporter/ammonia permease gene family. *BMC Genomics* (2013).

43. Katoh, K. & Standley, D. M. MAFFT multiple sequence alignment software version 7: improvements in performance and usability. *Molecular Biology and Evolution* (2013).

44. Waterhouse, R. M. *et al.* BUSCO Applications from Quality Assessments to Gene Prediction and Phylogenomics. *Molecular Biology and Evolution* (2017).

45. Korf, I. Gene finding in novel genomes. *BMC Bioinformatics* (2004).

46. Andrews, S. FastQC: a quality control tool for high throughput sequence data. Available online: http://www.bioinformatics.babraham.ac.uk/projects/fastqc. (2010).

47. Camacho, C. *et al.* BLAST+: architecture and applications. *BMC Bioinformatics* (2009).

48. Chikhi, R. & Rizk, G. Space-Efficient and Exact de Bruijn Graph Representation Based on a Bloom Filter. in *WABI* (2012).

49. Palmer, J. & Stajich, J. nextgenusfs/funannotate: funannotate v1.8.7. Available online: https://github.com/nextgenusfs/funannotate. (2021).

50. Sievers, F. *et al.* Fast, scalable generation of high-quality protein multiple sequence alignments using Clustal Omega. *Molecular Systems Biology* (2011).

51. Bolger, A. M., Lohse, M. & Usadel, B. Trimmomatic: a flexible trimmer for Illumina sequence data. *Bioinformatics* (2014).

52. Lowe, T. M. & Eddy, S. R. tRNAscan-SE: a program for improved detection of transfer RNA genes in genomic sequence. *Nucleic Acids Research* (1997).

53. Buchfink, B., Reuter, K. & Drost, H.-G. Sensitive protein alignments at tree-of-life scale using DIAMOND. *Nature Methods* (2021).

54. O'Connell, R. J. *et al.* Lifestyle transitions in plant pathogenic Colletotrichum fungi deciphered by genome and transcriptome analyses. *Nature Genetics* (2012).

55. Jackman, S. D. *et al.* ABySS 2.0: resource-efficient assembly of large genomes using a Bloom filter. *Genome Research* (2017).

56. Uritskiy, G. V., DiRuggiero, J. & Taylor, J. MetaWRAPa flexible pipeline for genome-resolved metagenomic data analysis. (2018).

57. Sharpton, T. J. *et al.* Comparative genomic analyses of the human fungal pathogens Coccidioides and their relatives. *Genome Research* (2009).

58. Jones, P. *et al.* InterProScan 5: genome-scale protein function classification. *Bioinformatics* (2014).

59. Capella-Gutierrez, S., Silla-Martinez, J. M. & Gabaldon, T. trimAl: a tool for automated alignment trimming in large-scale phylogenetic analyses. *Bioinformatics* (2009).

60. Jones, D. R. *et al.* SACCHARIS: an automated pipeline to streamline discovery of carbohydrate active enzyme activities within polyspecific families and de novo sequence datasets. *Biotechnology for Biofuels* (2018).

61. Minh, B. Q. *et al.* IQ-TREE 2: New Models and Efficient Methods for Phylogenetic Inference in the Genomic Era. *Molecular Biology and Evolution* (2020).

62. Mistry, J., Finn, R. D., Eddy, S. R., Bateman, A. & Punta, M. Challenges in homology search: HMMER3 and convergent evolution of coiled-coil regions. *Nucleic Acids Research* (2013).

63. Wang, Y., Yuan, X., Chen, L., Wang, X. & Li, C. Draft Genome Sequence of the Lichen-Forming Fungus Ramalina intermedia Strain YAF0013. *Genome Announcements* (2018).

64. Armstrong, E. E. *et al.* Draft Genome Sequence and Annotation of the Lichen-Forming Fungus Arthonia radiata. *Genome Announcements* (2018).

65. Staats, M. & Kan, J. A. L. van. Genome update of Botrytis cinerea strains B05.10 and T4. *Eukaryotic Cell* (2012).

66. Allen, J. L., McKenzie, S. K., Sleith, R. S. & Alter, S. E. First genome-wide analysis of the endangered, endemic lichen Cetradonia linearis reveals isolation by distance and strong population structure. *American Journal of Botany* (2018).

67. Park, S.-Y. *et al.* Draft Genome Sequence of Cladonia macilenta KoLRI003786, a Lichen-Forming Fungus Producing Biruloquinone. *Genome Announcements* (2013).

68. Park, S.-Y. *et al.* Draft Genome Sequence of Lichen-Forming Fungus Cladonia metacorallifera Strain KoLRI002260. *Genome Announcements* (2014).

69. Bertrand, R. L., Abdel-Hameed, M. & Sorensen, J. L. Lichen Biosynthetic Gene Clusters. Part I. Genome Sequencing Reveals a Rich Biosynthetic Potential. *Journal of natural products* (2018).

70. Raudabaugh, D. B. *et al.* Coniella lustricola, a new species from submerged detritus. *Mycological Progress* (2018).

71. Jiménez, D. J. *et al.* Draft Genome Sequence of Coniochaeta ligniaria NRRL 30616, a Lignocellulolytic Fungus for Bioabatement of Inhibitors in Plant Biomass Hydrolysates. *Genome Announcements* (2017).

72. Jahn, L. *et al.* *Journal of Biotechnology* (2017).

73. Li, S., Song, Q., Ji, P. & Cregan, P. Draft Genome Sequence of Phomopsis longicolla Type Strain TWH P74, a Fungus Causing Phomopsis Seed Decay in Soybean. *Genome Announcements* (2015).

74. Quandt, C. A. *et al.* Metagenome sequence of Elaphomyces granulatus from sporocarp tissue reveals Ascomycota ectomycorrhizal fingerprints of genome expansion and a Proteobacteria-rich microbiome. *Environmental Microbiology* (2015).

75. Jones, L. *et al.* Adaptive genomic structural variation in the grape powdery mildew pathogen, Erysiphe necator. *BMC Genomics* (2014).

76. Meiser, A., Otte, J. x000FC. rgen, Schmitt, I. & Grande, F. D. Sequencing genomes from mixed DNA samples - evaluating the metagenome skimming approach in lichenized fungi. *Scientific reports* (2017).

77. Ma, L.-J. *et al.* Comparative genomics reveals mobile pathogenicity chromosomes in Fusarium. *Nature* (2010).

78. Park, S.-Y. *et al.* Draft Genome Sequence of Lichen-Forming Fungus Caloplaca flavorubescens Strain KoLRI002931. *Genome Announcements* (2013).

79. Muñoz, J. F., McEwen, J. G., Clay, O. K. & Cuomo, C. A. Genome analysis reveals evolutionary mechanisms of adaptation in systemic dimorphic fungi. *Scientific reports* (2018).

80. Nicholson, M. J. *et al.* Draft Genome Sequence of the Filamentous Fungus Hypoxylon pulicicidum ATCC 74245. *Genome Announcements* (2018).

81. Tesei, D. *et al.* Draft Genome Sequences of the Black Rock Fungus Knufia petricola and Its Spontaneous Nonmelanized Mutant. *Genome Announcements* (2017).

82. Hüttner, S. *et al.* Combined genome and transcriptome sequencing to investigate the plant cell wall degrading enzyme system in the thermophilic fungus Malbranchea cinnamomea. *Biotechnology for Biofuels* (2017).

83. Yang, Y. *et al.* Complete genome sequence and transcriptomics analyses reveal pigment biosynthesis and regulatory mechanisms in an industrial strain, Monascus purpureus YY-1. *Scientific reports* (2015).

84. Huang, Y., Busk, P. K., Herbst, F.-A. & Lange, L. Genome and secretome analyses provide insights into keratin decomposition by novel proteases from the non-pathogenic fungus Onygena corvina. *Applied Microbiology and Biotechnology* (2015).

85. Specht, T., Dahlmann, T. A., Zadra, I., Kürnsteiner, H. & Kück, U. Complete Sequencing and Chromosome-Scale Genome Assembly of the Industrial Progenitor Strain P2niaD18 from the Penicillin Producer Penicillium chrysogenum. *Genome Announcements* (2014).

86. Morales-Cruz, A. *et al.* Distinctive expansion of gene families associated with plant cell wall degradation, secondary metabolism, and nutrient uptake in the genomes of grapevine trunk pathogens. *BMC Genomics* (2015).

87. Moreno, L. F. *et al.* Draft Genome Sequence of the Ant-Associated Fungus Phialophora attae (CBS 131958). *Genome Announcements* (2015).

88. Espagne, E. *et al.* The genome sequence of the model ascomycete fungus Podospora anserina. *Genome Biol* (2008).

89. Nowrousian, M. *et al.* De novo assembly of a 40 Mb eukaryotic genome from short sequence reads: Sordaria macrospora, a model organism for fungal morphogenesis. *PLoS Genetics* (2010).

90. Fujii, T., Koike, H., Sawayama, S., Yano, S. & Inoue, H. Draft Genome Sequence of Talaromyces cellulolyticus Strain Y-94, a Source of Lignocellulosic Biomass-Degrading Enzymes. *Genome Announcements* (2015).

91. Wingfield, B. D. *et al.* Draft genome sequences of Ceratocystis eucalypticola, Chrysoporthe cubensis, C. deuterocubensis, Davidsoniella virescens, Fusarium temperatum, Graphilbum fragrans, Penicillium nordicum, and Thielaviopsis musarum. *IMA fungus* (2015).

92. Martinez, D. *et al.* Genome sequencing and analysis of the biomass-degrading fungus Trichoderma reesei (syn. Hypocrea jecorina). *Nature biotechnology* (2008).

93. Sook-Young, P. *et al.* Draft Genome Sequence of Xylaria grammica EL000614, a Strain Producing Grammicin, a Potent Nematicidal Compound. *Mycobiology* (2021).

94. Wang, Y., Wei, X., Huang, J. & Wei, J. Modification and functional adaptation of the MBF1 gene family in the lichenized fungus Endocarpon pusillum under environmental stress. *Scientific reports* (2017).

95. Wang, N.-Y., Zhang, K., Huguet-Tapia, J. C., Rollins, J. A. & Dewdney, M. M. Mating Type and Simple Sequence Repeat Markers Indicate a Clonal Population of Phyllosticta citricarpa in Florida. *Phytopathology* (2016).

96. Zhang, G., Wang, D. & Wei, G. De Novo Sequencing and Analysis of the Whole Genome of Aureobasidium pullulans CCTCC M 2012259. *Genomics and Applied Biology* (2017).

97. Teixeira, M. M. *et al.* Exploring the genomic diversity of black yeasts and relatives (Chaetothyriales, Ascomycota). *Studies in Mycology* (2017).

98. Schardl, C. L. *et al.* Currencies of mutualisms: sources of alkaloid genes in vertically transmitted epichloae. *Toxins* (2013).

99. Vicente, V. A. *et al.* Comparative Genomics of Sibling Species of Fonsecaea Associated with Human Chromoblastomycosis. *Frontiers in Microbiology* (2017).

100. Song, X.-S. *et al.* A myosin5 dsRNA that reduces the fungicide resistance and pathogenicity of Fusarium asiaticum. *Pesticide Biochemistry and Physiology* (2018).

101. Sayari, M. *et al.* Characterization of the Ergosterol Biosynthesis Pathway in Ceratocystidaceae. *Journal of fungi* (2021).

102. Merlotti, A., Faria do Valle, I., Castellani, G. & Remondini, D. Statistical modelling of CG interdistance across multiple organisms. *BMC Bioinformatics* (2018).

103. Tagirdzhanova, G., McCutcheon, J. P. & Spribille, T. Lichen fungi do not depend on the alga for ATP production: A comment on Pogoda et al. (2018). *Molecular Ecology* (2021).
