# Supplementary material for: Large differences in carbohydrate degradation and transport potential among lichen fungal symbionts
Source: Nat Commun. 2022 May 12;13:2634. doi: 10.1038/s41467-022-30218-6 (PMC9098629; doi:10.1038/s41467-022-30218-6)
Supplement: Supplementary file 2 — Reporting Summary [file 41467_2022_30218_MOESM2_ESM.pdf]

nature portfolio

Corresponding author(s): Prof. Toby Spribille

Last updated by author(s): Apr 12, 2022

Reporting Summary

Nature Portfolio wishes to improve the reproducibility of the work that we publish. This form provides structure for consistency and transparency in reporting. For further information on Nature Portfolio policies, see our Editorial Policies and the Editorial Policy Checklist.

Statistics

For all statistical analyses, confirm that the following items are present in the figure legend, table legend, main text, or Methods section.

n/a	Confirmed	
☐	☒	The exact sample size (*n*) for each experimental group/condition, given as a discrete number and unit of measurement
☒	☐	A statement on whether measurements were taken from distinct samples or whether the same sample was measured repeatedly
☐	☒	The statistical test(s) used AND whether they are one- or two-sided<br>*Only common tests should be described solely by name; describe more complex techniques in the Methods section.*
☒	☐	A description of all covariates tested
☒	☐	A description of any assumptions or corrections, such as tests of normality and adjustment for multiple comparisons
☐	☒	A full description of the statistical parameters including central tendency (e.g. means) or other basic estimates (e.g. regression coefficient) AND variation (e.g. standard deviation) or associated estimates of uncertainty (e.g. confidence intervals)
☐	☒	For null hypothesis testing, the test statistic (e.g. *F*, *t*, *r*) with confidence intervals, effect sizes, degrees of freedom and *P* value noted<br>*Give P values as exact values whenever suitable.*
☒	☐	For Bayesian analysis, information on the choice of priors and Markov chain Monte Carlo settings
☒	☐	For hierarchical and complex designs, identification of the appropriate level for tests and full reporting of outcomes
☒	☐	Estimates of effect sizes (e.g. Cohen's *d*, Pearson's *r*), indicating how they were calculated

Our web collection on statistics for biologists contains articles on many of the points above.

Software and code

Policy information about availability of computer code

Data collection	The authors did not use any software during data collection
Data analysis	All code used to analyze data in this manuscript is deposited at GitHub (https://github.com/reslp/LFS-cazy-comparative; doi:10.5281/zenodo.6453144).<br>Filtering of Raw sequence data and genome assembly was done individually for each sample. Parameters and software used are given in supplementary table 4 and 5.<br>Used software is containerized (Docker, Singularity) and version controlled and publicly available on Dockerhub. Version numbers for each software are as follows and also given in Suppl. Table 3.<br>trimmomatic 0.38<br>FastQC 0.11.7<br>binner 0.1<br>SPAdes 3.12.0<br>platanus 1.2.4<br>minia3 git commit 1d5b8f4<br>abyss 2.0.1<br>velvet 1.2.0<br>QUAST 4.6.3<br>BUSCO 3.0.2, 4.0.2<br>blobtools 1.1.1<br>MetaWrap need to add<br>CONCOCT 1.2<br>ncbi-blast+ 2.9.0

funannotate 1.8.3
RepeatModeller 1.0.11
RepeatMasker 4.0.7
Augustus 3.3.2
GlimmerHMM 3.0.4
snap 2006-07-28
GeneMark-ES 4.62 (Jan. 2020)
tRNA-Scan 2.0.5
InterproScan 5.48-83.0
eggnog-mapper 1.0.3
phylociraptor git commit a93b4c8
mafft 7.464
trimAL 1.4.1
IQ-Tree 2.0.7
ASTRAL 5.7.1
r8s 1.81
CAFE 5.0.0b2
Saccharis git commit 9a748be
MUSCLE 3.8.31
hmmer 3.1b2
raxml 8.2.12
DeepLoc 1.0
Clustal Omega 1.2.4
Orthofinder 2.5.2
diamond 0.9.24, 0.9.22
Fasttree 2.1.10
phytools 0.7-70
ggplot2 3.3.3
ggtree 2.4.2

For manuscripts utilizing custom algorithms or software that are central to the research but not yet described in published literature, software must be made available to editors and reviewers. We strongly encourage code deposition in a community repository (e.g. GitHub). See the Nature Portfolio guidelines for submitting code & software for further information.

Data

Policy information about availability of data

All manuscripts must include a data availability statement. This statement should provide the following information, where applicable:
- Accession codes, unique identifiers, or web links for publicly available datasets
- A description of any restrictions on data availability
- For clinical datasets or third party data, please ensure that the statement adheres to our policy

De-novo generated genome assemblies and corresponding functional annotations are deposited at NCBI under BioProject PRJNA795879 [https://www.ncbi.nlm.nih.gov/bioproject/PRJNA795879]. Accession numbers for all genomes used in this study are provided in supplementary data 1. Used PFAM and RedoxiBase sequences, annotations of previously sequenced genomes, alignments and phylogenomic trees are available for download in a public Dryad repository (doi:10.5061/dryad.3xsj3txjb). Source Data are provided with this paper.

Field-specific reporting

Please select the one below that is the best fit for your research. If you are not sure, read the appropriate sections before making your selection.

☐ Life sciences ☐ Behavioural & social sciences ☒ Ecological, evolutionary & environmental sciences

For a reference copy of the document with all sections, see nature.com/documents/nr-reporting-summary-flat.pdf

Ecological, evolutionary & environmental sciences study design

All studies must disclose on these points even when the disclosure is negative.

Study description	This study is a comparative genomic study of 83 fungal genomes. It uses phylogenomic approaches and phylogenetic comparative methods to understand the evolution of carbohydrate active enzymes in lichen fungal symbionts.
Research sample	The research sample includes 83 fungal genomes from of Lecanoromycetes (the largest radiation of lichen-fungal symbionts) and its sister group Eurotiomycetes. Rationale: This sample is a representative cross-section of growth types and colonized substratum types in the Lecanoromycete in-group as well as a representative sample of species with different lifestyles in the Eurotiomycetes outgroup.
Sampling strategy	No statistical methods were used to determine sampling size a priori. Sampling was based on sample availability.

Data collection	Illumina sequencing data for all de-novo sequenced genomes was produced between 2014 and 2019. DNA for individual samples was extracted in the labs of John McCutcheon (Agyrium rufum, Lambiella insularis, Trapelia coarctata, Xylographa parallela, Xylographa pallens, Loxospora ochrophaea, Schaereria dolodes), Silke Werth (Lobaria immixta, Pseudocyphellaria aurata, Xylographa opegraphella, Xylographa soralifera, Xylographa bjoerkii and Xylographa trunciseda), Toby Spribille (Acarospora aff strigata, Bachmanniomyces sp. TS44760, Hypocenomyce scalaris, Lignoscripta atroalba, Mycoblastus sanguinarius, Puttea exsequens, Thelotrema lepadinum, Toensbergia leuccococca, Varicellaria rhodocarpa, Xylographa carneopallida), Mats Wedin (Stictis urceolatum) and Ólafur Sigmar Andrésson (Peltigera leucophlebia and Sticta canariensis). Illumina sequencing was performed in different sequencing facilities, details are given in the Supplementary material document.<br>Data regarding heterologous expression experiments were derived by reading them off of a spectrophotometer and entering them into an Excel spreadsheet in the lab of Lisa Willis.
Timing and spatial scale	Lichen tissue cultures and DNA were extracted from herbarium derived samples (voucher information given in Supplementary Information). For each lichen a single specimen was used as representative for the fungal symbiotic partner.
Data exclusions	All produced data was included in the analyses.
Reproducibility	Reproducibility of bioinformatic analyses is ensured by version controlled containerized (Docker, Singularity) open-source software that is publicly available. All scripts and custom code is open-source and publicly available on Github. All attempts to repeat bioinformatic analyses were successful. All enzymatic assays were reproducible with different batches of protein and experiments were performed in technical and biological triplicates.
Randomization	This study is a comparative genomic study investigating differences between multiple fungal genomes. This does not involve randomization or grouping of samples.
Blinding	None of the experiments could be influenced at any stage nor did they have known a priori outcomes. No additional blinding measures were required.

Did the study involve field work? ☐ Yes ☒ No

Reporting for specific materials, systems and methods

We require information from authors about some types of materials, experimental systems and methods used in many studies. Here, indicate whether each material, system or method listed is relevant to your study. If you are not sure if a list item applies to your research, read the appropriate section before selecting a response.

Materials & experimental systems

n/a	Involved in the study	
☒	☐	Antibodies
☒	☐	Eukaryotic cell lines
☒	☐	Palaeontology and archaeology
☒	☐	Animals and other organisms
☒	☐	Human research participants
☒	☐	Clinical data
☒	☐	Dual use research of concern

Methods

n/a	Involved in the study	
☒	☐	ChIP-seq
☒	☐	Flow cytometry
☒	☐	MRI-based neuroimaging
