# Supplementary material for: Large differences in carbohydrate degradation and transport potential among lichen fungal symbionts
Source: Nat Commun. 2022 May 12;13:2634. doi: 10.1038/s41467-022-30218-6 (PMC9098629; doi:10.1038/s41467-022-30218-6)
Supplement: Supplementary file 4 — Description of Additional Supplementary Files [file 41467_2022_30218_MOESM4_ESM.pdf]

Title: Supplementary Data 1:
Description: Information on all genomes included in this study.

Title: Supplementary Data 2:
Description: Sample information of denovo sequenced genomes.

Title: Supplementary Data 3:
Description: Overview of single-copy genes and alignments used for phylogenomic reconstruction.
